# Stability of Stochastically Forced Solitons in the Korteweg-de Vries Equation

**DOI:** 10.1007/s40072-026-00415-1

**Published:** 2026-02-20

**Authors:** R. W. S. Westdorp, H. J. Hupkes

**Affiliations:** Mathematisch Instituut, Universiteit Leiden, P.O. Box 9512, 2300 RA Leiden, The Netherlands

**Keywords:** Stochastic traveling waves, Korteweg-de Vries equation, Stability, Forcing, 60H15, 35Q53, 35C08

## Abstract

We study the stability and dynamics of solitons in the Korteweg-de Vries (KdV) equation in the presence of noise and deterministic forcing. The noise is space-dependent and statistically translation-invariant. We show that, for small forcing, solitons remain close to the family of traveling waves in a weighted Sobolev norm, with high probability. We study the effective dynamics of the soliton amplitude and position via their variational phase, for which we derive explicit modulation equations. The stability result holds on a time scale where the deterministic forcing induces significant amplitude modulation.

## Introduction

This paper studies the stochastic KdV equation

1.1 $$\begin{aligned} {\mathrm d}u = -(\partial _x^3 u +2 u\partial _x u)\ {\mathrm d}t+\epsilon f(t)u \ {\mathrm d}t+\sigma u\ {\mathrm d}W^Q_t, \end{aligned}$$

where *u* is a real-valued process on $(t,x)\in {\mathbb {R}}^+\times {\mathbb {R}}$. The scalar parameters ϵ>0 and σ>0 introduce deterministic and random multiplicative forcing to the KdV dynamics, respectively. The noise process $W_t^Q$ is a Wiener process taking values in $L^2({\mathbb {R}})$. The noise is white in time and colored in space with translation-invariant statistics given by the formal identity

$$\begin{aligned} \mathbb {E}[W^Q(t,x)W^Q(s,y)]=q(x-y)(t\wedge s), \quad x,y\in {\mathbb {R}}, \quad t,s>0,\end{aligned}$$

with $q\in H^1({\mathbb {R}})\cap L^1({\mathbb {R}})$. The deterministic forcing depends only on time, prescribed by the scalar-valued function $f:{\mathbb {R}}^+\rightarrow {\mathbb {R}}$. We study the effect of this forcing on the family of solitary waves associated to the unforced KdV equation ((1.1) with $\epsilon =\sigma =0$). In particular, we establish the stability of forced solitons on time scales where the forcing $\epsilon , \sigma >0$ causes drastic amplitude modulation. This paper complements our formal analysis in [27] with a rigorous stability result, and extends our deterministic results in [28] to space-dependent noise.

The Korteweg-de Vries equation is a well-known nonlinear dispersive PDE originally introduced as a model for shallow-water waves [4, 17]. It famously possesses a family of right-traveling wave solutions $u(t,x)=\phi _c(x-ct)$ with velocity dependent wave-profiles explicitly given by

1.2 $$\begin{aligned} \phi _c(x)=\frac{3c}{2}{{\,\textrm{sech}\,}}^2(\sqrt{c}x/2), \quad c>0. \end{aligned}$$

Note that the velocity dependence of the wave profiles satisfies the simple scaling $\phi _c(x)=c\phi _1(\sqrt{c}x)$. The traveling waves (1.2), or solitons, arise due to a balance of dispersion and nonlinear effects. They are of key importance for the KdV dynamics: inverse scattering theory provides exact solutions to the KdV equation in terms of right-traveling solitons and a remaining dispersive component [13].

Many variations on (1.1) have been introduced in the literature to incorporate various (random) forcing mechanisms. In particular, we mention [16], which first considered the stochastic KdV equation. We refer to our previous works [27, 28] and references therein for more background on stochastic forcing and deterministic forcing, respectively, in the context of the KdV equation.

**Deterministic stability** The stability of solitons (1.2) with respect to an initial perturbation has been extensively studied, for instance, by energy methods [21]. These rely on the conservative nature of the KdV dynamics: an infinite number of ‘integrals of motion’, such as the $L^2$-norm, is conserved under the KdV flow [22]. The family (1.2) is, as a consequence of dispersion, only marginally linearly stable in $L^2$. Indeed, the linearized dynamics around the soliton $\phi _c$ are detailed by the operator

1.3 $$\begin{aligned} {\mathcal {L}}_c=-\partial _x^3+c\partial _x-2\partial _x(\phi _c\cdot ), \end{aligned}$$

which, viewed as an operator on $L^2$, has spectrum $i{\mathbb {R}}$. In this work we rely heavily on the stability theory for the exponentially weighted spaces

1.4 $$\begin{aligned} L^2_w({\mathbb {R}})=&\big \{g: e^{wx}g\in L^2({\mathbb {R}})\big \}\quad {\text {with}} \quad \Vert g\Vert _{L^2_w}=\Vert e^{wx}g\Vert _{L^2}, \end{aligned}$$

1.5 $$\begin{aligned} H^1_w({\mathbb {R}})=&\big \{g: e^{wx}g\in H^1({\mathbb {R}})\big \}\quad {\text {with}} \quad \Vert g\Vert _{H^1_w}=\Vert e^{wx}g\Vert _{H^1} \end{aligned}$$

that was developed by Pego and Weinstein in the classic work [25]. The exponential weight $e^{wx}$ (w>0) shifts the continuous spectrum of the operator ${\mathcal {L}}_c$ with $\sqrt{c}>w$ into the stable half-plane, leaving a spectral gap of size $w(c-w^2)$ and a double eigenvalue at 0. Physically, the exponential weight dampens persisting disturbances outrun by the soliton. The linearized dynamics contain two neutral modes, associated with infinitesimal changes of $\phi _c$ with respect to the space variable *x* and the amplitude parameter *c*. These spectral properties ensure that the linear flow $\{e^{{\mathcal {L}}_ct}\}_{t\ge 0}$ on $L^2_w$ generated by the operator (1.3) is exponentially stable on a subspace where the neutral eigenvalue is avoided. This subspace of $L^2_w$ consists of functions *v* that satisfy the orthogonality conditions

1.6 $$\begin{aligned} \langle v,\phi _c\rangle =\langle v,\zeta _c\rangle =0, \end{aligned}$$

where

$$\begin{aligned} \zeta _c(x)=\int _{-\infty }^x\partial _c\phi _c(y){\mathrm d}y. \end{aligned}$$

Based on these properties, Pego and Weinstein [25] established the orbital stability of the traveling wave family (1.2) with respect to a small initial perturbation in $H^1\cap H^1_w$. In fact, such an initial perturbation only causes a small asymptotic phase-shift and amplitude change in the soliton.

**Stochastic stability** Stability of the wave family (1.2) with respect to small stochastic forcing has been previously considered in [10]. Based on energy methods, de Bouard and Debussche show that solutions to (1.1) with ϵ=0 and small σ>0 stay close to the wave family for times small with respect to $\sigma ^{-2}$. Their method relies on the fact that the soliton amplitude remains close to its starting value on this time scale. This is a significant restriction, as the result does not cover any (significant) modulation of the soliton. These stochastic modulations have, however, been explored to some degree in a formal sense. For example, the work [5] uses a collective coordinate approach to treat the case where $W_t^Q$ is replaced by a scalar Brownian motion. The numerical and analytical results in [27] cover both scalar and space-dependent noise and provide explicit Taylor expansions for the behavior of the modulation parameters.

**Present work** In the present work, we consider deterministic and stochastic forcing of the soliton family (1.2), leading to stochastic modulations of the amplitude and position over time. We establish the orbital stability of (1.2) in a setting where the stochastic amplitude modulations can have arbitrary size. This is primarily due to the deterministic forcing mechanism (ϵ>0) present in (1.1), which increases/decreases the energy present in the system after time *t* by a factor $e^{\epsilon \int _0^t f(s){\mathrm d}s}$ [28]. In this setting, it is vital to explicitly account for the dynamics of the soliton amplitude. In particular, we will show that, to leading order in the small parameters ϵ and σ, this amplitude evolves according to the SDE^1^

1.7 $$\begin{aligned} {\mathrm d}c_{\textrm{ap}} =&\big [ \tfrac{4}{3}c_{\textrm{ap}}\epsilon f(t)+\sigma ^2 g_Q(c_{\textrm{ap}}) \big ] \ {\mathrm d}t+\tfrac{2}{9}c_{\textrm{ap}}^{-1/2}\sigma \langle \phi _{c_{\textrm{ap}}}^2, {\mathrm d}W_t^Q \rangle , \end{aligned}$$

in which the function $g_Q:{\mathbb {R}}\rightarrow {\mathbb {R}}$ will be made explicit in Section 3. This allows us to demonstrate that stochastic stability is not limited to trivial changes in amplitude.

**Main result** We study solutions *u* to (1.1) in the (relatively standard) setting S1 – see Section 2 ahead – through the modulation Ansatz

$$\begin{aligned} u\big (t,x+\xi (t)\big )=\phi _{c(t)}(x)+v(t,x). \end{aligned}$$

Here, *c*(*t*) and ξ(t) are stochastic processes that track the soliton amplitude and position, respectively, which we fully define later on. In particular, we supply (1.1) with the initial condition

$$\begin{aligned} u(0,x)=\phi _{c_*}(x), \end{aligned}$$

for some $c_*>0$. The remainder *v*(*t*, *x*) constitutes the error resulting from our modulation approach. The main contribution of this work concerns the probabilistic behavior of the exit time

$$\begin{aligned} t_{\textrm{st}}(\eta )=\sup \big \{t\ge 0:\Vert v(t)\Vert _{H^1_w} \le \eta \big \}, \end{aligned}$$

which signals a deviation from the modulated soliton description.

The parameter *E* in our result below provides a-priori (but unlimited) control over the total potential impact of the deterministic forcing. This is related to the fact that the linear stability properties of the soliton family $\phi _c$ are limited by the soliton amplitude *c*. Indeed, the $w(c-w^2)$-wide spectral gap of the linear operator ${\mathcal {L}}_c$ on $L^2_w$ is at most $\frac{2}{3\sqrt{3}}c^{3/2}$ (take $w=\sqrt{c/3}$). The constants appearing in many of our estimates therefore require *c* to be kept within a fixed range $[c_{\textrm{min}}, c_{\textrm{max}}]$ that is controlled by *E*.

### Theorem 1.1

(See Section 8) Pick $c_*,E>0$ and $w\in (0,\sqrt{c_*}/3]$. Assuming S1, there exist constants $\eta _0,C,\delta >0$ such that the following holds true. For all $\eta \in [0,\eta _0]$, $C\sigma ,C\epsilon \in [0,\eta ]$, each T≥1, and each continuous function $f:{\mathbb {R}}^+\rightarrow {\mathbb {R}}$ for which

1.8 $$\begin{aligned} \sup _{t\ge 0}|f(t)|\le 1, \quad \text {and}\quad \epsilon \int _0^\infty |f(t)|{\mathrm d}t\le E, \end{aligned}$$

the exit time $t_{\textrm{st}}$ satisfies

1.9 $$\begin{aligned} \mathbb {P}\big [t_{\textrm{st}}(\eta )<T\big ]\le CT\sigma ^2\log (1/\sigma )+ CTe^{-\delta \eta ^2/\sigma ^2}. \end{aligned}$$

The probability bound (1.9) contains two contributions. The exponential part $Te^{-\delta \eta ^2/\sigma ^2}$ stems from the linear stability properties of the wave family (1.2) on weighted spaces, and matches modern phase-tracking results valid on time scales that are exponentially long with respect to the ratio $\eta ^2/ \sigma ^{2}$ [2, 3, 14, 15, 20]. The contribution $T\sigma ^2\log (1/\sigma )$, however, limits the time-scale on which stability can be obtained to times *T* that are *small* with respect to $\sigma ^{-2}\log (1/\sigma )^{-1}$, but independent of the size-parameter η.

The limiting factor is that we rely on a-priori control of the remainder *v* in the unweighted space $L^2$. In this space, the solitons $\phi _c$ are not linearly stable, and the norm $\Vert v(t)\Vert ^2_{L^2}$ grows linearly in time. The factor $\sigma ^2\log (1/\sigma )$ stems from an Itô drift term proportional to $\sigma ^2$, combined with a factor $\log (1/\sigma )$ that we need to account for the potentially large fluctuations of the soliton amplitude.

We emphasize that within the timescale discussed above, the deterministic forcing (ϵ>0) can cause significant modulation of the soliton amplitude *c*(*t*). Thus, the main point of our results is that they demonstrate that the stability of the wave family (1.2) is not limited to small fluctuations. To showcase this, we obtain a result on the exit time

$$\begin{aligned} t_{\textrm{ap}}(\lambda )=\sup \big \{t\ge 0:|c(t)-c_{\textrm{ap}}(t)|\le \lambda \big \}, \end{aligned}$$

which describes the validity of the approximation (1.7).

### Theorem 1.2

(See Section 9) Assuming the setting of Theorem 1.1, for each λ>0 that satisfies

$$\begin{aligned} \lambda \le \min \{\tfrac{1}{2}c_*e^{-3E},c_*e^{3E}\}, \end{aligned}$$

the exit times $t_{\textrm{ap}}$ and $t_{\textrm{st}}$ satisfy

1.10 $$\begin{aligned} \mathbb {P}\big [t_{\textrm{ap}}(\lambda )<T\big ]\le C\frac{\sigma ^2}{\lambda }\log (1/\sigma ). \end{aligned}$$

**Approach** The field of stochastic traveling waves has witnessed rapid development in recent years. Several approaches have emerged to study stochastic traveling waves in various PDE settings. These typically feature a decomposition of the solution in terms of a traveling wave modulated by a stochastic phase-shift. The exact definition of this phase-shift is where the various methods differ. Let us mention the phase-lag method [12, 18], variational-phase [2, 3, 15, 20] and isochronal-phase [1], all applied in the context of reaction-diffusion equations. In dispersive settings, (adaptations of) the variational phase have been applied in [9–11, 14, 28]. Let us also mention the recent work [29], which presents a phase-tracking mechanism for general symmetry groups.

In the present context, where we study the wave family (1.2), we follow our approach in [28] and decompose the solution *u* to (1.1) as

$$\begin{aligned} u\big (t,x+\xi (t)\big )=\phi _{c(t)}(x)+v(t,x). \end{aligned}$$

Here, *c*(*t*) and ξ(t) are processes that track the soliton amplitude and position, and ensure the orthogonality conditions

1.11 $$\begin{aligned} \big \langle v(t,\cdot ),\phi _{c(t)}\big \rangle =\big \langle v(t,\cdot ),\zeta _{c(t)}\big \rangle =0, \quad t\ge 0. \end{aligned}$$

We point out a technical but essential difference with [28], where we decomposed *u* in terms of a fixed soliton $\phi _{c_*}$ in a rescaled frame. We do not employ this coordinate-transformation here, to avoid Itô correction terms in the evolution equation of the remainder *v*. This leaves the challenge of harnessing the linear stability properties of a time-varying soliton $\phi _{c(t)}$. In spirit of [20], we employ the linear stability properties at some time T>0 of ${\mathcal {L}}_{c(T)}$ on an interval [T,T+ΔT]. On such intermediate intervals, the soliton amplitude *c*(*t*) does not deviate much from *c*(*T*). We furthermore introduce local modulation parameters on the interval [T,T+ΔT] resulting in a local remainder $v^T$ which enjoys exponential damping by the linear flow $\{e^{{\mathcal {L}}_{c(T)}t}\}_{t\ge 0}$. For $t\in [T,T+\Delta T]$, the local remainder can be used to control the global remainder *v*(*t*).

**Challenges** Classically, analysis of the $L^2_w$-norm of *v*(*t*) requires complementary control on the unweighted $L^2$-norm of *v*(*t*). This is due to the nonlinearity $u\partial _x u$ present in (1.1), which is typically estimated in the weighted $L^2$-norm as

1.12 $$\begin{aligned} \int _{{\mathbb {R}}} e^{2wx}u^2(x)u^2_x(x){\mathrm d}x\le \Vert u\Vert ^2_{\infty }\int _{{\mathbb {R}}} e^{2wx}u^2_x(x){\mathrm d}x\le 2\Vert u\Vert ^2_{H^1}\Vert u_x\Vert ^2_{L^2_w}. \end{aligned}$$

The unweighted $H^1$-norm of *v*(*t*) can in turn be analyzed via energy methods, see [28, Section 6]. A formal application of the Itô lemma shows that, in the presence of stochastic forcing (σ>0), the energy of solutions to (1.1) evolves as

$$\begin{aligned} {\mathrm d}\Vert u\Vert _{L^2}^2=\sigma ^2 \Vert u\Vert ^2_{L^2} \ {\mathrm d}t+2\epsilon f(t)\Vert u\Vert _{L^2}^2\ {\mathrm d}t+2 \sigma \langle u,u \ {\mathrm d}W_t^Q\rangle . \end{aligned}$$

As we will see later on, due to an Itô drift correction, the energy in *v*(*t*) grows proportionally to $\sigma ^2 t$ at leading order. We incur a further $\log ( 1/\sigma )$ penalty as a consequence of the ${\mathcal {O}}(v^2)$ terms, leading to the first term in (1.9).

At present, we can hence not carry out our arguments on a time-interval $[0,T_{\textrm{max}}\sigma ^{-2}]$ for *any* $T_{\textrm{max}}>0$, which we believe should be attainable with more refined arguments in the special case ϵ=0. Indeed, inspecting the approximation (1.7) with ϵ=0, we see that the effective soliton amplitude *c* is driven by the noise $\sigma W_t^Q$. We can hence expect *c* to be kept within the range $[c_{\textrm{min}}, c_{\textrm{max}}]$ on time scales proportional to $\sigma ^{-2}$ and the (arbitrary) size of this range.

Furthermore, the bound (1.12) shows that it is in fact sufficient to control the *supremum* norm of *v*(*t*), instead of an energy norm. Preliminary numerical investigations suggest that this norm remains under control for timescales longer than $\sigma ^{-2}$, showing that the dynamics of *c* are indeed dominant. However, bounds of this nature will require us to depart from energy-based methods, which are only available in $L^2$-based spaces. We envision that future work in this direction will center on a careful (and challenging) direct pointwise analysis of the dispersive dynamics that drive the remainder *v*(*t*) in the wake of the soliton. We emphasize however that the framework developed here to control the weighted norm of *v* can readily accommodate such a refinement.

**Outline** This paper is organized as follows. In Section 2, we outline the setting of (1.1) in more detail and recall classical linear stability results regarding the operator ${\mathcal {L}}_c$ defined in (1.3). Then, in Section 3, we analyze the modulation system brought forth by the conditions (1.11). Following this, in Section 4, we introduce local approximations to this system. In Section 5, we carry out time-discretized stability arguments for the local remainder $v^T$ in the weighted spaces $H^1_w$. We supplement this with an analysis of the local modulation parameters in Section 6. In Section 7, we develop global control of the unweighted $L^2$-norm of the remainder *v*, as well as the global amplitude parameter *c*(*t*). Finally, we combine our results in Section 8 and Section 9 for the proofs of Theorem 1.1 and Theorem 1.2.

## Preliminaries

Below, we describe the setting of (1.1) in more detail and collect several results regarding the linear stability of the soliton family (1.2) as developed in [25].

**Stochastic set-up** We work in the following setting, which we recall from [27, Section 2.1.2].

**S1**: * We have *$c_*>0$, *and supply *(1.1) *with the initial condition*
  2.1 $$\begin{aligned} u(0,x)=\phi _{c_*}(x).\end{aligned}$$
  *The operator *$Q\in {\mathcal {L}}(L^2)$* is defined as the convolution with an even function *$q\in H^1\cap L^1$* through *
  $$\begin{aligned}Qf(x)=\int _{{\mathbb {R}}}q(x-y)f(y)\textrm{d} y.\end{aligned}$$
  *Moreover, the Fourier transform *${\hat{q}}$ *of the kernel q is non-negative. Lastly, the forcing term *$f:{\mathbb {R}}^+\rightarrow {\mathbb {R}}$* is continuous and bounded as*
  $$\begin{aligned}|f(t)|\le 1, \quad t\ge 0.\end{aligned}$$

By Young’s convolution inequality, the integrability assumption $q\in L^1$ ensures that *Q* is a bounded operator on $L^2({\mathbb {R}})$. The non-negativity of the Fourier transform ${\hat{q}}$ furthermore ensures that *Q* is a symmetric and non-negative operator. Thus, *Q* can be used to construct an $L^2$-valued cylindrical Brownian motion $W_t^Q$ on a filtered probability space $(\Omega ,{\mathcal {F}},\mathbb {F},\mathbb {P})$ cf. [7, 19], which satisfies the formal covariance identity

$$\begin{aligned} \mathbb {E}\bigl [{\mathrm d}W^Q(t,x){\mathrm d}W^Q(s,y)\bigr ]=\delta (t-s)q(x-y),\quad x,y\in {\mathbb {R}}, \quad t,s>0. \end{aligned}$$

Since *q* is an even function, the spatial correlation of $W^Q_t$ depends only on the distance |x-y| between two points $x,y\in {\mathbb {R}}$ and preserves the translation-invariance of (1.1). We recall furthermore from [27, Section 2.1.2] that the non-negative operator *Q* has a square-root $Q^{1/2}$ that acts as the convolution

$$\begin{aligned} Q^{1/2}f(x)=\int _{{\mathbb {R}}}q_{1/2}(x-y)f(y)\ {\mathrm d}y, \end{aligned}$$

where $q_{1/2}$ is the inverse Fourier transform of $\sqrt{{\hat{q}}}$.

Let us now introduce some notation related to stochastic integration with respect to the noise $W_t^Q$. Letting $\{e_k\}_{k=0}^{\infty }$ be an orthonormal basis of $L^2({\mathbb {R}})$, and introducing the space $L^2_Q:=Q^{1/2}(L^2)$ equipped with the inner product

$$\begin{aligned}\langle v, w\rangle _{L^2_Q}=\langle Q^{-1/2}v,Q^{-1/2}w\rangle _{L^2},\end{aligned}$$

we note that $L^2_Q$ is a separable Hilbert space for which $\{Q^{1/2}e_k\}_{k=0}^\infty$ is an orthonormal basis. We furthermore denote by $\textrm{HS}(L^2_Q,{\mathcal {H}})$ the space of Hilbert-Schmidt operators between $L^2_Q$ and a Hilbert space ${\mathcal {H}}$, equipped with the inner-product

$$\begin{aligned} \langle A,B\rangle _{\textrm{HS}(L^2_Q,{\mathcal {H}})}=\sum _{k=0}^\infty \bigl \langle A[Q^{1/2}e_k],B[Q^{1/2}e_k]\bigr \rangle _{{\mathcal {H}}}. \end{aligned}$$

In the setting S1, de Bouard and Debussche [8] showed that (1.1) admits unique mild solutions for purely stochastic forcing (ϵ=0). For deterministic forcing (σ=0), the well-posedness of (1.1) has been established in [28, lemma 2.1]. Combining these results yields the following well-posedness property, in which $\{e^{-\partial _x^3t}\}_{t\in {\mathbb {R}}}$ denotes the $C_0$-group of isometries on $L^2$ associated to the Airy equation.

### Lemma 2.1

([9, 28]) Assuming S1, (1.1) has a unique mild solution $u\in L^2(\Omega ;C({\mathbb {R}}^+;L^2({\mathbb {R}}))$, that satisfies

$$\begin{aligned} u(t)&=e^{-\partial _x^3 t}u_0-\int _0^t e^{-\partial _x^3 (t-t')}\partial _x\big (u^2(t')\big ){\mathrm d}t'+\epsilon \int _0^t f(t')e^{-\partial _x^3 (t-t')}u(t'){\mathrm d}t'\\&\,\,+\sigma \int _0^t e^{-\partial _x^3 (t-t')}u(t'){\mathrm d}W_{t'}^Q, \end{aligned}$$

and has paths that are almost surely in $C({\mathbb {R}}^+;H^1({\mathbb {R}}))$.

**Linear stability tools**

Our arguments rely heavily on the linear stability properties of the operator ${\mathcal {L}}_c$ defined in (1.3), that were developed in [25]. We recall from this work that ${\mathcal {L}}_c$ satisfies the eigenvalue relations ${\mathcal {L}}_{c} \partial _x\phi _{c}=0$ and ${\mathcal {L}}_{c} \partial _c \phi _{c}=\partial _x\phi _{c}$, giving rise to a two-dimensional generalized kernel. The (formal) adjoint ${\mathcal {L}}^*_c$ also has a two-dimensional kernel, spanned by $\phi _c$ and the primitive

$$\begin{aligned}\zeta _c (x)=\int _{-\infty }^x \partial _c \phi _c(y){\mathrm d}y.\end{aligned}$$

This function satisfies $\zeta _c \in L^2_{-w}$ (but not $\zeta _c\in L^2$). With this notation in place, we note that the projection onto the generalized kernel of ${\mathcal {L}}_c$ is given by

2.2 $$\begin{aligned} P_{c} f=\langle f, \tfrac{2}{9}c^{-1/2}\zeta _c+\tfrac{2}{9}c^{-2}\phi _c \rangle \partial _x \phi _{c} +\langle f, \tfrac{2}{9}c^{-1/2}\phi _c\rangle \partial _c \phi _c. \end{aligned}$$

We write $Q_c=I-P_c$ for the complementary projection, and note that $\operatorname {Ker}(P_c)$ coincides with the subspace where the conditions (1.6) hold. We collect the following properties of the flow generated by ${\mathcal {L}}_c$, which demonstrate the parabolic nature of $-\partial _x^3$ on the weighted spaces $L^2_w$, after projecting out the neutral modes. First, let us fix limits $c_{\textrm{min}}, c_{\textrm{max}}$ and a weight *w* as follows.

**S2**: * The constants* $c_{\textrm{min}}, c_{\textrm{max}},w\in {\mathbb {R}}$ satisfy
  $$\begin{aligned}0<c_{\textrm{min}}<c_*< c_{\textrm{max}}\quad \text {and} \quad 0<w<\sqrt{c_{\textrm{min}}}/3.\end{aligned}$$

### Theorem 2.2

([23, 25]) Assume S2 and let $c\in [c_{\textrm{min}}, c_{\textrm{max}}]$. Then, ${\mathcal {L}}_{c}$ is the generator of a $C_0$-semigroup $\{e^{{\mathcal {L}}_ct}\}_{t\ge 0}$ on $H^s_w$ for any real *s*. For all b>0 which satisfy $b<w(c-w^2)$, there exists a constant M>0 such that, for all $g\in L_{w}^2$, t>0 and $k\in \{0,1\}$, we have

2.3 $$\begin{aligned} \Vert \partial _x^k e^{{\mathcal {L}}_{c}t}Q_c g\Vert _{L^2_{w}}\le Mt^{-k/2}e^{-b t}\Vert g\Vert _{L^2_{w}}, \end{aligned}$$

while for all $g\in L_{w}^1$ we have

2.4 $$\begin{aligned} \Vert \partial _x^k e^{{\mathcal {L}}_{c}t}Q_c g\Vert _{L^2_{w}}\le Mt^{-(2k+1)/4}e^{-b t}\Vert g\Vert _{L^1_{w}}. \end{aligned}$$

## Global modulation system

With these preliminaries in place, we are in shape to introduce the decomposition

3.1 $$\begin{aligned} u\big (t,x+\xi (t)\big )=\phi _{c(t)}(x)+v(t,x) \end{aligned}$$

characterized by the orthogonality conditions

3.2 $$\begin{aligned} \big \langle v(t,\cdot ),\phi _{c(t)}\big \rangle =\big \langle v(t,\cdot ),\zeta _{c(t)}\big \rangle =0. \end{aligned}$$

As a consequence of Lemma 2.1, the remainder *v* introduced through (3.1) has paths that are almost surely in $C({\mathbb {R}}^+;H^1({\mathbb {R}}))$. The decomposition (3.1) is equivalent to that in [27, 28], albeit phrased in a different frame of reference. The unique existence of decomposition (3.1) is guaranteed as long as $\Vert v(t)\Vert _{L^2_w}$ remains sufficiently small, essentially as a result of the implicit function theorem.

### Lemma 3.1

([23]) Assuming S2, there exist constants $\delta _1, C_1>0$ such for each $v_* \in H^1_w\cap H^1$ with $\Vert v_*\Vert _{L^2_w}\le \delta _1$ and $c_*\in [c_{\textrm{min}},c_{\textrm{max}}]$, there exist unique parameters $c>0, \xi \in {\mathbb {R}}$ and a unique function $v\in H^1_w\cap H^1$ that together satisfy the identities

$$\begin{aligned}\phi _{c_*}(x)+v_*(x)=\phi _{c}(x-\xi )+v(x-\xi ) \quad \text {with} \quad \langle v,\phi _{c}\rangle =\langle v,\zeta _{c}\rangle =0\end{aligned}$$

and the bounds

$$\begin{aligned}\Vert v\Vert _{H^1_w}+|\xi |+|c_*-c|\le&C_1\Vert v_*\Vert _{H^1_w},\\ \Vert v\Vert _{H^1}\le&C_1\big (\Vert v_*\Vert _{H^1}+\Vert v_*\Vert _{H^1_w}\big ). \end{aligned}$$

For convenience, we introduce a phase-shift function Ω(t) through $\xi (t)=\int _0^t c(t') {\mathrm d}t'+\Omega (t)$. Our goal in this section is to describe the evolution of the modulation parameters *c*(*t*) and Ω(t) via SDEs of the form

3.3 $$\begin{aligned} {\mathrm d}c =&c_d^{\sigma ,\epsilon }(v,c,t) \ {\mathrm d}t+\sigma \big \langle c_s(v,c), T_{\xi } {\mathrm d}W_t^Q \big \rangle , \end{aligned}$$

3.4 $$\begin{aligned} {\mathrm d}\Omega =&\Omega _d^{\sigma ,\epsilon }(v,c,t)\ {\mathrm d}t+ \sigma \big \langle \Omega _s(v,c), T_{\xi } {\mathrm d}W_t^Q \big \rangle . \end{aligned}$$

For $\xi \in {\mathbb {R}}$, the operator $T_{\xi }$ above denotes the translation by ξ, i.e. $(T_{\xi } f)(x)=f(x+\xi )$. We thus set out to find mappings $c_d^{\sigma ,\epsilon }, c_s, \Omega _d^{\sigma ,\epsilon }$ and $\Omega _s$ that ensure the conditions (3.2). Formally applying the Itô lemma [7, Theorem 4.32] to (3.1) with (3.3) and (3.4), yields, after tedious computations

3.5 $$\begin{aligned} {\mathrm d}v&=\big [{\mathcal {L}}_{c(t)}v+Y^{\sigma ,\epsilon }(v,c,t)\big ]\ {\mathrm d}t+ \sigma Z(v,c)\ T_{\xi } {\mathrm d}W_t^Q \end{aligned}$$

where

$$\begin{aligned} Y^{\sigma ,\epsilon }(v,c,t)&=N(v)+\epsilon f(t)(\phi _c+v)+ \Omega _d^{\sigma ,\epsilon }(v,c,t)\partial _x(\phi _c+v)\\&\quad -c_d^{\sigma ,\epsilon }(v,c,t)\partial _c\phi _c+\sigma ^2 Y_d(v,c). \end{aligned}$$

Here, $N(v)=-\partial _x(v^2)$ is the KdV nonlinearity, the drift contribution $Y_d$ is given by

$$\begin{aligned} Y_d(v,c)&= \tfrac{1}{2} \big \Vert Q^{1/2}\Omega _s(v,c)\big \Vert _{L^2}^2 \partial _x^2v+ \tfrac{1}{2} \big \Vert Q^{1/2}\Omega _s(v,c)\big \Vert _{L^2}^2 \partial _x^2\phi _c\\&\quad + \tfrac{1}{2}\big \Vert Q^{1/2}c_s(v,c)\big \Vert _{L^2}^2 \partial _c^2\phi _{c}\end{aligned}$$

and the stochastic component is defined by

$$\begin{aligned} Z(v,c)[h]=&\Big (h +\big \langle \Omega _s(v,c),h\big \rangle \partial _x \Big )v +\Big (h+ \big \langle \Omega _s(v,c),h\big \rangle \partial _x - \big \langle c_s(v,c),h \big \rangle \partial _c \Big )\phi _{c}. \end{aligned}$$

The formal adjoint of *Z* is given by

$$\begin{aligned} Z^*(v,c)[g]=&(g+\phi _c)v +\Omega _s(v,c)\big \langle g,\partial _x (\phi _c+v)\big \rangle - c_s(v,c) \langle g, \partial _c\phi _{c}\rangle . \end{aligned}$$

The evolution of $\big \langle v(t),\phi _{c(t)}\big \rangle$ can now formally be obtained by applying the Itô product rule. However, we can not expect (3.5) to hold in the strong sense, as *v* is not regular enough for ${\mathcal {L}}_{c(t)}v$ and $\partial _x^2 v$ to be well-defined. We take care of this technical issue in Appendix B by resorting to a mild Itô formula. The result is as follows.

### Lemma 3.2

(See Appendix B) Assume S1 and S2. For each t≥0, the inequalities

$$\begin{aligned}\Vert v(t')\Vert _{L^2_w}\le \delta _1 \quad \text {and} \quad c(t')\in [c_{\textrm{min}},c_{\textrm{max}}],\quad t' \in [0,t]\end{aligned}$$

imply that

3.6 $$\begin{aligned}&{\mathrm d}\big \langle v(t),\phi _{c(t)}\big \rangle =\Big ( c_d^{\sigma ,\epsilon }(v,c,t) \langle v,\partial _c \phi _{c(t)}\rangle +\big \langle Y^{\sigma ,\epsilon }(v,c,t),\phi _{c(t)}\big \rangle \Big )\ {\mathrm d}t\nonumber \\&+\sigma ^2\Big (\tfrac{1}{2}\big \Vert Q^{1/2}c_s(v,c)\big \Vert _{L^2}^2 \langle v,\partial _c^2 \phi _{c(t)}\rangle + \big \langle Q^{1/2}Z^*(v,c)[\partial _c\phi _{c(t)}],Q^{1/2}c_s(v,c) \big \rangle \Big )\ {\mathrm d}t \nonumber \\&+\sigma \langle v,\partial _c\phi _{c(t)}\rangle \big \langle c_s(v,c),T_{\xi }{\mathrm d}W_t^Q\big \rangle +\sigma \big \langle Z(v,c)[T_{\xi }{\mathrm d}W_t^Q],\phi _{c(t)}\big \rangle \end{aligned}$$

and

3.7 $$\begin{aligned}&{\mathrm d}\big \langle v(t),\zeta _{c(t)}\big \rangle =\Big ( c_d^{\sigma ,\epsilon }(v,c,t) \langle v,\partial _c \zeta _{c(t)}\rangle +\big \langle Y^{\sigma ,\epsilon }(v,c,t),\zeta _{c(t)}\big \rangle \Big )\ {\mathrm d}t\nonumber \\&+\sigma ^2\Big (\tfrac{1}{2}\big \Vert Q^{1/2}c_s(v,c)\big \Vert _{L^2}^2 \langle v,\partial _c^2 \zeta _{c(t)}\rangle + \big \langle Q^{1/2}Z^*(v,c)[\partial _c\zeta _{c(t)}],Q^{1/2}c_s(v,c) \big \rangle \Big )\ {\mathrm d}t \nonumber \\&+\sigma \langle v,\partial _c\zeta _{c(t)}\rangle \big \langle c_s(v,c),T_{\xi }{\mathrm d}W_t^Q\big \rangle +\sigma \big \langle Z(v,c)[T_{\xi }{\mathrm d}W_t^Q],\zeta _{c(t)}\big \rangle \end{aligned}$$

hold $\mathbb {P}$-almost surely.

### Remark

In (3.6) and (3.7) above, derivatives on *v* are interpreted in the weak sense.

Solving for solutions $c_s(v,c)$ and $\Omega _s(v,c)$ to the system

$$\begin{aligned} \begin{bmatrix} \langle v,\partial _c\phi _c\rangle \big \langle c_s(v,c),h \big \rangle +\big \langle Z(v,c)[h],\phi _c\big \rangle \\ \langle v,\partial _c\zeta _c\rangle \big \langle c_s(v,c),h \big \rangle +\big \langle Z(v,c)[h],\zeta _c\big \rangle \end{bmatrix}=\begin{bmatrix} 0\\ 0 \end{bmatrix}, \quad \forall h\in L^2_Q, \end{aligned}$$

reveals that

$$\begin{aligned} K_c(v)\begin{bmatrix} \langle c_s(v,c),h\rangle \\ \langle \Omega _s(v,c),h\rangle \end{bmatrix}=- \begin{bmatrix} \big \langle h(\phi _c+v),\phi _c\big \rangle \\ \big \langle h(\phi _c+v),\zeta _c\big \rangle \end{bmatrix} \end{aligned}$$

for all $h\in L^2_Q$, where $K_c(v)$ is the matrix

$$\begin{aligned} K_c(v)=\begin{bmatrix} \langle -\phi _c+v,\partial _c\phi _c\rangle &  \langle \partial _x v,\phi _c\rangle \\ \langle v,\partial _c\zeta _c\rangle -\langle \partial _c \phi _{c},\zeta _c\rangle &  \big \langle \partial _x (\phi _c+v),\zeta _c\big \rangle \end{bmatrix}. \end{aligned}$$

This implies that the mappings $c_s(v,c)$ and $\Omega _s(v,c)$ are given by

$$\begin{aligned}&\begin{bmatrix} c_s(v,c)\\ \Omega _s(v,c) \end{bmatrix}=-K_c^{-1}(v)\begin{bmatrix} (\phi _c+v)\phi _c \\ (\phi _c+v)\zeta _c \end{bmatrix}. \end{aligned}$$

We solve for $c_d^{\sigma ,\epsilon }$ and $\Omega _d^{\sigma ,\epsilon }$ by decomposing

$$\begin{aligned} c_d^{\sigma ,\epsilon }(v,c,t)=&c^0_d(v,c)+\epsilon f(t)c_f(v,c)+\sigma ^2 c_d(v,c), \\ \Omega _d^{\sigma ,\epsilon }(v,c,t)=&\Omega ^0_d(v,c)+\epsilon f(t)\Omega _f(v,c)+\sigma ^2 \Omega _d(v,c), \end{aligned}$$

and isolating the $\sigma ^2$ and ϵ dependent terms in (3.6) and (3.7). This yields

$$\begin{aligned} \begin{bmatrix} c_d^0(v,c)\\ \Omega _d^0(v,c) \end{bmatrix}=&-K_c^{-1}(v)\begin{bmatrix} \big \langle N(v),\phi _c\big \rangle \\ \big \langle N(v),\zeta _c \big \rangle \end{bmatrix},\\ \begin{bmatrix} c_f(v,c)\\ \Omega _f(v,c) \end{bmatrix}=&-K_c^{-1}(v)\begin{bmatrix} \langle \phi _c+v,\phi _c\rangle \\ \langle \phi _c+v,\zeta _c \rangle \end{bmatrix}, \end{aligned}$$

together with

$$\begin{aligned} \begin{bmatrix} c_d(v,c)\\ \Omega _d(v,c) \end{bmatrix}=&-K_c^{-1}(v)\begin{bmatrix} \big \langle Y_d(v,c),\phi _c\big \rangle \\ \big \langle Y_d(v,c),\zeta _c \big \rangle \end{bmatrix}-\tfrac{1}{2}\big \Vert Q^{1/2}c_s(v,c)\big \Vert _{L^2}^2K_c^{-1}(v)\begin{bmatrix} \langle v,\partial _c^2 \phi _c\rangle \\ \langle v,\partial _c^2 \zeta _c\rangle \end{bmatrix}\\&-K_c^{-1}(v)\begin{bmatrix} \big \langle Q^{1/2}Z^*(v,c)[\partial _c\phi _c],Q^{1/2}c_s(v,c) \big \rangle \\ \big \langle Q^{1/2}Z^*(v,c)[\partial _c\zeta _c],Q^{1/2}c_s(v,c) \big \rangle \end{bmatrix}. \end{aligned}$$

We remark that, in the absence of deterministic forcing (ϵ=0), the modulation system derived above is equivalent to the system found in [27, Section 2.3.2], and in the absence of stochastic forcing (σ=0) to that in [28, Section 2]. Evaluating the modulation system at v=0 gives rise to the approximation $c_{\textrm{ap}}$ defined in (1.7), with $g_Q(c)=c_d(0,c)$. Now that the modulation system has taken concrete form, we can establish control on the modulation parameters, which will be important for our stability arguments later on.

### Lemma 3.3

Assuming S2, there exist constants $\delta _2, C_2>0$ such that for all ${\tilde{v}}\in L^2_w$ that satisfy $\Vert {\tilde{v}}\Vert _{L^2_w}\le \delta _2$ and each ${\tilde{c}}\in [c_{\textrm{min}},c_{\textrm{max}}]$ we have

3.8 $$\begin{aligned}&\big \Vert Q^{1/2}c_s({\tilde{v}},{\tilde{c}})-Q^{1/2}c_s(0,{\tilde{c}})\big \Vert _{L^2}+\big \Vert Q^{1/2}\Omega _s({\tilde{v}},{\tilde{c}})-Q^{1/2}\Omega _s(0,{\tilde{c}})\big \Vert _{L^2}\le C_2\Vert {\tilde{v}}\Vert _{L^2_w},\end{aligned}$$

3.9 $$\begin{aligned}&\quad \big |c_d^0({\tilde{v}},{\tilde{c}})\big |+\big |\Omega _d^0({\tilde{v}},{\tilde{c}})\big |\le C_2 \Vert {\tilde{v}}\Vert _{L^2_w}^2,\end{aligned}$$

3.10 $$\begin{aligned}&\quad \big |c_d({\tilde{v}},{\tilde{c}})-{\tilde{c}}_d(0,{\tilde{c}})\big |+\big |\Omega _d({\tilde{v}},{\tilde{c}})-\Omega _d(0,{\tilde{c}})\big |\le C_2\big (1+\Vert {\tilde{v}}\Vert _{L^2_w}^2\big )\Vert {\tilde{v}}\Vert _{L^2_w},\end{aligned}$$

3.11 $$\begin{aligned}&\quad \big |c_f({\tilde{v}},{\tilde{c}})-c_f(0,{\tilde{c}})\big |+\big |\Omega _f({\tilde{v}},{\tilde{c}})-\Omega _f(0,{\tilde{c}})\big |\le C_2\Vert {\tilde{v}}\Vert _{L^2_w}. \end{aligned}$$

### Proof

Setting out to control $K_{{\tilde{c}}}^{-1}({\tilde{v}})$, we note that

$$\begin{aligned}K_{{\tilde{c}}}(0)=-\frac{9}{2}\begin{bmatrix} {{\tilde{c}}}^{1/2}&  0\\ {{\tilde{c}}}^{-1} & {{\tilde{c}}}^{1/2} \end{bmatrix}\end{aligned}$$

is invertible, so that we can find constants ${\tilde{C}}_1,{\tilde{C}}_2>0$ that ensure $\Vert A^{-1}\Vert _{\textrm{op}}\le {\tilde{C}}_2$ for all $A\in {\mathbb {R}}^{2\times 2}$ which satisfy

$$\begin{aligned}\big |A_{ij}-[K_{{{\tilde{c}}}}(0)]_{ij}\big |\le {\tilde{C}}_1, \quad (i,j) \in \{1,2\}^2.\end{aligned}$$

Here, $\Vert \cdot \Vert _{\textrm{op}}$ denotes the operator-norm on $\big ({\mathbb {R}}^2, \Vert \cdot \Vert _1\big )$, chosen for convenience in the computations below. Now note that

$$\begin{aligned} \big |[K_{{{\tilde{c}}}}({\tilde{v}})]_{ij}-[K_{{{\tilde{c}}}}(0)]_{ij}\big |\le&\Big (\big |\langle {\tilde{v}},\partial _c\phi _{{\tilde{c}}}\rangle \big |+\big |\langle {\tilde{v}},\partial _x\phi _{{\tilde{c}}}\rangle \big |+\big |\langle {\tilde{v}},\partial _c\zeta _{{\tilde{c}}}\rangle \big |\Big )\\ \le&\big (\Vert \partial _c\phi _{{\tilde{c}}}\Vert _{L^2_{-w}}+\Vert \partial _x\phi _{{\tilde{c}}}\Vert _{L^2_{-w}}+\Vert \partial _c\zeta _{{\tilde{c}}}\Vert _{L^2_{-w}}\big )\Vert {\tilde{v}}\Vert _{L^2_w} \end{aligned}$$

for all $(i,j)\in \{1,2\}^2$. Ensuring $\delta _2\le \big (\Vert \partial _c\phi _{{\tilde{c}}}\Vert _{L^2_{-w}}+\Vert \partial _x\phi _{{\tilde{c}}}\Vert _{L^2_{-w}}+\Vert \partial _c\zeta _{{\tilde{c}}}\Vert _{L^2_{-w}}\big )^{-1}{\tilde{C}}_1$, it follows that $\big \Vert K_{{\tilde{c}}}^{-1}({\tilde{v}})\big \Vert _{\textrm{op}}\le {\tilde{C}}_2$. Estimating

$$\begin{aligned} \Big |\big \langle N({\tilde{v}}),\phi _{{{\tilde{c}}}}\big \rangle \Big |+\Big |\big \langle N({\tilde{v}}),\zeta _{{{\tilde{c}}}}\big \rangle \Big |=&\Big |\big \langle \partial _x({\tilde{v}}^2),\phi _{{{\tilde{c}}}}\big \rangle \Big |+\Big |\big \langle \partial _x({\tilde{v}}^2),\zeta _{{{\tilde{c}}}}\big \rangle \Big |\\ =&\big |\langle {\tilde{v}}^2,\partial _x\phi _{{{\tilde{c}}}}\rangle \big |+\big |\langle {\tilde{v}}^2,\partial _c\phi _{{{\tilde{c}}}}\rangle \big |\\ \le&{\tilde{C}}_5\Vert {\tilde{v}}\Vert ^2_{L^2_{w}} \end{aligned}$$

where

$$\begin{aligned} {\tilde{C}}_5= \sup _{x\le 0}\Big (|e^{-2w x}\partial _x\phi _{{{\tilde{c}}}}(x)|+|e^{-2w x}\partial _c\phi _{{{\tilde{c}}}}(x)|\Big ) \end{aligned}$$

establishes (3.9). Writing

$$\begin{aligned} \begin{bmatrix} c_f({\tilde{v}},{{\tilde{c}}})-c_f(0,{\tilde{c}})\\ \Omega _f({\tilde{v}},{{\tilde{c}}})-\Omega _f(0,{\tilde{c}}) \end{bmatrix}=-K_{{\tilde{c}}}^{-1}({\tilde{v}})\begin{bmatrix} \langle {\tilde{v}},\phi _{{\tilde{c}}}\rangle \\ \langle {\tilde{v}},\zeta _{{\tilde{c}}} \rangle \end{bmatrix}+\big (K_{{\tilde{c}}}(0)^{-1}-K_{{\tilde{c}}}^{-1}({\tilde{v}})\big )\begin{bmatrix} \langle \phi _{{\tilde{c}}},\phi _{{\tilde{c}}}\rangle \\ \langle \phi _{{\tilde{c}}},\zeta _{{\tilde{c}}} \rangle \end{bmatrix} \end{aligned}$$

establishes (3.11). Writing

$$\begin{aligned}&\begin{bmatrix} Q^{1/2}c_s({\tilde{v}},{{\tilde{c}}})-Q^{1/2}c_s(0,{{\tilde{c}}})\\ Q^{1/2}\Omega _s({\tilde{v}},{{\tilde{c}}})-Q^{1/2}\Omega _s(0,{{\tilde{c}}}) \end{bmatrix} =-K_{{\tilde{c}}}^{-1}({\tilde{v}})\begin{bmatrix} Q^{1/2}({\tilde{v}}\phi _{{\tilde{c}}}) \\ Q^{1/2}({\tilde{v}}\zeta _{{\tilde{c}}}) \end{bmatrix}\\&\quad +\big (K_{{\tilde{c}}}^{-1}(0)-K_{{\tilde{c}}}^{-1}({\tilde{v}})\big )\begin{bmatrix} Q^{1/2}(\phi ^2_{{\tilde{c}}}) \\ Q^{1/2}(\phi _{{\tilde{c}}}\zeta _{{\tilde{c}}}) \end{bmatrix} \end{aligned}$$

together with

$$\begin{aligned}\Vert Q^{1/2}g\Vert _{L^2}=\Vert \sqrt{{\hat{q}}}{\hat{g}}\Vert _{L^2}\le \Vert {\hat{q}}\Vert ^{1/2}_\infty \Vert {\hat{g}}\Vert _{L^2}\le \Vert q\Vert ^{1/2}_{L^1}\Vert g\Vert _{L^2}, \quad g\in L^2\end{aligned}$$

and

$$\begin{aligned}\Vert {\tilde{v}}\phi _{{\tilde{c}}}\Vert _{L^2}+\Vert {\tilde{v}}\zeta _{{\tilde{c}}}\Vert _{L^2}\le \big (\Vert \phi _{{\tilde{c}}}\Vert _{L^2_{-w}}+\Vert \zeta _{{\tilde{c}}}\Vert _{L^2_{-w}}\big )\Vert {\tilde{v}}\Vert _{L^2_w}\end{aligned}$$

establishes (3.8). Lastly, (3.10) follows in the same way, using

$$\begin{aligned}&\Big |\big \langle Y_d({\tilde{v}},{{\tilde{c}}})-Y_d(0,{{\tilde{c}}}),\phi _{{\tilde{c}}}\big \rangle \Big |+\Big |\big \langle Y_d({\tilde{v}},{{\tilde{c}}})-Y_d(0,{{\tilde{c}}}),\zeta _{{\tilde{c}}}\big \rangle \Big |\\&\quad \le \tfrac{1}{2}\Big | \big \Vert Q^{1/2}\Omega _s({\tilde{v}},{{\tilde{c}}})\big \Vert _{L^2}^2-\big \Vert Q^{1/2}\Omega _s(0,{{\tilde{c}}})\big \Vert _{L^2}^2\Big | \Big ( \big |\langle \partial _x^2\phi _{{\tilde{c}}},\phi _{{\tilde{c}}}\rangle \big |+\big |\langle \partial _x^2\phi _{{\tilde{c}}}, \zeta _{{\tilde{c}}}\rangle \big |\Big )\\&\quad +\tfrac{1}{2}\Big | \big \Vert Q^{1/2}c_s({\tilde{v}},{{\tilde{c}}})\big \Vert _{L^2}^2-\big \Vert Q^{1/2}c_s(0,{{\tilde{c}}})\big \Vert _{L^2}^2\Big | \Big ( \big |\langle \partial _c^2\phi _{{\tilde{c}}},\phi _{{\tilde{c}}}\rangle \big |+\big |\langle \partial _c^2\phi _{{\tilde{c}}}, \zeta _{{\tilde{c}}}\rangle \big |\Big )\\&\quad +\tfrac{1}{2} \big \Vert Q^{1/2}\Omega _s({\tilde{v}},{{\tilde{c}}})\big \Vert _{L^2}^2\Big (\big |\langle {\tilde{v}},\partial _x^2\phi _{{\tilde{c}}}\rangle \big |+\big |\langle {\tilde{v}},\partial _x^2\zeta _{{\tilde{c}}}\rangle \big |\Big )\\ \le&{\tilde{C}}_6 \big (1+\Vert {\tilde{v}}\Vert _{L^2_w}+\Vert {\tilde{v}}\Vert _{L^2_w}^2\big )\Vert {\tilde{v}}\Vert _{L^2_w} \end{aligned}$$

and

$$\begin{aligned} \Big \Vert \big (Z^*({\tilde{v}},{{\tilde{c}}})-Z^*(0,{{\tilde{c}}})\big )[g]\Big \Vert _{L^2}\le&\big \Vert (g+\phi _{{\tilde{c}}}){\tilde{v}}\big \Vert _{L^2} +\big \Vert \Omega _s({\tilde{v}},{{\tilde{c}}})-\Omega _s(0,{{\tilde{c}}})\big \Vert _{L^2}\big |\langle \partial _x g, \phi _{{\tilde{c}}}+{\tilde{v}}\rangle \big |\\&+\big \Vert \Omega _s({\tilde{v}},{{\tilde{c}}})\big \Vert _{L^2}\big |\langle \partial _x g, {\tilde{v}}\rangle \big |+ \big \Vert c_s({\tilde{v}},{{\tilde{c}}})-c_s(0,{{\tilde{c}}})\big \Vert _{L^2} \big |\langle g, \partial _c\phi _{{{\tilde{c}}}}\rangle \big |\\ \le&{\tilde{C}}_7\big (1+\Vert {\tilde{v}}\Vert _{L^2_w}\big )\Vert {\tilde{v}}\Vert _{L^2_w}.\end{aligned}$$

□

## Local modulation system

Now that we have set up the modulation system $\big (v(t),c(t),\xi (t)\big )$, we turn our attention to the main goal of this paper: asserting that the remainder *v*(*t*) defined through

$$\begin{aligned}v(t,x)=u\big (t,x+\xi (t)\big )-\phi _{c(t)}(x)\end{aligned}$$

remains small (measured in the norm $$H^1_w)$$. However, we do not base our arguments on the operator $${\mathcal {L}}_{c(t)}$$ that represents the linear part of (3.5). The main reason is that $${\mathcal {L}}_{c(t)}$$ is non-autonomous, which complicates stability arguments based on the stability properties of the flow generated by $${\mathcal {L}}_{c}$$. A second reason is that *v* is defined through a stochastic shift of *u*, and hence (3.5) contains the term $$\partial _x^2v$$, which presents regularity issues.

Given T≥0, we therefore introduce a *local* modulation system ${\textbf{m}}^T:=\big (v^T,c^T,\xi ^T\big )$ through

4.1 $$\begin{aligned} v^T(s,x)=&u\big (T+s,x+\xi (T)+c(T)s\big )-\Phi ^T({\textbf{m}}^T(s),s,x), \end{aligned}$$

where we have abbreviated

$$\begin{aligned} \Phi ^T({\textbf{m}}^T(s),s,x):=\phi _{c^T(s)}\big (x+\xi (T)+c(T)s-\xi ^T(s)\big ). \end{aligned}$$

The local remainder $v^T(s)$ is defined by shifting *u* with constant velocity *c*(*T*). This freezes the wave around the origin in the absence of forcing, while avoiding an Itô correction term of the form $\partial _x^2$. The parameter $\xi ^T(s)$ then has the interpretation as soliton position, and accounts for corrections due to the forcing. The local modulation parameters $c^T(s)$ and $\xi ^T(s)$ are uniquely determined through the condition

4.2 $$\begin{aligned} \big \langle v^T(s),\phi _{c(T)}\big \rangle =\big \langle v^T(s),\zeta _{c(T)}\big \rangle =0. \end{aligned}$$

In particular, we have

$$\begin{aligned} \big (v^T(0),c^T(0),\xi ^T(0)\big )=\big (v(T),c(T),\xi (T)\big ). \end{aligned}$$

In the absence of noise and forcing, with v(T)=0, the local modulation parameters keep their constant value $c^T(s)=c(T)$ and $\xi ^T(s)=\xi (T)$. The unique existence of the decomposition (4.1) is guaranteed by the following lemma, a variation on Lemma 3.1 tailored to the condition (4.2).

### Lemma 4.1

Assuming S2, there exists a constant $\delta _3>0$ so that for each $v_* \in H^1_w\cap H^1$ and $c_*,c_0\in [c_{\textrm{min}},c_{\textrm{max}}]$ with $\Vert v_*\Vert _{L^2_w},|c_*-c_0|\le \delta _3$, there exist unique parameters $c>0, \xi \in {\mathbb {R}}$ and a unique function $v\in H^1_w\cap H^1$ that together enforce the identities

$$\begin{aligned} \phi _{c_*}(x)+v_*(x)=\phi _{c}(x-\xi )+v(x-\xi ) \quad \text {with} \quad \langle v,\phi _{c_0}\rangle =\langle v,\zeta _{c_0}\rangle =0. \end{aligned}$$

Existence of the local decomposition is thus guaranteed as long as the remainder $v^T(s)$ and the difference $|c(T)-c^T(s)|$ remain under $\delta _3$. The advantage of defining the local system through the conditions (4.2) is that it demands orthogonality with respect to *fixed* eigenfunctions $\phi _{c(T)}$ and $\zeta _{c(T)}$, facilitating the use of the stability properties of $\{e^{{\mathcal {L}}_{c(T)}t}\}_{t\ge 0}$ to control the growth of $v^T(s)$. Rewriting (4.1) as

$$\begin{aligned} v^T(s,x) =&v\big (T+s,x+\xi (T)+c(T)s-\xi (T+s)\big )\\&+\phi _{c(T+s)}\big (x+\xi (T)+c(T)s-\xi (T+s)\big )-\Phi ^T({\textbf{m}}^T(s),s,x), \end{aligned}$$

we observe that as long as the local modulation parameters $c^T(s)$ and $\xi ^T(s)$ do not deviate substantially from their global counterparts c(T+s) and ξ(T+s), then neither do $v^T(s)$ and v(T+s). As long as this holds, we can understand the growth of v(T+s) through $v^T(s)$. The following lemma asserts this correspondence.

### Lemma 4.2

Assuming S1 and S2, there exists a constant $C_4>0$ such that the following holds true. For all $T,s,\delta \ge 0$, the inclusions

$$\begin{aligned} c^T(s),c(T+s)\in [\tfrac{1}{2}c_{\textrm{min}}, 2c_{\textrm{max}}] \end{aligned}$$

and the bound

$$\begin{aligned} \big |c^T(s)-c(T+s)\big |+\big |\xi ^T(s)-\xi (T+s)\big |\le \delta , \end{aligned}$$

imply

$$\begin{aligned} \Big |\big \Vert v(T+s)\big \Vert _{H^1_w}-e^{w(\xi (T)+c(T)s-\xi (T+s))}\big \Vert v^T(s)\big \Vert _{H^1_w}\Big |\le C_4\delta . \end{aligned}$$

### Proof

We compute

$$\begin{aligned}&\Big |\big \Vert e^{w\cdot }v(T+s,\cdot )\big \Vert _{H^1}-e^{w(\xi (T)+c(T)s-\xi (T+s))}\big \Vert e^{w\cdot }v^T(s,\cdot )\big \Vert _{H^1}\Big |\\ =&\Big |\big \Vert e^{w\cdot }v(T+s,\cdot )\big \Vert _{H^1}-\big \Vert e^{w\cdot }v^T(s,\cdot -\xi (T)-c(T)s+\xi (T+s))\big \Vert _{H^1}\Big |. \end{aligned}$$

Using the reverse triangle inequality, we get

$$\begin{aligned}&\Big |\big \Vert e^{w\cdot }v(T+s,\cdot )\big \Vert _{H^1}-e^{w(\xi (T)+c(T)s-\xi (T+s))}\big \Vert e^{w\cdot }v^T(s,\cdot )\big \Vert _{H^1}\Big |\\ \le&\Big \Vert v(T+s)-v^T\big (s,\cdot -\xi (T)-c(T)s+\xi (T+s)\big )\Big \Vert _{H^1_w}\\ =&\Big \Vert \phi _{c(T+s)}-\phi _{c^T(s)}\big (\cdot +\xi (T+s)-\xi ^T(s)\big )\Big \Vert _{H^1_w}. \end{aligned}$$

The result now follows by exploiting the $O(e^{-\sqrt{c}|x|})$ decay of the wave-profile

$$\begin{aligned} \phi _c(x)=\tfrac{3c}{2}{{\,\textrm{sech}\,}}^2(\sqrt{c}x/2)=\frac{6c}{(e^{-\sqrt{c}x/2}+e^{\sqrt{c}x/2})^2} \end{aligned}$$

and its derivatives $\partial _x\phi _c, \partial _x^2\phi _c, \partial _c\phi _c$ and $\partial ^2_{cx}\phi _c$. Indeed, it implies that the map $c \mapsto e^{wx}\phi _c(x)+e^{wx}\partial _x\phi _c(x)$ is Lipschitz from $[0,2c_{\textrm{max}}]$ to $L^2$. □

Let us proceed by describing the dynamics of the local modulation system. We once again introduce a phase-shift parameter $\Omega ^T(s)$ through

$$\begin{aligned}\xi ^T(s)=\int _0^s c^T(s'){\mathrm d}s'+\Omega ^T(s)\end{aligned}$$

and will see that $c^T$ and $\Omega ^T$ satisfy SDEs of the form

4.3 $$\begin{aligned} {\mathrm d}c^{T} =&c_d^{\sigma ,\epsilon ,T}({\textbf{m}}^T(s),s) \ {\mathrm d}s+\sigma \big \langle c^{T}_s({\textbf{m}}^T(s),s),T_{\xi (T)+c(T)s}{\mathrm d}W_{T+s}^Q\big \rangle , \end{aligned}$$

4.4 $$\begin{aligned} {\mathrm d}{\Omega }^{T} =&\Omega _d^{\sigma ,\epsilon ,T}({\textbf{m}}^T(s),s) \ {\mathrm d}s+\sigma \big \langle \Omega ^{T}_s({\textbf{m}}^T(s),s),T_{\xi (T)+c(T)s}{\mathrm d}W_{T+s}^Q\big \rangle . \end{aligned}$$

A formal application of Itô’s lemma then shows that

4.5 $$\begin{aligned} {\mathrm d}v^T={\mathcal {L}}_{c(T)}v^T\ {\mathrm d}s+Y^{\sigma ,\epsilon ,T}({\textbf{m}}^T(s),s)\ {\mathrm d}s+ \sigma Z^{T}({\textbf{m}}^T(s),s)\ T_{\xi (T)+c(T)s}{\mathrm d}W^Q_{T+s},\end{aligned}$$

where

4.6 $$\begin{aligned} Y^{\sigma ,\epsilon ,T}({\textbf{m}}^T,s)=&Y_I^{\sigma ,\epsilon ,T}({\textbf{m}}^T,s)+\epsilon f(T+s)v^T \end{aligned}$$

with

4.7 $$\begin{aligned} Y_{I}^{\sigma ,\epsilon ,T}({\textbf{m}}^T,s)=&2\partial _x\big ((\phi _{c(T)}-\Phi ^T({\textbf{m}}^T,s))v^T\big )+N(v^T)+\epsilon f(T+s)\Phi ^T({\textbf{m}}^T,s)\nonumber \\&-c_d^{\sigma ,\epsilon ,T}({\textbf{m}}^T,s) \partial _c\Phi ^T({\textbf{m}}^T,s)+\Omega _d^{\sigma ,\epsilon ,T}({\textbf{m}}^T,s)\partial _x\Phi ^T({\textbf{m}}^T,s)\nonumber \\&+\tfrac{1}{2}\sigma ^2\Big [\big \Vert Q^{1/2}c_s^{T}({\textbf{m}}^T,s)\big \Vert _{L^2}^2\partial _c^2 +\big \Vert Q^{1/2}\Omega _s^{T}({\textbf{m}}^T,s)\big \Vert _{L^2}^2\partial _x^2\Big ]\Phi ^T({\textbf{m}}^T,s) \end{aligned}$$

and

4.8 $$\begin{aligned} Z^{T}({\textbf{m}}^T,s)[h]&= \big (\Phi ^T({\textbf{m}}^T,s)+v^T\big )h+\Big (-\big \langle c_s^{T}({\textbf{m}}^T,s),h\big \rangle \partial _c\nonumber \\&\quad +\big \langle \Omega _s^{T}({\textbf{m}}^T,s),h\big \rangle \partial _x\Big )\Phi ^T({\textbf{m}}^T,s). \end{aligned}$$

In (4.6) and (4.8), $\partial _c\Phi ^T$ should be interpreted as

$$\begin{aligned}\partial _c\Phi ^T({\textbf{m}}^T,s,x):=\partial _c\phi _{c^T}\big (x+\xi (T)+c(T)s-\xi ^T\big ).\end{aligned}$$

However, we can not rigorously justify (4.5), as $v^T$ is not regular enough for ${\mathcal {L}}_{c(T)}v^T$ to be well-defined. We therefore pass to a mild formulation with respect to the flow generated by the linear equation $w_t={\mathcal {L}}_{c(T)}w$ (see Theorem 2.2).

### Proposition 4.3

(See Appendix B) Assume S1 and S2. For each T,s≥0, the inequalities

$$\begin{aligned}\Vert v^T(s')\Vert _{L^2_w}\le \delta _3 \quad \text {and} \quad c^T(s')\in [\tfrac{1}{2}c_{\textrm{min}},2c_{\textrm{max}}],\quad s' \in [0,s]\end{aligned}$$

imply that

4.9 $$\begin{aligned} v^T(s)=&e^{{\mathcal {L}}_{c(T)}s}v(T)+\int _0^se^{{\mathcal {L}}_{c(T)}(s-s')}Y^{\sigma ,\epsilon ,T}({\textbf{m}}^T(s'),s'){\mathrm d}s'\nonumber \\&+ \sigma \int _0^se^{{\mathcal {L}}_{c(T)}(s-s')}Z^{T}({\textbf{m}}^T(s'),s')\ T_{\xi (T)+c(T)s'}{\mathrm d}W^Q_{T+s'} ,\end{aligned}$$

$$\mathbb {P}$$-almost surely.

It follows straightforwardly that

$$\begin{aligned}{\mathrm d}\big \langle v^T(s),\phi _{c(T)}\big \rangle =&\Big \langle Y^{\sigma ,\epsilon ,T}\big ({\textbf{m}}^T(s),s\big ),\phi _{c(T)}\Big \rangle \ {\mathrm d}s+\sigma \Big \langle Z^T\big ({\textbf{m}}^T(s),s\big )[T_{\xi (T)+c(T)s}{\mathrm d}W_{T+s}^Q],\phi _{c(T)}\Big \rangle ,\\ {\mathrm d}\big \langle v^T(s),\zeta _{c(T)}\big \rangle =&\Big \langle Y^{\sigma ,\epsilon ,T}\big ({\textbf{m}}^T(s),s\big ),\phi _{c(T)}\Big \rangle \ {\mathrm d}s+\sigma \Big \langle Z^T\big ({\textbf{m}}^T(s),s\big )[T_{\xi (T)+c(T)s}{\mathrm d}W_{T+s}^Q],\zeta _{c(T)}\Big \rangle . \end{aligned}$$

For the orthogonality conditions (4.2) to hold, we must have

$$\begin{aligned}\begin{bmatrix} \big \langle Z^T({\textbf{m}}^T,s)[h],\phi _{c(T)}\big \rangle \\ \big \langle Z^T({\textbf{m}}^T,s)[h],\zeta _{c(T)}\big \rangle \end{bmatrix}=0,\quad \forall h \in L^2_Q.\end{aligned}$$

This shows that

$$\begin{aligned} \begin{bmatrix} c^{T}_s({\textbf{m}}^T,s)\\ \Omega ^{T}_s({\textbf{m}}^T,s) \end{bmatrix}= (K^T)^{-1}({\textbf{m}}^T,s)\begin{bmatrix} (\Phi ^T({\textbf{m}}^T,s)+v^T)\phi _{c(T)} \\ ( \Phi ^T({\textbf{m}}^T,s)+v^T)\zeta _{c(T)} \end{bmatrix}, \end{aligned}$$

where

$$\begin{aligned}K^T({\textbf{m}}^T,s)=\begin{bmatrix} \langle \partial _c \Phi ^T({\textbf{m}}^T,s),\phi _{c(T)}\rangle &  -\langle \partial _x \Phi ^T({\textbf{m}}^T,s),\phi _{c(T)}\rangle \\ \langle \partial _c \Phi ^T({\textbf{m}}^T,s),\zeta _{c(T)}\rangle &  -\langle \partial _x \Phi ^T({\textbf{m}}^T,s),\zeta _{c(T)}\rangle \end{bmatrix}.\end{aligned}$$

The drift components $c_d^{\sigma ,\epsilon ,T}$, and $\Omega _d^{\sigma ,\epsilon ,T}$ follow by solving

$$\begin{aligned} \begin{bmatrix} \big \langle Y^{\sigma ,\epsilon ,T}({\textbf{m}}^T,s),\phi _{c(T)}\big \rangle \\ \big \langle Y^{\sigma ,\epsilon ,T}({\textbf{m}}^T,s),\zeta _{c(T)}\big \rangle \end{bmatrix} = 0, \end{aligned}$$

leading to

$$\begin{aligned} c_d^{\sigma ,\epsilon ,T}({\textbf{m}}^T,s)=&c^{0,T}_d({\textbf{m}}^T,s)+\epsilon f(T+s)c^T_f({\textbf{m}}^T,s)+\sigma ^2 c^T_d({\textbf{m}}^T,s) \\ \Omega _d^{\sigma ,\epsilon ,T}({\textbf{m}}^T,s)=&\Omega ^{0,T}_d({\textbf{m}}^T,s)+\epsilon f(T+s)\Omega ^T_f({\textbf{m}}^T,s)+\sigma ^2 \Omega ^T_d({\textbf{m}}^T,s) \end{aligned}$$

with

$$\begin{aligned} \begin{bmatrix} c^{0,T}_d({\textbf{m}}^T,s)\\ \Omega ^{0,T}_d({\textbf{m}}^T,s) \end{bmatrix}=&(K^T)^{-1}({\textbf{m}}^T,s)\begin{bmatrix} \big \langle N(v^T),\phi _{c(T)} \big \rangle \\ \big \langle N(v^T),\zeta _{c(T)} \big \rangle \end{bmatrix}\\  &+2(K^T)^{-1}({\textbf{m}}^T,s)\begin{bmatrix} \Big \langle \partial _x\big ((\phi _{c(T)}-\Phi ^T({\textbf{m}}^T,s))v^T\big ),\phi _{c(T)}\Big \rangle \\ \Big \langle \partial _x\big ((\phi _{c(T)}-\Phi ^T({\textbf{m}}^T,s))v^T\big ),\zeta _{c(T)}\Big \rangle \end{bmatrix},\\ \begin{bmatrix} c^{T}_f({\textbf{m}}^T,s)\\ \Omega ^{T}_f({\textbf{m}}^T,s) \end{bmatrix}=&(K^T)^{-1}({\textbf{m}}^T,s)\begin{bmatrix} \langle \Phi ^T({\textbf{m}}^T,s)+v^T,\phi _{c(T)} \rangle \\ \langle \Phi ^T({\textbf{m}}^T,s)+v^T,\zeta _{c(T)} \rangle \end{bmatrix},\\ \begin{bmatrix} c^{T}_d({\textbf{m}}^T,s)\\ \Omega ^{T}_d({\textbf{m}}^T,s) \end{bmatrix}=&\tfrac{1}{2} \Vert Q^{1/2}c^T_s({\textbf{m}}^T,s)\Vert _{L^2}^2(K^T)^{-1}({\textbf{m}}^T,s)\begin{bmatrix} \Big \langle \partial _c^2 \Phi ^T({\textbf{m}}^T,s),\phi _{c(T)} \Big \rangle \\ \Big \langle \partial _c^2\Phi ^T({\textbf{m}}^T,s) ,\zeta _{c(T)} \Big \rangle \end{bmatrix}\\&+\tfrac{1}{2}\Vert Q^{1/2}\Omega _s^T({\textbf{m}}^T,s)\Vert _{L^2}^2(K^T)^{-1}({\textbf{m}}^T,s)\begin{bmatrix} \Big \langle \partial _x^2 \Phi ^T({\textbf{m}}^T,s),\phi _{c(T)} \Big \rangle \\ \Big \langle \partial _x^2\Phi ^T({\textbf{m}}^T,s) ,\zeta _{c(T)} \Big \rangle \end{bmatrix}. \end{aligned}$$

We now establish control on the local modulation parameters, assuming a-priori control of the quantity

4.10 $$\begin{aligned} R^T_w(s):=\Vert v^T(s)\Vert _{L^2_w}+\big |c(T)-c^T(s)\big |+\big |\xi (T)+c(T)s-\xi ^T(s)\big |.\end{aligned}$$

### Lemma 4.4

Assuming S1 and S2, there exist constants $\delta _5, C_5>0$ such that following holds true. For all T,s≥0, the bounds

$$\begin{aligned}c(T),c^T(s)\in [\tfrac{1}{2}c_{\textrm{min}},2c_{\textrm{max}}]\quad \text {and}\quad R^T_w(s)\le \delta _5\end{aligned}$$

imply

4.11 $$\begin{aligned} \big \Vert Q^{1/2}c^T_s({\textbf{m}}^T(s),s)\big \Vert _{L^2}+\big \Vert Q^{1/2}\Omega ^T_s({\textbf{m}}^T(s),s)\big \Vert _{L^2}\le&C_5\big (1+\Vert v^T(s)\Vert _{L^2_w}\big ), \end{aligned}$$

4.12 $$\begin{aligned} \big |c_d^{T,0}({\textbf{m}}^T(s),s)\big |+\big |\Omega _d^{T,0}({\textbf{m}}^T(s),s)\big | \le&C_5 R^T_w(s)\Vert v^T(s)\Vert _{L^2_w}, \end{aligned}$$

4.13 $$\begin{aligned} \big |c_f({\textbf{m}}^T(s),s)\big |+\big |\Omega _f({\textbf{m}}^T(s),s)\big |\le&C_5\big (1+\Vert v^T(s)\Vert _{L^2_w}\big ), \end{aligned}$$

4.14 $$\begin{aligned} \big |c_d({\textbf{m}}^T(s),s)\big |+\big |\Omega _d({\textbf{m}}^T(s),s)\big |\le&C_5\big (1+\Vert v^T(s)\Vert _{L^2_w}\big ). \end{aligned}$$

### Proof

As in the proof of Lemma 3.3, note that

$$\begin{aligned}K^T({\textbf{m}}^T(0),0)=\begin{bmatrix} \langle \partial _c \phi _{c(T)},\phi _{c(T)}\rangle &  0\\ \langle \partial _c \phi _{c(T)},\zeta _{c(T)}\rangle &  -\langle \partial _x \phi _{c(T)},\zeta _{c(T)}\rangle \end{bmatrix}.\end{aligned}$$

In particular, we can find constants ${\tilde{C}}_1,{\tilde{C}}_2>0$ that ensure $\Vert A^{-1}\Vert _{\textrm{op}}\le {\tilde{C}}_2$ for all $A\in {\mathbb {R}}^{2\times 2}$ which satisfy

$$\begin{aligned}\Big |A_{ij}-K_{ij}^T({\textbf{m}}^T(0),0)\Big |\le {\tilde{C}}_1, \quad (i,j) \in \{1,2\}^2.\end{aligned}$$

Recalling that $\Phi ^T({\textbf{m}}^T(s),s)=\phi _{c^T(s)}\big (\cdot +\xi (T)+c(T)s-\xi ^T(s)\big )$, the Lipschitz properties of $\phi _c$ imply

$$\begin{aligned} \Big |K_{ij}^T({\textbf{m}}^T(s),s)-K_{ij}^T({\textbf{m}}^T(0),0)\Big |\le&{\tilde{C}}_3\Big (\big |\xi (T)+c(T)s-\xi ^T(s)\big |+\big |c^T(s)-c(T)\big |\Big ) \end{aligned}$$

for all $(i,j)\in \{1,2\}^2$. In case $\delta _5\le {\tilde{C}}_3^{-1}{\tilde{C}}_1$, it follows that $\big \Vert (K^T)^{-1}({\textbf{m}}^T,s)\big \Vert _{\textrm{op}}\le {\tilde{C}}_2$. Estimating

$$\begin{aligned} \Big |\Big \langle \partial _x\big ((\phi _{c(T)}-\Phi ^T({\textbf{m}}^T,s))v^T(s)\big ),\phi _{c(T)}\Big \rangle \Big |+ \Big |\Big \langle \partial _x\big ((\phi _{c(T)}-\Phi ^T({\textbf{m}}^T(s),s))v^T(s)\big ),\zeta _{c(T)}\Big \rangle \Big |\\ \le \big (\Vert \partial _x\phi _{c(T)}\Vert _{\infty }+\Vert \partial _x\phi _{c(T)}\Vert _{\infty }\big )\Vert \phi _{c(T)}-\Phi ^T({\textbf{m}}^T(s),s)\Vert _{L^2_{-w}}\Vert v^T(s)\Vert _{L^2_w} \end{aligned}$$

establishes (4.12). The estimates (4.11), (4.13) and (4.14) follow by applying the Cauchy-Schwarz inequality. □

We conclude this section with a result on the deterministic integral in (4.9). This is provided by Corollary 4.6 below and forms the basis of our stability arguments. Our estimates for the deterministic terms (σ=0) in (4.9) are similar to those in [25], save for our treatment of the nonlinearity $N(v^T)=-\partial _x(v^T)^2$. Here we follow the approach of Mizumachi and Tzvetkov [23] which uses property (2.4). This allows us to control the nonlinear term based on the condition that $\Vert v^T\Vert _{L^2}$ is small. This is an improvement over the standard argument used in [25], which requires control of $\Vert \partial _x v^T\Vert _{L^2}$ and consequently a more cumbersome energy argument.

### Lemma 4.5

Assuming S1 and S2, there exists a constant $C_6>0$ such that for each T,s≥0 the inequalities

$$\begin{aligned}c(T),c^T(s)\in [\tfrac{1}{2}c_{\textrm{min}},2c_{\textrm{max}}]\quad \text {and}\quad R^T_w(s)\le \delta _5\end{aligned}$$

imply

$$\begin{aligned}\Big \Vert Y_I^{\sigma ,\epsilon ,T}({\textbf{m}}^T(s),s)\Big \Vert _{L^1_w} \le&C_{6}\sigma ^2\big (1+\Vert v^T(s)\Vert _{L^2_w}\big )^2+C_6\epsilon \\  &+C_6\Big (\Vert v^T(s)\Vert _{L^2}+R^T_w(s)\Big )\Vert v^T(s)\Vert _{H^1_w}. \end{aligned}$$

### Proof

The various terms in $Y_I^{\sigma ,\epsilon ,T}\big ({\textbf{m}}^T(s),s\big )$ – see (4.7) – can be controlled as follows. The inequality

$$\begin{aligned}&\Big \Vert 2\partial _x\big ((\phi _{c(T)}-\Phi ^T({\textbf{m}}^T(s),s)) v^T \big )\Big \Vert _{L^1_w}\le 2\big \Vert \phi _{c(T)}-\Phi ^T({\textbf{m}}^T(s),s)\big \Vert _{H^1}\big \Vert v^T(s)\big \Vert _{H^1_w}\\ \le&{\tilde{C}}_1\Big (\big |c(T)-c^T(s)\big |+\big |\xi (T)+c(T)s-\xi ^T(s)\big |\Big ) \big \Vert v^T(s)\big \Vert _{H^1_w} \end{aligned}$$

follows from the fact that $c \mapsto \phi _c(x)+\partial _x\phi _c(x)$ is Lipschitz from $[0,c_{\textrm{max}}]$ to $L^2$. Via Lemma 4.4 we find

$$\begin{aligned}&\Big \Vert \Big [-c_d^{\sigma ,\epsilon ,T}({\textbf{m}}^T(s),s) \partial _c+\Omega _d^{\sigma ,\epsilon ,T}({\textbf{m}}^T(s),s)\partial _x \Big ]\Phi ^T({\textbf{m}}^T(s),s)\Big \Vert _{L^1_w}\\&\quad \le {\tilde{C}}_2\Big |c_d^{\sigma ,\epsilon ,T}({\textbf{m}}^T(s),s) +\Omega _d^{\sigma ,\epsilon ,T}({\textbf{m}}^T(s),s) \Big | \\&\quad \le {\tilde{C}}_2C_5R^T_w(s)\Vert v^T(s)\Vert _{L^2_w}, \end{aligned}$$

where ${\tilde{C}}_2$ is a constant large enough to ensure

$$\begin{aligned}\Vert \partial _c\Phi ^T({\textbf{m}}^T(s),s)\Vert _{L^1_w}+\Vert \partial _x\Phi ^T({\textbf{m}}^T(s),s)\Vert _{L^1_w}\le {\tilde{C}}_2.\end{aligned}$$

Similarly,

$$\begin{aligned}\big \Vert Q^{1/2}c_s^{T}({\textbf{m}}^T(s),s)\big \Vert _{L^2}^2\big \Vert \partial _c^2\Phi ^T({\textbf{m}}^T(s),s)\big \Vert _{L^1_w} \le&{\tilde{C}}_3\big (1+\Vert v^T(s)\Vert _{L^2_w}\big )^2, \end{aligned}$$

and

$$\begin{aligned} \big \Vert Q^{1/2}\Omega _s^{T}({\textbf{m}}^T(s),s)\big \Vert _{L^2}^2\big \Vert \partial _x^2\Phi ^T({\textbf{m}}^T(s),s)\big \Vert _{L^1_w} \le&{\tilde{C}}_3\big (1+\Vert v^T(s)\Vert _{L^2_w}\big )^2. \end{aligned}$$

Lastly,

$$\begin{aligned}\Big \Vert \partial _x\big ((v^T(s))^2\big )\Big \Vert _{L^1_w}\le 2\Vert v^T(s)\Vert _{L^2}\Vert v^T(s)\Vert _{H^1_w}, \end{aligned}$$

and

$$\begin{aligned}\big \Vert \epsilon f(T+s)\Phi ^T({\textbf{m}}^T(s),s)\big \Vert _{L^1_w}\le {\tilde{C}}_4\epsilon .\end{aligned}$$

□

### Corollary 4.6

Assuming S1 and S2, there exists a constant $C_7>0$ such that for each T,s≥0 the inequalities

$$\begin{aligned} c_{\textrm{min}}\le c^T(s') \le c_{\textrm{max}} \quad \text {and} \quad R^T_w(s')\le \delta _5,\quad s'\in [0,s]\end{aligned}$$

imply that

4.15 $$\begin{aligned} \Big \Vert \int _0^s e^{{\mathcal {L}}_{c(T)}(s-s')}Y^{\sigma ,\epsilon ,T}\big ({\textbf{m}}^T(s'),s'){\mathrm d}s'\Big \Vert _{H^1_w} \le&C_7s\sup _{0\le s'\le s}\Big (\Vert v^T(s')\Vert _{L^2}+R^T_w(s')\Big ) \big \Vert v^T(s')\big \Vert _{H^1_w}\nonumber \\&+C_7(\sigma ^2+\epsilon )s, \end{aligned}$$

holds $\mathbb {P}$-almost surely.

### Proof

Consider the decomposition

$$\begin{aligned}Y^{\sigma ,\epsilon ,T}\big ({\textbf{m}}^T(s'),s'\big )=\underbrace{Y_I^{\sigma ,\epsilon ,T}\big ({\textbf{m}}^T(s'),s'\big )}_{ \text {in } L^1_w}+\underbrace{\epsilon f(T+s')v^T(s')}_{ \text {in } L^2_w}.\end{aligned}$$

Both terms are contained in the stable subspace of $\big \{e^{{\mathcal {L}}_{c(T)}t}\big \}_{t\ge 0}$. We have via (2.3)

$$\begin{aligned}  &   \Big \Vert \int _0^s e^{{\mathcal {L}}_{c(T)}(s-s')}\big [f(T+s')v^T(s')\big ]{\mathrm d}s' \Big \Vert _{H^1_w}\\  &   \quad \le M \int _0^s e^{-b(s-s')}(s-s')^{-1/2}{\mathrm d}s' \sup _{s'\in [0,s]}\big \Vert v^T(s')\big \Vert _{L^2_w}.\end{aligned}$$

Here, *b* and *M* are the constants appearing in the semigroup-bounds (2.3) and (2.4). We remark also that

$$\begin{aligned}\sup _{s\ge 0}\int _0^s e^{-b(s-s')}(s-s')^{-1/2}{\mathrm d}s'<\infty .\end{aligned}$$

Using (2.4), on the other hand, we have

$$\begin{aligned}&\Big \Vert \int _0^s e^{{\mathcal {L}}_{c(T)}(s-s')}\Big [Y_I^{\sigma ,\epsilon ,T}\big ({\textbf{m}}^T(s'),s'\big )\Big ]{\mathrm d}s' \Big \Vert _{H^1_w} \\ \le&M \int _0^s e^{-b(s-s')}(s-s')^{-3/4}{\mathrm d}s\ \sup _{s'\in [0,s]}\Big \Vert Y_I^{\sigma ,\epsilon ,T}\big ({\textbf{m}}^T(s'),s'\big )\Big \Vert _{L^1_w},\end{aligned}$$

where also

$$\begin{aligned}\sup _{s\ge 0}\int _0^s e^{-b(s-s')}(s-s')^{-3/4}{\mathrm d}s<\infty .\end{aligned}$$

The result now follows by applying Lemma 4.5. □

## Weighted norm control

In this section, we control the local remainder $v^T$ on time intervals [0,ΔT]. We do so by exploiting the stability properties of the linear flow $\{e^{{\mathcal {L}}_{c(T)}t}\}_{t\ge 0}$ on weighted spaces. As pointed out, control of the local remainder $v^T$ transfers to the global remainder *v* via Lemma 4.2. The main result of this section is Proposition 5.1 below, which bounds the probability that $v^T$ grows large on a time interval [0,ΔT]. We show that $\Vert v^T\Vert _{H^1_w}$ only grows large with small probability, provided that

- the soliton amplitude remains within fixed bounds;
- the unweighted $L^2$-norm of $v^T$ remains small;
- the difference between global and local modulation parameters remains small.

An important ingredient for ensuring that we can repeat our stability argument on time intervals of size ΔT is the exponential decay of the semigroup after time ΔT in the first term of (5.3). We thus require that ΔT is large enough to guarantee significant decay. We formulate this in the following condition, and fix constants $\delta _*, \eta _0$ for later use.

**C1**: *The constants* $\Delta T,\delta _*>0$ and $\eta _0>0$ satisfy

- $$\Delta T= \log (6M)/b$$;
- $$\delta _*\le \min \big \{\frac{1}{216 MC_7(\Delta T +\Delta T^2)},\frac{1}{4}c_{\textrm{min}},\frac{1}{2}c_{\textrm{max}},\frac{1}{e^2+3C_4}\big \}$$;
- $$\eta _0\le \min \big \{\delta _*,\delta _5,1,\frac{1}{8C_5(\Delta T+\Delta T^2)}\big \}$$.

The constants $C_4, C_5, \delta _5$ and $C_7$ have been introduced in Lemma 4.2, Lemma 4.4 and Corollary 4.6, respectively. The constants *b*, *M* are introduced in the semigroup-bounds (2.3). To keep track of the weighted norm $\Vert v^T(s)\Vert _{H^1_w}$, we define for each T,η>0 the stopping time $\tau ^T_{\textrm{st}}$ as

$$\begin{aligned} \tau ^T_{\textrm{st}}(\eta )=\sup \big \{s\ge 0:\big \Vert v^T(s)\big \Vert _{H^1_w}\le \eta \big \}. \end{aligned}$$

We furthermore introduce stopping times $\tau ^T_c, \tau ^T_{\textrm{en}}, \tau ^{T}_{\textrm{amp}}$ and $\tau ^{T}_{\textrm{pos}}$ which encode the conditions for stability:

$$\begin{aligned} \tau ^T_c=&\sup \big \{s\ge 0:c(T+s)\in [\tfrac{1}{2}c_{\textrm{min}},2c_{\textrm{max}}] \big \};\\ \tau ^T_{\textrm{en}}(\eta )=&\sup \big \{s\ge 0:\big \Vert v^T(s)\big \Vert _{L^2}\le \eta \big \};\\ \tau ^{T}_{\textrm{amp,1}}(\eta ) =&\sup \big \{s\ge 0:\big |c(T)-c^T(s)\big |\le \delta _*\eta \}; \\ \tau ^{T}_{\textrm{amp,2}}(\eta ) =&\sup \big \{s\ge 0:\big |c(T+s)-c^T(s)\big |\le \delta _*\eta \}; \\ \tau ^{T}_{\textrm{pos,1}}(\eta ) =&\sup \big \{s\ge 0:\big |\xi (T)+c(T)s-\xi ^T(s)\big |\le 2\Delta T \delta _*\eta \big \};\\ \tau ^{T}_{\textrm{pos,2}}(\eta ) =&\sup \big \{s\ge 0:\big |\xi (T+s)-\xi ^T(s)\big |\le 2\Delta T \delta _*\eta \big \}, \end{aligned}$$

and we define

$$\begin{aligned} \tau ^T_{\textrm{mod}}(\eta )=\tau ^{T}_{\textrm{amp},1}(\eta )\wedge \tau ^{T}_{\textrm{amp},2}(\eta )\wedge \tau ^{T}_{\textrm{pos,1}}(\eta )\wedge \tau ^{T}_{\textrm{pos,2}}(\eta ). \end{aligned}$$

Our result is then as follows.

### Proposition 5.1

(Short-time control) Assuming S1, S2 and C1, there exist constants $\delta _9> 0$ and $C_9\ge 1$ such that the following holds true. For all $\eta \in [0,\eta _0]$, $C_9\sigma ,C_9\epsilon \in [0,\eta ]$ and T≥0 the events

$$\begin{aligned} {\mathcal {E}}_1=\big \{\tau ^T_{\textrm{st}}(\eta ) \le \Delta T\wedge \tau ^T_{\textrm{en}}(\delta _*)\wedge \tau ^T_{\textrm{mod}}(\eta )\wedge \tau ^T_c\big \} \end{aligned}$$

and

$$\begin{aligned} {\mathcal {E}}_2=\big \{\big \Vert v^T(\Delta T)\big \Vert _{H^1_w}\ge \tfrac{\eta }{9M}\big \}\cap \big \{\tau ^T_{\textrm{st}}(\eta )\wedge \tau ^T_{\textrm{en}}(\delta _*)\wedge \tau ^T_{\textrm{mod}}(\eta )\wedge \tau ^T_c\ge \Delta T\big \} \end{aligned}$$

satisfy

5.1 $$\begin{aligned} \mathbb {P}\Big [ {\mathcal {E}}_1\cap \big \{\Vert v(T)\Vert _{H^1_w}\le \tfrac{\eta }{3M} \big \}\Big ]+\mathbb {P}\Big [ {\mathcal {E}}_2\cap \big \{\big \Vert v(T)\big \Vert _{H^1_w}\le \tfrac{\eta }{3M} \big \}\Big ]\le e^{-\delta _9\eta ^2/\sigma ^2}. \end{aligned}$$

Our main tool for establishing Proposition 5.1 is based on the results of Section 4. We recall that the constant $C_7$ was introduced in Corollary 4.6.

### Lemma 5.2

Assume S1 and S2. For all $\sigma ,\epsilon ,T,s,\delta \ge 0$, each $\delta _*>0$ and $\eta \in [0,\min \{\delta _*,\delta _5,1\}]$ that satisfy

5.2 $$\begin{aligned} s+s^2\le \frac{\delta }{6C_7\delta _*}\quad \text {and} \quad \max \{\sigma ^2s, \epsilon s\}\le \frac{\delta \eta }{3C_7},\end{aligned}$$

the inequalities

$$\begin{aligned} \big \Vert v^T(s')\big \Vert _{H^1_w}\le \eta \quad \text {and} \quad \Vert v^T(s')\Vert _{L^2}+R^T_w(s')\le \delta _*(1+2s),\quad s'\in [0,s] \end{aligned}$$

imply

5.3 $$\begin{aligned}&\big \Vert v^T(s)\big \Vert _{H^1_w}\le Me^{-b s} \big \Vert v(T)\big \Vert _{H^1_w}+\delta \eta \nonumber \\&\quad +\sigma \Big \Vert \int _0^se^{{\mathcal {L}}_{c(T)}(s-s')}Z^{T}\big ({\textbf{m}}^T(s'),s'\big )\ T_{\xi (T)+c(T)s'}{\mathrm d}W^Q_{T+s'}\Big \Vert _{H^1_w}. \end{aligned}$$

### Proof

We apply Corollary 4.6 to estimate (4.9):

$$\begin{aligned} \big \Vert v^T(s)\big \Vert _{H^1_w}\le&Me^{-b s} \big \Vert v(T)\big \Vert _{H^1_w}+C_7(\sigma ^2+\epsilon )s+2C_7\delta _*(s+s^2)\eta \nonumber \\&+\sigma \Big \Vert \int _0^se^{{\mathcal {L}}_{c(T)}(s-s')}Z^{T}\big ({\textbf{m}}^T(s'),s'\big )\ T_{\xi (T)+c(T)s'}{\mathrm d}W^Q_{T+s'}\Big \Vert _{H^1_w}. \end{aligned}$$

The result hence follows from the assumptions (5.2). □

We remark that the constants $\delta _*,\eta _0$ introduced in C1 ensure that Lemma 5.2 may be applied on the interval [0,ΔT]. With Lemma 5.2 established, we furthermore require control of the stochastic convolution present in (5.3). Our main tool for doing so is the following.

### Theorem 5.3

(Gaussian tails of stochastic convolution, see Appendix B) There exists a constant K>0 such that the following holds true. Suppose that $\big \{S(t)\big \}_{t\ge 0}$ is a $C_0$-semigroup on a Hilbert space ${\mathcal {H}}$ satisfying

$$\begin{aligned} \sup _{t\ge 0}\big \Vert S(t)\big \Vert _{{\mathcal {L}}({\mathcal {H}})}\le M \end{aligned}$$

for some M≥1, and $g\in L^p(\Omega ;L^p(0,T;\textrm{HS}(L_Q^2,{\mathcal {H}}))$ satisfies

$$\begin{aligned} \int _0^T\mathbb {E}\Big [ \big \Vert g(t)\big \Vert ^p_{\textrm{HS}(L^2_Q,{\mathcal {H}})}\Big ]{\mathrm d}t\le B^pT, \quad p>2 \end{aligned}$$

for some B≥0. Then, for $\lambda > eBKM\sqrt{ T}$ we have the inequality

$$\begin{aligned} \mathbb {P}\Big [ \sup _{t\in [0,T]}\big \Vert \int _0^t S(t-s)g(s){\mathrm d}W^Q_s \big \Vert _{{\mathcal {H}}} \ge \lambda \Big ] \le e^{-(eBKM)^{-2}\lambda ^2/T}. \end{aligned}$$

In the sequel, we will apply Theorem 5.3 to stochastic convolutions with respect to the semigroup $\{e^{{\mathcal {L}}_{c(T)}t}\}_{t\ge 0}$, as well as ordinary stochastic integrals. In the latter case, we take the semigroup in Theorem 5.3 to be the trivial semigroup, i.e. the identity operator. Below, we explicitly compute the norms of various Hilbert-Schmidt operators used in the sequel.

### Lemma 5.4

Assuming S1 and S2, there exists a constant $C_{10}$ such that for all ${\tilde{\xi }}\in {\mathbb {R}}$, $g\in L^2$ and $h\in H^1_w$, we have

$$\begin{aligned} \Vert hT_{{\tilde{\xi }}}\cdot \Vert _{\textrm{HS}(L^2_Q,H^1_w)}\le&C_{10}\Vert h\Vert _{H^1_w},\\ \big \Vert \langle g,T_{{\tilde{\xi }}}\cdot \rangle h\big \Vert _{\textrm{HS}(L^2_Q,H^1_w)}=&\Vert Q^{1/2}g\Vert _{L^2}\Vert h\Vert _{H^1_w},\\ \big \Vert \langle g,T_{{\tilde{\xi }}} \cdot \rangle \big \Vert _{\textrm{HS}(L_Q^2,{\mathbb {R}})}=&\Vert Q^{1/2}g\Vert _{L^2}, \end{aligned}$$

while for all $g\in L^1$ we have

$$\begin{aligned} \big \Vert \langle g,T_{{\tilde{\xi }}} \cdot \rangle \big \Vert _{\textrm{HS}(L_Q^2,{\mathbb {R}})}\le&C_{10}\Vert g\Vert _{L^1}. \end{aligned}$$

### Proof

We compute that pointwise multiplication with a function $h\in H^1_w$ leads to the identity

$$\begin{aligned} \Vert hT_{{\tilde{\xi }}}\cdot \Vert ^2_{\textrm{HS}(L^2_Q,H^1_w)}=&\sum _{k=0}^\infty \Vert hQ^{1/2}e_k\Vert ^2_{H^1_w} \\ =&\sum _{k=0}^\infty \int _{{\mathbb {R}}} e^{2wx}\big (h^2(x)+h_x^2(x)\big )\big \langle q_{1/2}(x-\cdot ),e_k\big \rangle ^2\\&+e^{2wx}h^2(x)\big \langle q'_{1/2}(x-\cdot ),e_k\big \rangle ^2 {\mathrm d}x \\ =&\Vert q_{1/2}\Vert _{L^2}^2\Vert h\Vert _{H^1_w}^2+\Vert q'_{1/2}\Vert _{L^2}^2\Vert h\Vert _{L^2_w}^2. \end{aligned}$$

Inner products against a function $g\in L^2$ lead to

$$\begin{aligned} \big \Vert \langle g,T_{{\tilde{\xi }}} \cdot \rangle \big \Vert ^2_{\textrm{HS}(L_Q^2,{\mathbb {R}})}=&\sum _{k=0}^\infty \big |\langle g,T_{{\tilde{\xi }}} Q^{1/2}e_k\rangle \big |^2=\Vert Q^{1/2}g\Vert _{L^2}^2, \end{aligned}$$

and hence also

$$\begin{aligned}&\big \Vert \langle g,T_{{\tilde{\xi }}}\cdot \rangle h\big \Vert ^2_{\textrm{HS}(L^2_Q,H^1_w)}=\sum _{k=0}^\infty \big \Vert \langle g,Q^{1/2}e_k\rangle h\big \Vert ^2_{H^1_w}\\&\quad = \big \Vert \langle g,T_{{\tilde{\xi }}} \cdot \rangle \big \Vert ^2_{\textrm{HS}(L_Q^2,{\mathbb {R}})}\Vert h\Vert ^2_{H^1_w}=\Vert Q^{1/2}g\Vert _{L^2}^2\Vert h\Vert ^2_{H^1_w}. \end{aligned}$$

For $$g\in L^1$$:

$$\begin{aligned} \big \Vert \langle g,T_{{\tilde{\xi }}} \cdot \rangle \big \Vert ^2_{\textrm{HS}(L_Q^2,{\mathbb {R}})}=\Vert Q^{1/2}g\Vert _{L^2}^2\le \Vert \sqrt{{\hat{q}}}\Vert _{L^2}^2\Vert {\hat{g}}\Vert _\infty ^2=\Vert {\hat{q}}\Vert _{L^1}\Vert g\Vert ^2_{L^1}. \end{aligned}$$

Here we note that ${\hat{q}}\in L^1$, since *q* is assumed to be an element of $H^1$ in S1 and

$$\begin{aligned} \Vert {\hat{q}}\Vert _{L^1}^2\le \int _{{\mathbb {R}}}(1+|\omega |^2)|{\hat{q}}(\omega )|^2{\mathrm d}\omega \times \int _{{\mathbb {R}}}\frac{1}{1+|\omega |^2}{\mathrm d}\omega \le {\tilde{C}}_1\Vert q\Vert _{H^1}^2. \end{aligned}$$

□

With these preliminaries in place, control on the stochastic integral in (5.3) is provided by the following lemma.

### Lemma 5.5

Assuming S1 and S2, there exists a constant $C_{11}>0$ so that for each T,s≥0 and ${\tilde{\xi }} \in {\mathbb {R}}$, the bounds

$$\begin{aligned} c(T),c^T(s)\in [\tfrac{1}{2}c_{\textrm{min}},2c_{\textrm{max}}]\quad \text {and}\quad R^T_w(s)\le \delta _5 \end{aligned}$$

imply

$$\begin{aligned}\Big \Vert Z^{T}\big ({{\textbf{m}}}^T(s),s\big )T_{{\tilde{\xi }}}\Big \Vert _{\textrm{HS}(L^2_Q,H^1_w)}\le C_{11}\Big (1+\big \Vert {v}^T(s)\big \Vert _{H^1_w}\Big ). \end{aligned}$$

### Proof

From (4.8), a straightforward application of the triangle inequality yields the $\mathbb {P}$-a.s. bound

$$\begin{aligned} \big \Vert Z^{T}({{\textbf{m}}}^T(s),s)T_{{\tilde{\xi }}}\big \Vert _{\textrm{HS}(L^2_Q,H^1_w)}\le&\big \Vert (\Phi ^T({{\textbf{m}}}^T(s),s) +{v}^T(s))T_{{\tilde{\xi }}}\cdot \big \Vert _{\textrm{HS}(L^2_Q,H^1_w)}\\&+\Big \Vert \big \langle c_s^{T}({{\textbf{m}}}^T(s),s),T_{{\tilde{\xi }}}\cdot \big \rangle \partial _c\Phi ^T({{\textbf{m}}}^T(s),s)\Big \Vert _{\textrm{HS}(L^2_Q,H^1_w)}\\&+\Big \Vert \big \langle \Omega _s^{T}({{\textbf{m}}}^T(s),s),T_{{\tilde{\xi }}}\cdot \big \rangle \partial _x\Phi ^T({{\textbf{m}}}^T(s),s)\Big \Vert _{\textrm{HS}(L^2_Q,H^1_w)}. \end{aligned}$$

Applying Lemma 5.4 yields

$$\begin{aligned}  &   \big \Vert Z^{T}({{\textbf{m}}}^T(s),s)T_{{\tilde{\xi }}}\big \Vert _{\textrm{HS}(L^2_Q,H^1_w)}\le {\tilde{C}}_4\sigma \Big (1+\big \Vert Q^{1/2}c_s^T({{\textbf{m}}}^T(s),s)\big \Vert _{L^2}\\  &   \quad +\big \Vert Q^{1/2}\Omega _s^T({{\textbf{m}}}^T(s),s)\big \Vert _{L^2}+\Vert {v}^T(s)\Vert _{H^1_w}\Big ) \end{aligned}$$

and applying Lemma 4.4 provides the result. □

Having established control on $v^T$ via Lemma 5.2 and Lemma 5.5, we are ready to prove the main result of this section: Proposition 5.1.

### Proof of Proposition 5.1

Let us fix $C_9=18C_7\Delta T$. Writing

$$\begin{aligned} \tau =\min \big \{\tau ^T_{\textrm{st}}(\eta ),\tau ^T_{\textrm{en}}(\delta _*),\tau ^T_{\textrm{mod}}(\eta ),\tau ^T_c,\Delta T\big \}, \end{aligned}$$

we may establish control of $\Vert v^T(\tau )\Vert _{H^1_w}$ by applying Lemma 5.2 with δ=1/3:

$$\begin{aligned} \big \Vert v^T(\tau )\big \Vert _{H^1_w}\le&M \big \Vert v(T)\big \Vert _{H^1_w}+\eta /3+\sigma \sup _{0\le s\le \Delta T}I(s), \end{aligned}$$

where we have abbreviated

$$\begin{aligned} I(s)=\Big \Vert \int _0^s e^{{\mathcal {L}}_{c(T)}(s-s')}1_{[0,\tau ]}(s')Z^{T}\big ({\textbf{m}}^T(s'),s'\big )\ T_{\xi (T)+c(T)s'}{\mathrm d}W^Q_{T+s'}\Big \Vert _{H^1_w}. \end{aligned}$$

Suppose now that $\tau ^T_{\textrm{st}}(\eta )\le \Delta T$, meaning that the stopping time $\tau ^T_{\textrm{st}}$ is activated because the bound η is reached on [0,ΔT], while also

$$\begin{aligned}\tau ^T_{\textrm{st}}(\eta ) \le \min \big \{\tau ^T_{\textrm{en}}(\delta _*),\tau ^T_{\textrm{mod}}(\eta ),\tau ^T_c\big \}.\end{aligned}$$

Then,

$$\begin{aligned} \eta =&\big \Vert v^T\big (\tau ^T_{\textrm{st}}(\eta )\big )\big \Vert _{H^1_w}=\big \Vert v^T(\tau )\big \Vert _{H^1_w} \le M\big \Vert v(T)\big \Vert _{H^1_w}+\eta /3+\sigma \sup _{0\le s\le \Delta T}I(s). \end{aligned}$$

It follows that the event ${\mathcal {E}}_1\cap \{\Vert v(T)\Vert _{H^1_w}\le \tfrac{\eta }{3M}\}$ can only happen if

$$\begin{aligned}\sigma \sup _{0\le s\le \Delta T}I(s)\ge \eta /3,\end{aligned}$$

so that

$$\begin{aligned} \mathbb {P}\Big [{\mathcal {E}}_1\cap \big \{\Vert v(T)\Vert _{H^1_w}\le \tfrac{\eta }{3M} \big \}\Big ]\le \mathbb {P}\Big [\sigma \sup _{0\le s\le \Delta T}I(s)\ge \eta /3\Big ]. \end{aligned}$$

To control this probability, note that Lemma 5.5 implies that $\mathbb {P}$-almost surely

$$\begin{aligned} 1_{[0,\tau ]}(s')\Big \Vert Z^{T}\big ({\textbf{m}}^T(s'),s'\big )T_{\xi (T)+c(T)s'}\Big \Vert _{\textrm{HS}(L^2_Q,H^1_w)}\le C_{11}(1+\eta ), \quad s'\in [0,\Delta T].\end{aligned}$$

By applying Theorem 5.3 with the semigroup $\{e^{{\mathcal {L}}_{c(T)t}}\}_{t\ge 0}$ restricted to its stable subspace, and by increasing $C_9$ to meet $C_9\ge 2eC_7K\sqrt{\Delta T}$, we find

$$\begin{aligned} \mathbb {P}\Big [\sigma \sup _{0\le s\le \Delta T}I(s)\ge \eta /3\Big ]\le e^{-\delta _9\eta ^2/\sigma ^2}, \end{aligned}$$

with

$$\begin{aligned}\delta _9=\big (2e C_7M^{-1}K\big )^{-2}/\Delta T.\end{aligned}$$

It remains to establish the same bound on ${\mathcal {E}}_2$. To this end, suppose that

$$\begin{aligned}\tau =\Delta T\quad \text {and}\quad \Vert v(T)\Vert _{H^1_w}\le \tfrac{\eta }{3M},\quad \text {but}\quad \big \Vert v^T(\Delta T)\big \Vert _{H^1_w}\ge \tfrac{\eta }{9M}.\end{aligned}$$

Upon increasing $C_9$ to ensure that Lemma 5.2 may be applied on [0,ΔT] with δ=1/36M, we obtain

$$\begin{aligned}\tfrac{\eta }{9M} \le \big \Vert v^T(\Delta T)\big \Vert _{H^1_w}\le \tfrac{1}{6}\big \Vert v(T)\big \Vert _{H^1_w}+\tfrac{\eta }{36M}+\sigma I(\Delta T),\end{aligned}$$

where C1 produces the factor $\tfrac{1}{6}$ above. The conclusion follows, upon decreasing $\delta _9$ to

$$\begin{aligned}\delta _9=\big (72e C_7K\big )^{-2}/\Delta T,\end{aligned}$$

via the tail bound

$$\begin{aligned} \mathbb {P}\Big [{\mathcal {E}}_2\cap \big \Vert v(T)\big \Vert _{H^1_w}\le \tfrac{\eta }{3M}\Big ]\le \mathbb {P}\Big [\sigma I(\Delta T)\ge \tfrac{\eta }{36M}\Big ]\le e^{-\delta _9 \eta ^2/\sigma ^2}. \end{aligned}$$

□

## Local control of modulation parameters

In this section, we establish several facts regarding the modulation parameters c,ξ and their local counterparts. Our goal is to address one of the conditions for stability formulated in Section 5: an estimate on the local modulation parameters, encoded by the stopping time

$$\begin{aligned} \tau ^T_{\textrm{mod}}(\eta )=\tau ^{T}_{\textrm{amp},1}(\eta )\wedge \tau ^{T}_{\textrm{amp},2}(\eta )\wedge \tau ^{T}_{\textrm{pos,1}}(\eta )\wedge \tau ^{T}_{\textrm{pos,2}}(\eta )\end{aligned}$$

introduced in Section 5, where

$$\begin{aligned} \tau ^{T}_{\textrm{amp,1}}(\eta ) =&\sup \big \{s\ge 0:\big |c(T)-c^T(s)\big |\le \delta _*\eta \}; \\ \tau ^{T}_{\textrm{amp,2}}(\eta ) =&\sup \big \{s\ge 0:\big |c(T+s)-c^T(s)\big |\le \delta _*\eta \}; \\ \tau ^{T}_{\textrm{pos,1}}(\eta ) =&\sup \big \{s\ge 0:\big |\xi (T)+c(T)s-\xi ^T(s)\big |\le 2\Delta T\delta _*\eta \big \};\\ \tau ^{T}_{\textrm{pos,2}}(\eta ) =&\sup \big \{s\ge 0:\big |\xi (T+s)-\xi ^T(s)\big |\le 2\Delta T\delta _*\eta \big \}. \end{aligned}$$

We show that the probability that one of the stopping times above is activated on [0,ΔT], while the local perturbation $v^T$ is small and the global amplitude is within the bounds $[c_{\textrm{min}},c_{\textrm{max}}]$, satisfies an exponential tail estimate. Recall from Section 5 that

$$\begin{aligned} \tau ^T_c=&\sup \big \{s\ge 0:c(T+s)\in [\tfrac{1}{2}c_{\textrm{min}},2c_{\textrm{max}}] \big \},\\ \tau ^T_{\textrm{st}}(\eta )=&\sup \big \{s\ge 0:\big \Vert v^T(s)\big \Vert _{H^1_w}\le \eta \big \}, \end{aligned}$$

and let us furthermore introduce the stopping time

$$\begin{aligned} t_c=\sup \{t\ge 0:c(t) \in [c_{\textrm{min}},c_{\textrm{max}}]\}, \end{aligned}$$

which signals that *c*(*t*) exits its bounds $[c_{\textrm{min}},c_{\textrm{max}}]$.

### Proposition 6.1

(Control of modulation parameters) Assuming S1, S2 and C1, there exist constants $\delta _{12},C_{12}> 0$ such that the following holds true. For all $\eta \in [0,\eta _0]$, all $C_{12}\sigma ,C_{12}\epsilon \in [0,\eta ]$ and T≥0 the stopping times $\tau ^T_{\textrm{mod}}, \tau ^T_{\textrm{st}}$ and $t_c$ satisfy

$$\begin{aligned}\mathbb {P}\big [\{\tau ^T_{\textrm{mod}}(\eta ) \le \tau ^T_{\textrm{st}}(\eta )\wedge \Delta T\} \cap \{T+\tau _{\textrm{mod}}^T(\eta ) \le t_c \}\big ]\le e^{-\delta _{12}\eta ^2/\sigma ^2}.\end{aligned}$$

We first treat the stopping time $\tau ^{T}_{\textrm{amp,1}}$ by estimating the local amplitude $c^T$ through

6.1 $$\begin{aligned} \big |c(T)-c^T(s)\big |\le&\int _0^s \Big |c_d^{\sigma ,\epsilon ,T}\big ({\textbf{m}}^T(s'),s'\big )\Big | \ {\mathrm d}s'+\sigma \Big |\int _0^s\big \langle c^{T}_s\big ({\textbf{m}}^T(s'),s'\big ),T_{\xi (T)+c(T)s'}{\mathrm d}W_{T+s'}^Q\big \rangle \Big |. \end{aligned}$$

This is obtained straightforwardly from (4.3). We recall that $\eta _0$ below is introduced in Proposition 5.1.

### Lemma 6.2

Assuming S1, S2 and C1, there exist constants $\delta _{13},C_{13}> 0$ such that the following holds true. For all $\eta \in [0,\eta _0]$, all $C_{13}\sigma ,C_{13}\epsilon \in [0,\eta ]$ and T≥0 the stopping times $\tau ^{T}_{\textrm{amp,1}}, \tau ^{T}_{\textrm{pos,1}}, \tau ^T_{\textrm{st}}$ and $\tau ^{T}_c$ satisfy

$$\begin{aligned} \mathbb {P}\big [\tau ^{T}_{\textrm{amp,1}}(\eta ) \le \tau ^{T}_{\textrm{pos,1}}( \eta )\wedge \tau ^T_{\textrm{st}}(\eta )\wedge \tau ^{T}_c\wedge \Delta T\big ]\le e^{-\delta _{13}\eta ^2/\sigma ^2}.\end{aligned}$$

### Proof

Writing $\tau =\min \{\tau ^{T}_{\textrm{amp,1}}({\eta }),\tau ^{T}_{\textrm{pos,1}}({\eta }), \tau ^T_{\textrm{st}}(\eta ), \tau ^{T}_c\}$, we apply Lemma 4.4 to (6.1) and find

$$\begin{aligned} \big |c(T)-c^T(s)\big | \le&C_5 \int _0^s R_w^T(s')\Vert v^T(s')\Vert _{L^2_w}{\mathrm d}s'\\&+C_5 \epsilon \int _0^sf(T+s')\big (1+\Vert v^T(s')\Vert _{L^2_w}\big ){\mathrm d}s' +C_5 \sigma ^2\int _0^s \big (1+\Vert v^T(s')\Vert _{L^2_w}\big ){\mathrm d}s'\\&+\sigma \Big |\int _0^s\big \langle c^{T}_s\big ({\textbf{m}}^T(s'),s'\big ),T_{\xi (T)+c(T)s'}{\mathrm d}W_{T+s'}^Q\big \rangle \Big |, \end{aligned}$$

$\mathbb {P}$-almost surely for $s'\in [0,\tau ]$. We thus have

$$\begin{aligned} \big |c(T)-c^T(\tau )\big | \le&C_5 \Delta T\Big ( \delta _*(2+2\Delta T )\eta ^2+2(\epsilon + \sigma ^2) \Big )+\sigma \sup _{0\le s \le \Delta T} C(s), \end{aligned}$$

where we have abbreviated

$$\begin{aligned}C(s):=\Big |\int _0^s1_{[0,\tau ]}(s')\big \langle c^{T}_s\big ({\textbf{m}}^T(s'),s'\big ),T_{\xi (T)+c(T)s'}{\mathrm d}W_{T+s'}^Q\big \rangle \Big |.\end{aligned}$$

We note via Lemma 5.4 that $\mathbb {P}$-almost surely for $s'\in [0,\tau ]$, the integrand above satisfies

$$\begin{aligned} \Big \Vert \big \langle c^{T}_s\big ({\textbf{m}}^T(s'),s'\big ),T_{\xi (T)+c(T)s'}\cdot \big \rangle \Big \Vert ^2_{\textrm{HS}(L^2_Q,{\mathbb {R}})} \le&C_5^2\big (1+\Vert v^T(s')\Vert _{L^2_w}\big )^2\le C_5^2(1+\eta )^2. \end{aligned}$$

Suppose now that $\tau ^{T}_{\textrm{amp,1}}(\eta )\le \tau ^{T}_{\textrm{pos,1}}(\eta )\wedge \tau ^T_{\textrm{st}}(\eta )\wedge \tau ^T_c$ and that the stopping time $\tau ^{T}_{\textrm{amp,1}}$ is activated because the bound $\delta _*\eta$ is reached on [0,ΔT]. In this case,

$$\begin{aligned} \delta _*\eta =&\big |c(T)-c^T(\tau )\big | \le \delta _*\eta /4+2C_5 \Delta T (\epsilon +\sigma ^2)+\sigma \sup _{0\le s \le \Delta T} C(s), \end{aligned}$$

where we have used that $C_5 \Delta T \delta _*(2+2\Delta T)\eta ^2\le \delta _*\eta /4$ via C1. Ensuring that also $2C_5 \Delta T (\epsilon +\sigma ^2)\le \delta _*\eta /4$ via $C_{13}$, this can only happen if

$$\begin{aligned}\sigma \sup _{0\le s \le \Delta T} C(s)\ge \delta _*\eta /2.\end{aligned}$$

Ensuring furthermore that $\sigma \sqrt{\Delta T}\le \frac{\delta _*\eta }{2eC_5(1+\eta _0)K}$, Theorem 5.3 implies the tail bound

6.2 $$\begin{aligned} \mathbb {P}\Big [\sigma \sup _{0\le s\le \Delta T}C(s)\ge \delta _*/2\Big ]\le e^{-\delta _{13}\eta ^2/\sigma ^2}, \end{aligned}$$

with

$$\begin{aligned}\delta _{13}=\big (2eC_5(1+\eta _0)K\big )^{-2}\delta _*^2/\Delta T.\end{aligned}$$

Hence,

$$\begin{aligned}\mathbb {P}\big [\tau ^{T}_{\textrm{amp,1}}(\eta ) \le \tau ^{T}_{\textrm{pos,1}}( \eta )\wedge \tau ^T_{\textrm{st}}(\eta )\wedge \tau ^{T}_c\wedge \Delta T\big ]\le e^{-\delta _{13}\eta ^2/\sigma ^2 }.\end{aligned}$$

□

Next, we set out to control the stopping time $\tau ^{T}_{\textrm{pos},1}$ via the estimate

6.3 $$\begin{aligned} \big |\xi (T)+c(T)s-\xi ^T(s)\big |\le&\int _0^s \big |c^T(s')-c(T)\big |+\big |\Omega _d^{\sigma ,\epsilon ,T}\big ({\textbf{m}}^T(s'),s'\big )\big |{\mathrm d}s' \nonumber \\&+\sigma \Big |\int _0^s\big \langle \Omega ^{T}_s\big ({\textbf{m}}^T(s'),s'\big ),T_{\xi (T)+c(T)s'}{\mathrm d}W_{T+s'}^Q\big \rangle \Big | \end{aligned}$$

on the local soliton position $\xi ^T$. Note here the dependence on $|c^T(s')-c(T)\big |$, which is under control before time $\tau ^{T}_{\textrm{amp},1}$.

### Lemma 6.3

Assuming S1, S2 and C1, there exist constants $\delta _{14},C_{14}>0$ such that the following holds true. For all $\eta \in [0,\eta _0]$, all $C_{14}\sigma ,C_{14}\epsilon \in [0,\eta ]$ and T≥0 the stopping times $\tau ^{T}_{\textrm{amp,2}}, \tau ^{T}_{\textrm{pos,2}}, t_{\textrm{st}}$ and $t_c$ satisfy

$$\begin{aligned}\mathbb {P}\big [\tau ^{T}_{\textrm{pos,1}}(\eta ) \le \tau ^{T}_{\textrm{amp,1}}(\eta )\wedge \tau ^T_{\textrm{st}}(\eta )\wedge \tau ^{T}_c\wedge \Delta T\big ]\le e^{-\delta _{14}\eta ^2/\sigma ^2}.\end{aligned}$$

### Proof

Let us once more write $\tau =\min \{\tau ^{T}_{\textrm{amp,1}}({\eta }),\tau ^{T}_{\textrm{pos,1}}({\eta }), \tau ^T_{\textrm{st}}(\eta ), \tau ^{T}_c\}$. From (6.3) we obtain the inequality

$$\begin{aligned} 2\Delta T\delta _*\eta =&\big |\xi (T)+c(T)\tau -\xi ^T(\tau )\big | \\ \le&\delta _*\Delta T\eta +C_5 \Delta T \big (\delta _*(2+2\Delta T )\eta ^2+\epsilon (1+\eta ) +\sigma ^2 (1+\eta )\big )\\&+\sigma \sup _{0\le s \le \Delta T} \Big |\int _0^s1_{[0,\tau ]}(s')\big \langle \Omega ^{T}_s\big ({\textbf{m}}^T(s'),s'\big ),T_{\xi (T)+c(T)s'}{\mathrm d}W_{T+s'}^Q\big \rangle \Big |. \end{aligned}$$

Ensuring $C_5 \big (\delta _*(2+2\Delta T )\eta ^2+\epsilon (1+\eta ) +\sigma ^2 (1+\eta )\big )\le \delta _*\eta /2$ as well as $\sigma \le \frac{\sqrt{\Delta T}\delta _*\eta }{2eC_5(1+\eta _0)K}$ via $C_{14}$, the result follows from the tail bound

$$\begin{aligned}\mathbb {P}\Big [\sigma \sup _{0\le s \le \Delta T} \Big |\int _0^s1_{[0,\tau ]}(s')\big \langle \Omega ^{T}_s\big ({\textbf{m}}^T(s'),s'\big ),T_{\xi (T)+c(T)s'}{\mathrm d}W_{T+s'}^Q\big \rangle \Big |\ge \Delta T\eta /2\Big ]\le e^{-\delta _{14} \eta ^2/\sigma ^2},\end{aligned}$$

where

$$\begin{aligned}\delta _{14}=\Delta T\big (2eC_5(1+\eta _0)K\big )^{-2}\delta _*^2,\end{aligned}$$

analogous to (6.2). □

Control of the global amplitude is established by estimating (3.3) as

$$\begin{aligned} \big |c(T)-c(T+s)\big |\le&\int _T^{T+s} \Big |c_d^{\sigma ,\epsilon }\big (v(t'),c(t'),t'\big )\Big | \ {\mathrm d}t'+\sigma \Big |\int _T^{T+s}\big \langle c_s\big (v(t'),c(t')\big ),T_{\xi (t')}{\mathrm d}W_{t'}^Q\big \rangle \Big |. \end{aligned}$$

The difference between the local and global positions is controlled via

$$\begin{aligned}|\xi (T+s)-\xi ^T(s)|\le \big |\xi (T)+c(T)s-\xi ^T(s)\big |+\big |\xi (T)+c(T)s-\xi (T+s)\big |,\end{aligned}$$

where

$$\begin{aligned} \big |\xi (T)+c(T)s-\xi (T+s)\big |\le&\int _{T}^{T+s} |c(T)-c(t')|+\big |\Omega _d^{\sigma ,\epsilon }(v(t'),c(t'),t'\big )\big | {\mathrm d}t'\\&+\sigma \Big |\int _{T}^{T+s}\langle \Omega _s(v(t'),c(t')\big ),T_{\xi (t')} {\mathrm d}W_{t'}^Q\rangle \Big |. \end{aligned}$$

This leads to the following estimates on $\tau ^T_{\textrm{amp},2}$ and $\tau ^T_{\textrm{pos},2}$.

### Lemma 6.4

Assuming S1, S2 and C1, there exist constants $\delta _{15},C_{15}> 0$ such that the following holds true. For all $\eta \in [0,\eta _0]$, all $C_{15}\sigma ,C_{15}\epsilon \in [0,\eta ]$ and T≥0 the stopping times $\tau ^{T}_{\textrm{amp,2}},\tau ^{T}_{\textrm{pos,2}}, t_{\textrm{st}}$ and $t_c$ satisfy

$$\begin{aligned}\mathbb {P}\big [\{\tau ^{T}_{\textrm{amp,2}}({\eta }) \le \tau ^{T}_{\textrm{pos,2}}(\eta )\wedge \Delta T\}\cap \{T+\tau ^{T}_{\textrm{amp,2}}({\eta })\le t_{\textrm{st}}(2\eta )\wedge t_c\}\big ]\le e^{-\delta _{15}\eta ^2/\sigma ^2},\end{aligned}$$

and

$$\begin{aligned}\mathbb {P}\big [\{ \tau ^{T}_{\textrm{pos,2}}(\eta )\le \tau ^{T}_{\textrm{amp,2}}({\eta })\wedge \Delta T\}\cap \{T+\tau ^{T}_{\textrm{pos,2}}(\eta )\le t_{\textrm{st}}(2\eta )\wedge t_c\}\big ]\le e^{-\delta _{15}\eta ^2/\sigma ^2}.\end{aligned}$$

### Proof

These bounds follow from computations fully analogous to those in the proofs of Lemma 6.2 and Lemma 6.3. □

As a last preparation, we show how the correspondence between the local perturbation v(T+s) and global perturbation $v^T(s)$ manifests in the stopping times $\tau _{\textrm{st}}^T$ and its global counterpart $t_{\textrm{st}}$. We recall that

$$\begin{aligned}t_{\textrm{st}}(\eta )=\sup \big \{t\ge 0:\Vert v(t)\Vert _{H^1_w} \le \eta \big \}.\end{aligned}$$

### Lemma 6.5

Assume S1, S2 and C1. For each T≥0 and $\eta \in [0,\eta _0]$, the stopping times $\tau ^T_{\textrm{st}}, \tau ^{T}_{\textrm{mod}}, t_c$ and $t_\textrm{st}$ satisfy

$$\begin{aligned}\min \{T+\tau ^T_{\textrm{st}}(\eta ),T+\tau ^{T}_{\textrm{mod}}(\delta _*\eta ),t_c\}\le t_\textrm{st}(2\eta ),\end{aligned}$$

$$\mathbb {P}$$-almost surely.

### Proof

Writing $\tau =\min \{\tau ^T_{\textrm{st}}(\eta ),\tau ^{T}_{\textrm{mod}}(\eta ),t_c-T\}$, we have

$$\begin{aligned}c(T+s)\in [c_{\textrm{min}}, c_{\textrm{max}}], \quad {\text {and}}\quad \big |c^{ T}(s)-c(T+s)\big |\le 2\delta _*\eta , \quad {\text {for}}\quad s\in [0,\tau ].\end{aligned}$$

Since $\delta _*\le \min \{\frac{1}{4}c_{\textrm{min}},\frac{1}{2}c_{\textrm{max}}\}$ via C1, it follows that also

$$\begin{aligned}c^{T}(s)\in [\tfrac{1}{2}c_{\textrm{min}}, 2c_{\textrm{max}}],\quad s\in [0,\tau ].\end{aligned}$$

Lemma 4.2 now gives

$$\begin{aligned}\Big |\big \Vert v({ T}+s)\big \Vert _{H^1_w}-e^{w(\xi ( T)+c(T)s-\xi (T+s))}\big \Vert v^{ T}(s)\big \Vert _{H^1_w}\Big |\le 3C_4\delta _*\eta .\end{aligned}$$

We then obtain the bound

$$\begin{aligned}\Vert v(T+s)\Vert _{H^1_w}\le e^{w(\xi ( T)+c(T)s-\xi (T+s))}\Vert v^T(s)\Vert _{H^1_w}+3C_4\eta \le e^{2\eta _0}\delta _*\eta +3C_4\delta _*\eta <2\eta ,\end{aligned}$$

for all $s\in [0,\tau ]$, where we have used that $\delta _*<(e^2+3C_4)^{-1}$ via C1. □

We are then ready to collect our results and establish control of $\tau _{\textrm{mod}}^T$. We note that the event

$$\begin{aligned}\tau ^T_{\textrm{mod}}(\eta ) \le \tau ^T_{\textrm{st}}(\eta )\wedge \Delta T\quad \text {while}\quad T+\tau _{\textrm{mod}}^T(\eta ) \le t_c\end{aligned}$$

implies that one of the events

$$\begin{aligned} {\mathcal {B}}_1=&\big \{\tau ^{T}_{\textrm{amp,1}}(\eta ) \le \tau ^{T}_{\textrm{pos,1}}( \eta )\wedge \tau ^{T}_{\textrm{amp,2}}({\eta })\wedge \tau ^T_{\textrm{st}}(\eta )\wedge \Delta T\big \}\cap \big \{T+\tau ^{T}_{\textrm{amp,1}}(\eta ) \le t_c\big \};\\ {\mathcal {B}}_2=&\big \{\tau ^{T}_{\textrm{pos,1}}( \eta ) \le \tau ^{T}_{\textrm{amp,1}}(\eta )\wedge \tau ^{T}_{\textrm{amp,2}}({\eta })\wedge \tau ^T_{\textrm{st}}(\eta )\wedge \Delta T\big \}\cap \big \{ T+ \tau ^{T}_{\textrm{pos,1}}( \eta ) \le t_c\big \};\\ {\mathcal {B}}_3=&\big \{\tau ^{T}_{\textrm{amp,2}}({\eta }) \le \tau ^{T}_{\textrm{mod}}(\eta )\wedge \tau ^T_{\textrm{st}}(\eta ) \wedge \Delta T\big \}\cap \big \{ T+\tau ^{T}_{\textrm{amp,2}}({\eta })\le t_c\big \}; \\ {\mathcal {B}}_4=&\big \{\tau ^{T}_{\textrm{pos,2}}( \eta )\le \tau ^{T}_{\textrm{mod}}({\eta })\wedge \tau ^T_{\textrm{st}}(\eta )\wedge \Delta T\big \}\cap \big \{ T+\tau ^{T}_{\textrm{pos,2}}( \eta )\le t_c\big \}, \end{aligned}$$

holds, depending on which of $\tau ^T_{\textrm{amp},1},\tau ^T_{\textrm{amp},2},\tau ^T_{\textrm{pos},1}$ and $\tau ^T_{\textrm{pos},2}$ is smallest.

### Proof of Proposition 6.1

We bound the probability of the events ${\mathcal {B}}_1, {\mathcal {B}}_2, {\mathcal {B}}_3$ and ${\mathcal {B}}_4$. In case ${\mathcal {B}}_1$ or ${\mathcal {B}}_2$ holds, note that $t_c-T\wedge \tau ^T_{\textrm{amp},2}(\eta ) \le \tau ^T_c$. Indeed,

$$\begin{aligned} |c^T(s)-c(T+s)|\le \eta _0 \quad \text {and} \quad c(T+s)\in [c_{\textrm{min}},c_{\textrm{max}}] \end{aligned}$$

implies that $c^T(s)\in [\tfrac{1}{2}c_{\textrm{min}},2c_{\textrm{max}}]$. Thus, the probability of ${\mathcal {B}}_1\cup {\mathcal {B}}_2$ is controlled by Lemma 6.2 and Lemma 6.3, respectively.

In case ${\mathcal {B}}_3$ holds, Lemma 6.5 implies $T+\tau ^{T}_{\textrm{amp,2}}({\eta })\le t_{\textrm{st}}(2\eta )$. Similarly, the event ${\mathcal {B}}_4$ implies $T+\tau ^{T}_{\textrm{pos,2}}({\eta })\le t_{\textrm{st}}(2\eta )$. Hence, the probability of ${\mathcal {B}}_3\cup {\mathcal {B}}_4$ is bounded via Lemma 6.4. □

## Global control

In this section, we establish long-time control of the global modulation system $\big (v(t),c(t),\xi (t)\big )$ introduced in Section 3. Our first result controls the probability that the soliton amplitude *c*(*t*) exits its bounds $[c_{\textrm{min}},c_{\textrm{max}}]$. Secondly, we analyze the growth of the remainder *v*(*t*) in the unweighted space $L^2$. Besides being interesting in its own right, this is a crucial ingredient toward controlling the weighted norm of *v* via Proposition 5.1. Since the traveling-wave operator ${\mathcal {L}}_c$ is not exponentially stable on $L^2$, it is standard to analyze the unweighted norm via energy arguments [25, 28].

Henceforth, we assume an integrability condition on the forcing term *f*, limiting the total energy contribution of the deterministic forcing in (1.1).

**C2**: *The constant *E>0 *satisfies*
  $$\begin{aligned}c_{\textrm{min}}\le c_*e^{-3E} \quad \text {and} \quad c_{\textrm{max}}\ge c_*e^{3E}.\end{aligned}$$
  *The forcing term f lies in *$L^1\big ([0,\infty )\big )$* and the constant *$\epsilon _0 \in (0,\infty ]$* is small enough to ensure*
  $$\begin{aligned}\epsilon _0\int _0^\infty |f(t)|{\mathrm d}t\le E.\end{aligned}$$

With this condition in place, the soliton amplitude *c*(*t*) stays within the bounds $[c_{\textrm{min}},c_{\textrm{max}}]$ for times that are small with respect to $\sigma ^2$. Recall the notation

$$\begin{aligned}t_{c}=&\sup \big \{t\ge 0: c(t)\in [c_{\textrm{min}},c_{\textrm{max}}] \big \},\\ t_{\textrm{st}}(\eta )=&\sup \big \{t\ge 0:\Vert v(t)\Vert _{H_w^1}\le \eta \big \}, \end{aligned}$$

the global counterparts of $\tau ^T_c$ and $\tau ^T_{\textrm{st}}(\eta )$ from Section 5. The constant $\delta _{17}$ below will be introduced in Lemma 7.3.

### Proposition 7.1

Assume S1, S2, C1 and C2. For each $\eta \in [0,\eta _0]$, each $\epsilon \in [0,\epsilon _0]$, and $\sigma , T\ge 0$ that satisfy $\sigma ^2 T, \eta ^2 T\le \delta _{17}$ we have

$$\begin{aligned}\mathbb {P}[t_c \le T\wedge t_{\textrm{st}}(\eta )]\le e^{-\delta _{17}/(\sigma ^2 T)}.\end{aligned}$$

The second main result of this section concerns the stopping time

$$\begin{aligned}t_{\textrm{en}}(\eta )=\sup \big \{t\ge 0:\big \Vert v(t)\big \Vert _{L^2}\le \eta \big \},\end{aligned}$$

a global counterpart of $\tau ^T_{\textrm{en}}$ of Section 5. The probability that $\Vert v(t)\Vert _{L^2}$ remains small on an interval [0, *T*] is controlled by the forcing parameters σ and ϵ.

### Proposition 7.2

Assume S1, S2, C1 and C2. There exist constants $C_{16}, \delta _{16}>0$ such that for all $\sigma ,T,\lambda >0$ satisfying $\sigma ^2 T \le \delta _{16}$, each $\eta \in [0,\eta _0]$ and each $\epsilon \in [0,\epsilon _0]$, we have

$$\begin{aligned}\mathbb {P}\Big [t_{\textrm{en}}(\lambda )\le T\wedge t_c \wedge t_{\textrm{st}}(\eta )\Big ]\le C_{16}\lambda ^{-1}\Big (T\big ( \sigma ^2+\epsilon \eta +\eta ^2\big )+\sqrt{T}\sigma \eta \Big ).\end{aligned}$$

We first establish Proposition 7.1, making use of the evolution equation

7.1 $$\begin{aligned}&{\mathrm d}\log (c(t))=\Big (\frac{c_d^{\sigma ,\epsilon }(v(t),c(t),t)}{c(t)}-\sigma ^2\frac{\big \Vert Q^{1/2}c_s(v(t),c(t))\big \Vert _{L^2}^2}{2c^2(t)} \ \Big ) \ \nonumber \\&\quad {\mathrm d}t+\sigma \frac{\langle c_s(v(t),c(t)),T_{\xi }{\mathrm d}W_t^Q\rangle }{c(t)}, \end{aligned}$$

which one finds by applying the Itô lemma [7, Theorem 4.32] to (3.3). In particular, as an intermediate result, we control the stopping time

$$\begin{aligned} t_{\textrm{log}}=\sup \{t\ge 0 :|\log (c(t)/c_*)-\frac{4}{3}\epsilon \int _0^t|f(t')|{\mathrm d}t'|\le E\}. \end{aligned}$$

It is then ensured that *c*(*t*) remains within the limits $[c_{\textrm{min}},c_{\textrm{max}}]$ before $t_{\log }$, and consequently $t_c\ge t_{\log }$.

### Lemma 7.3

Assuming S1, S2, C1 and C2, there exists a constant $\delta _{17}>0$ such that the following holds true. For each $\eta \in [0,\eta _0]$, each $\epsilon \in [0,\epsilon _0]$, and $\sigma , T\ge 0$ satisfying $\sigma ^2 T, \eta ^2 T\le \delta _{17}$ we have

$$\begin{aligned} \mathbb {P}[t_{\textrm{log}}\le t_{\textrm{st}}(\eta )\wedge T]\le e^{-\delta _{17}/\sigma ^2T}. \end{aligned}$$

### Proof

Using the identity $c^{-1}c_f(0,c)=\frac{4}{3}$, we estimate (7.1) as

$$\begin{aligned}&\Big |\log (c(t)/c_*)-\frac{4}{3}\epsilon \int _0^t|f(t')|{\mathrm d}t'\Big |\le \int _0^t\Big |\frac{c_d^0(v(t'),c(t'))}{c(t')}\Big |\\&\quad +\epsilon |f(t')|\Big |\frac{c_f(v(t'),c(t'))-c_f(0,c(t'))}{c(t')}\Big | \ {\mathrm d}t'\\&\quad +\sigma ^2\int _0^t\Big |\frac{c_d(v(t'),c(t'))}{c(t')}\Big |-\frac{\big \Vert Q^{1/2}c_s(v(t'),c(t'))\big \Vert _{L^2}^2}{2c^2(t')} \ {\mathrm d}t'\\&\quad +\sigma \Big |\int _0^t\frac{\langle c_s(v(t'),c(t')),T_{\xi }{\mathrm d}W_{t'}^Q\rangle }{c(t')}\Big |\\&\quad \le C_2t (\eta ^2+\sigma ^2)+C_2\epsilon \eta \int _0^t |f(t')|{\mathrm d}t'\\&\quad +\sigma \Big |\int _0^t\frac{\langle c_s(v(t'),c(t')),T_{\xi }{\mathrm d}W_{t'}^Q\rangle }{c(t')}\Big |, \end{aligned}$$

for $t\in [0,t_{\log }\wedge t_{\textrm{st}}(\eta )]$. Condition C2 now gives

$$\begin{aligned}\epsilon \eta \int _0^t |f(t')|{\mathrm d}t'\le E\eta .\end{aligned}$$

Ensuring $C_2T (\eta ^2+\sigma ^2)+C_2E \eta \le L/2,$ the inequality $t_{\textrm{log}}\le t_{\textrm{st}}(\eta )\wedge T$ implies

$$\begin{aligned} E=&\Big |\log (c(t_{\textrm{log}})/c_*)-\frac{4}{3}\int _0^{t_{\textrm{log}}}|f(t')|{\mathrm d}t'\Big |\\ \le&E/2+\sigma \sup _{0\le t \le T}\Big |\int _0^t 1_{[0,t_{\textrm{log}}\wedge t_{\textrm{st}}(\eta )]}(t')\frac{\langle c_s(v(t'),c(t')),T_{\xi }{\mathrm d}W_{t'}^Q\rangle }{c(t')}\Big | \end{aligned}$$

and consequently

$$\begin{aligned} \sigma \sup _{0\le t \le T}\Big |\int _0^t 1_{[0,t_{\textrm{log}}\wedge t_{\textrm{st}}(\eta )]}(t')\frac{\langle c_s(v(t'),c(t')),T_{\xi }{\mathrm d}W_{t'}^Q\rangle }{c(t')}\Big |\ge E/2. \end{aligned}$$

The probability of this event is bounded by the tail estimate Theorem 5.3. □

### Proof of Proposition 7.1

The result now follows as an immediate corollary to Lemma 7.3, in view of the fact that $t_c\ge t_{\log }$. □

We now turn our attention to the energy result, Proposition 7.2. Our first result regarding the global modulation system outlined in Section 3 is as follows.

### Lemma 7.4

Assume S1 and S2. For each t≥0, the inequalities

$$\begin{aligned} \Vert v(t')\Vert _{L^2_w}\le \delta _1 \quad \text {and} \quad c(t')\in [c_{\textrm{min}},c_{\textrm{max}}],\quad t' \in [0,t] \end{aligned}$$

imply that

7.2 $$\begin{aligned} \big \Vert v(t)\big \Vert _{L^2}^2=&\sigma ^2\int _0^t\Big (6c^{3/2}- 9 c_d(v,c) c^{1/2} - \tfrac{9}{4} \big \Vert Q^{1/2}c_s(v,c)\big \Vert _{L^2}^2 c^{-1/2}+\big \Vert v\big \Vert _{L^2}^2\Big ) {\mathrm d}t'\nonumber \\&- 9\int _0^t c^0_d(v,c) c^{1/2} {\mathrm d}t' +\epsilon \int _0^tf(t')\Big (2\Vert v\Vert _{L^2}^2-9\big (c_f(v,c)-c_f(0,c)\big )\Big ){\mathrm d}t'\nonumber \\&-9\sigma \int _0^t c^{1/2}\big \langle c_s(v,c)-c_s(0,c),{\mathrm d}W_{t'}^Q\big \rangle +2\sigma \int _0^t\big \langle 2\phi _cv+v^2,T_{\xi } {\mathrm d}W_{t'}^Q\big \rangle \end{aligned}$$

holds $\mathbb {P}$-almost surely.

### Proof

We observe that

7.3 $$\begin{aligned} \big \Vert v(t)\big \Vert _{L^2}^2=\big \Vert u(t)\big \Vert _{L^2}^2-\big \Vert \phi _{c(t)}\big \Vert _{L^2}^2\end{aligned}$$

by Pythagoras’ theorem and the orthogonality condition (3.2). Via Itô’s lemma [6, Theorem 1] applied to the functional $M(u)=\Vert u\Vert _{L^2}^2$, we obtain

7.4 $$\begin{aligned} {\mathrm d}\Vert u\Vert _{L^2}^2=&\sigma ^2 \Vert u\Vert ^2_{L^2}\ {\mathrm d}t+2\epsilon f(t)\Vert u\Vert _{L^2}^2\ {\mathrm d}t+2 \sigma \langle u,u \ {\mathrm d}W_t^Q\rangle \nonumber \\ =&\big (\sigma ^2 +2\epsilon f(t)\big )\big (6c^{3/2}(t)+\Vert v\Vert _{L^2}^2\big )\ {\mathrm d}t+2 \sigma \big \langle \phi _c^2+2\phi _c v+v^2, \ T_{\xi } {\mathrm d}W_t^Q\big \rangle .\end{aligned}$$

Here we have used that $M:L^2\rightarrow {\mathbb {R}}$ is twice differentiable with Fréchet derivatives

$$\begin{aligned} {\mathrm d}M(u)[v]=2\langle u,v\rangle , \quad {\mathrm d}^2 M(u)[v,w]=2\langle v,w\rangle , \end{aligned}$$

and that the Airy group $\{e^{-\partial _x^3t}\}_{t\in {\mathbb {R}}}$ is a $C_0$-group of isometries on $L^2$, in the sense that

$$\begin{aligned}&M(e^{-\partial _x^3t}u)=\Vert u\Vert _{L^2}^2, \quad {\mathrm d}M(e^{-\partial _x^3t}u)[e^{-\partial _x^3t}v]=2\langle u,v\rangle , \\&\quad {\mathrm d}^2 M(e^{-\partial _x^3t}u)[e^{-\partial _x^3t}v,e^{-\partial _x^3t}w]=2\langle v,w\rangle . \end{aligned}$$

On the other hand, we can also employ Itô’s lemma to compute

7.5 $$\begin{aligned} {\mathrm d}\Vert \phi _{c(t)}\Vert _{L^2}^2={\mathrm d}\big (6c^{3/2}(t)\big ) =&\Big ( 9c_d^{\sigma ,\epsilon }(v,c,t) c^{1/2} + \tfrac{9}{4}\sigma ^2 \big \Vert Q^{1/2}c_s(v,c)\big \Vert _{L^2}^2 c^{-1/2} \Big )\ {\mathrm d}t \end{aligned}$$

7.6 $$\begin{aligned}&+ 9\sigma c^{1/2} \big \langle c_s(v,c),T_{\xi } {\mathrm d}W^Q_t\big \rangle , \end{aligned}$$

where we recall that

$$\begin{aligned}c_d^{\sigma ,\epsilon }(v,c,t)=c_d^0(v,c)+\epsilon f(t)c_f(v,c)+\sigma ^2 c_d(v,c).\end{aligned}$$

Note here that $9 c^{1/2} c_s(0,c)=2\phi _c^2$, thus the leading-order diffusion term in ${\mathrm d}\Vert u\Vert _{L^2}^2$ equals that in ${\mathrm d}\Vert \phi _{c(t)}\Vert _{L^2}^2$. Similarly,

$$\begin{aligned}c_d^{\sigma ,\epsilon }(0,c,t)=\tfrac{4}{3}\epsilon f(t)c+\sigma ^2 c_d(0,c)\end{aligned}$$

shows that the leading-order ϵ-dependent term in ${\mathrm d}\Vert u\Vert _{L^2}^2$ equals that in ${\mathrm d}\Vert \phi _{c(t)}\Vert _{L^2}^2$. The result now follows by subtracting (7.5) from (7.4). □

Control on the stochastic integrals in (7.2) is provided in the following lemma.

### Lemma 7.5

Assuming S1 and S2, there exists a constant $C_{18}>0$ such that for all ${\tilde{v}}\in L^2_w$ satisfying $\Vert {\tilde{v}}\Vert _{L^2_w}\le \delta _2$, all ${\tilde{\xi }}\in {\mathbb {R}}$ and ${\tilde{c}}\in [c_{\textrm{min}},c_{\textrm{max}}]$ we have the inequalities

$$\begin{aligned} \big \Vert \langle 2\phi _{{\tilde{c}}}{\tilde{v}}+{\tilde{v}}^2,T_{{\tilde{\xi }}}\cdot \rangle \big \Vert _{\textrm{HS}(L_Q^2,{\mathbb {R}})} \le C_{18} \Big (\Vert {\tilde{v}}\Vert _{L_w^2}+\Vert {\tilde{v}}\Vert ^2_{L^2}\Big ) \end{aligned}$$

and

$$\begin{aligned} \big \Vert \langle c_s({\tilde{v}},{{\tilde{c}}})-c_s(0,{{\tilde{c}}}),T_{{\tilde{\xi }}}\cdot \rangle \big \Vert _{\textrm{HS}(L_Q^2,{\mathbb {R}})} \le C_{18}\Vert {\tilde{v}}\Vert _{L_w^2}. \end{aligned}$$

### Proof

Applying Lemma 5.4, we have

$$\begin{aligned} \big \Vert \langle 2\phi _{{\tilde{c}}}{\tilde{v}}+{\tilde{v}}^2,T_{{\tilde{\xi }}}\cdot \rangle \big \Vert _{\textrm{HS}(L_Q^2,{\mathbb {R}})} \le&2\Vert Q^{1/2}(\phi _{{\tilde{c}}}{\tilde{v}})\Vert _{L^2}+C_{10}\Vert {\tilde{v}}^2\Vert _{L^1}\\ \le&\Vert q\Vert ^{1/2}_{L^1} \Vert \phi _{{\tilde{c}}}\Vert _{L^2_{-w}}\Vert {\tilde{v}}\Vert _{L^2_w}+C_{10}\Vert {\tilde{v}}\Vert _{L^2}^2, \end{aligned}$$

and

$$\begin{aligned} \big \Vert \langle c_s({\tilde{v}},{{\tilde{c}}})-c_s(0,{{\tilde{c}}}),T_{{\tilde{\xi }}}\cdot \rangle \big \Vert _{\textrm{HS}(L_Q^2,{\mathbb {R}})}= \big \Vert Q^{1/2}c_s({\tilde{v}},{{\tilde{c}}})-Q^{1/2}c_s(0,{{\tilde{c}}})\big \Vert _{L^2}. \end{aligned}$$

The result follows by applying Lemma 3.3. □

### Proof of Proposition 7.2

Using (7.2), we may $\mathbb {P}$-almost surely estimate

$$\begin{aligned} \Vert v(t)\Vert _{L^2}^2\le&C_2\int _0^t \big (\sigma ^2+2\epsilon |f(t')|\big )\Vert v(t')\Vert _{L^2}^{2}{\mathrm d}t'+C_2 t \left( \sigma ^2+\epsilon \eta + \eta ^2 \right) \\&9\sigma \Big |\int _0^t \big \langle c_s(v,c)-c_s(0,c),{\mathrm d}W_{t'}^Q\big \rangle \Big |+2\sigma \Big |\int _0^t\big \langle 2\phi _cv+v^2,T_{\xi } {\mathrm d}W_{t'}^Q\big \rangle \Big |, \end{aligned}$$

for $t\in [0,t_c\wedge t_{\textrm{st}}(\eta )]$, where we have controlled the modulation parameters in the deterministic integrals via Lemma 3.3. After taking a supremum and expectations,

7.7 $$\begin{aligned} \mathbb {E}\sup _{0\le t' \le T\wedge t_{c}\wedge t_{\textrm{st}}(\eta )}\Vert v(t')\Vert _{L^2}^2\le&C_2\int _0^T \big (\sigma ^2+2\epsilon |f(t)|\big )\mathbb {E}\sup _{0\le t'\le t\wedge t_{c}\wedge t_{\textrm{st}}(\eta )}\Vert v(t')\Vert _{L^2}^{2}{\mathrm d}t\nonumber \\&+C_2 T \left( \sigma ^2+\epsilon \eta + \eta ^2 \right) +\sigma I(T),\end{aligned}$$

where we have abbreviated

$$\begin{aligned} I(T)=&9 \mathbb {E}\sup _{0\le t \le T\wedge t_{c}\wedge t_{\textrm{st}}(\eta )}\Big |\int _0^t \big \langle c_s(v,c)-c_s(0,c),{\mathrm d}W_{t'}^Q\big \rangle \Big |\\&+2 \mathbb {E}\sup _{0\le t \le T\wedge t_{c}\wedge t_{\textrm{st}}(\eta )}\Big |\int _0^t\big \langle 2\phi _cv+v^2,T_{\xi } {\mathrm d}W_{t'}^Q\big \rangle \Big |. \end{aligned}$$

The Burkholder-Davis-Gundy inequality [26, proposition 2.1] together with Lemma 7.5 yields the control

$$\begin{aligned} I(T) \le&C_{18} \mathbb {E}\Big [\Big (\int _0^{T\wedge t_{c}\wedge t_{\textrm{st}}(\eta )} \Vert v(t)\Vert ^2_{L^2_w}+ \Vert v(t)\Vert _{L^2}^4{\mathrm d}t\Big )^{1/2}\Big ]\\ \le&C_{18}\eta \sqrt{T}+C_{18}\sqrt{T} \mathbb {E}\sup _{0\le t \le T\wedge t_{c}\wedge t_{\textrm{st}}(\eta )} \Vert v(t)\Vert _{L^2}^2. \end{aligned}$$

If $\delta _{16}$ is small enough to ensure $C_{18}\sigma \sqrt{T}\le 1/2$, we may bring this last term to the left in (7.7):

$$\begin{aligned} \tfrac{1}{2}\mathbb {E}\sup _{0\le t \le T\wedge t_{c}\wedge t_{\textrm{st}}(\eta )}\Vert v(t)\Vert _{L^2}^2\le&C_2\int _0^T \big (\sigma ^2+2\epsilon |f(t)|\big )\mathbb {E}\sup _{0\le t'\le t\wedge t_{c}\wedge t_{\textrm{st}}(\eta )}\Vert v(t')\Vert _{L^2}^{2}{\mathrm d}t\\&+C_2 T \left( \sigma ^2+\epsilon \eta + \eta ^2 \right) +C_2\sigma \sqrt{T}\eta .\end{aligned}$$

Grönwall’s inequality then yields

$$\begin{aligned} \mathbb {E}\sup _{0\le t \le T\wedge t_{c}\wedge t_{\textrm{st}}(\eta )}\Vert v(t)\Vert _{L^2}^2\le&2C_2e^{C_2\sigma ^2 T}e^{2C_2\epsilon \int _0^T|f(t)|{\mathrm d}t}\Big (T \left( \sigma ^2+\epsilon \eta + \eta ^2 \right) +\sigma \sqrt{T}\eta \Big ). \end{aligned}$$

Noting that C2 implies

$$\begin{aligned}\epsilon \int _0^T|f(t)|{\mathrm d}t\le E,\end{aligned}$$

the result now follows via Markov’s inequality:

$$\begin{aligned} \mathbb {P}\Big [\sup _{0\le t \le T\wedge t_{c}\wedge t_{\textrm{st}}(\eta )}\Vert v(t)\Vert _{L^2}^2\ge \lambda \Big ] \le&2C_2e^{C_2\delta _{16}+2C_2E}\lambda ^{-1}\Big (T\big ( \sigma ^2+\epsilon \eta +\eta ^2\big )+\sigma \sqrt{T} \eta \Big ). \end{aligned}$$

□

## Nonlinear stability

In this section, we collect our results and prove the stability result Theorem 1.1. Our goal here is to show that the event $t_{\textrm{st}}(\eta )\ge T$ occurs with high probability, by ensuring that

- the unweighted $L^2$-norm of *v*(*t*) remains under control on [0, *T*] (Proposition 7.2);
- the difference between the local and global modulation parameters remains under control on [0, *T*] (Proposition 6.1);
- the weighted norm of *v*(*t*) remains under control on [0, *T*] (Proposition 5.1).

Although our primary interest lies in the latter, our proof requires all of the above to hold. Since the stability results of Section 5 and Section 6 hold on intervals of length ΔT, we partition the interval [0, *T*] into

$$\begin{aligned}{[}0,T]\subseteq \bigcup _{n=0}^{\lceil T/\Delta T\rceil -1}[n\Delta T,(n+1)\Delta T),\end{aligned}$$

and seek to establish stability on each intermediate interval $[n\Delta T,(n+1)\Delta T)$. Consider, therefore, for each $n\in {\mathbb {N}}_0$ the stability event ${\mathcal {S}}_n(\eta )\subseteq \Omega$, defined as the set of $\omega \in \Omega$ for which:

(i) the local stopping times at nΔT satisfy
  $$\begin{aligned}\tau ^{n\Delta T}_{\textrm{mod}}(\eta ),\tau ^{n\Delta T}_{\textrm{en}}(\delta _*),\tau ^{n\Delta T}_{\textrm{st}}(\eta )\ge \Delta T;\end{aligned}$$
(ii) at the end point (n+1)ΔT we have
  $$\begin{aligned} \Vert v^{n\Delta T}(\Delta T)\Vert _{H^1_w}\le \tfrac{\eta }{9M},\quad \Vert v((n+1)\Delta T)\Vert _{H^1_w}\le \tfrac{\eta }{3M}. \end{aligned}$$

The following result allows us to establish high probability of the local stability events in a recursive manner: we bound the probability that ${\mathcal {S}}_{n}(\eta )$ fails to hold, provided ${\mathcal {S}}_{n-1}(\eta )$ occurred. We do so under the condition that the global modulation system is under control. In particular, for each $n\in {\mathbb {N}}$, we define the stability event ${\mathcal {G}}_n(\eta )$ as the set of $\omega \in \Omega$ for which the global stopping times $t_c$ and $t_{\textrm{en}}$ reach

8.1 $$\begin{aligned} \quad t_c, t_{\textrm{en}}(\delta _*/2)\ge n\Delta T+ \min \{\tau ^{n\Delta T}_{\textrm{mod}}(\eta ),\tau ^{n\Delta T}_{\textrm{en}}(\delta _*),\tau ^{n\Delta T}_{\textrm{st}}(\eta )\} .\end{aligned}$$

Below, $S_{n}^c$ denotes the complement $\Omega \setminus S_{n}$.

### Proposition 8.1

Assuming S1, S2 and C1, there exist constants $C_{19}, \delta _{19}>0$, such that for each $n\in {\mathbb {N}}$ and each $\eta \in [0,\eta _0]$ the stability events ${\mathcal {S}}_{n-1},{\mathcal {S}}_{n}(\eta )$ and ${\mathcal {G}}_n(\eta )$ satisfy

$$\begin{aligned}\mathbb {P}\Big [{\mathcal {S}}_{n-1}(\eta )\cap {\mathcal {G}}_n(\eta )\cap {\mathcal {S}}^c_{n}(\eta )\Big ]\le C_{19}e^{-\delta _{19}\eta ^2/\sigma ^2}.\end{aligned}$$

### Proof

Let us write

$$\begin{aligned}{\bar{t}}=t_c\wedge t_{\textrm{en}}(\delta _*/2)\quad \text {and} \quad \tau =\tau ^{n\Delta T}_{\textrm{mod}}(\eta )\wedge \tau ^{n\Delta T}_{\textrm{en}}(\delta _*)\wedge \tau ^{n\Delta T}_{\textrm{st}}(\eta ).\end{aligned}$$

Assuming ${\mathcal {S}}_{n-1}$, we distinguish three scenarios through which condition (i) in ${\mathcal {S}}_{n}$ can fail to hold:

$$\begin{aligned} {\mathcal {A}}_1=&\big \{\tau ^{n\Delta T}_{\textrm{mod}}(\eta )<\Delta T\big \}\cap \{n\Delta T+\tau ^{n\Delta T}_{\textrm{mod}}(\eta )\le {\bar{t}}\}\cap \{\tau ^{n\Delta T}_{\textrm{mod}}(\eta )\le \tau \};\\ {\mathcal {A}}_2=&\big \{\tau ^{n\Delta T}_{\textrm{en}}(\delta _*)<\Delta T\big \}\cap \{n\Delta T+\tau ^{n\Delta T}_{\textrm{en}}(\delta _*)\le {\bar{t}}\}\cap \{\tau ^{n\Delta T}_{\textrm{en}}(\delta _*)\le \tau \};\\ {\mathcal {A}}_3=&\big \{\tau ^{n\Delta T}_{\textrm{st}}(\eta )<\Delta T\big \}\cap \{n\Delta T+\tau ^{n\Delta T}_{\textrm{st}}(\eta )\le {\bar{t}}\}\cap \{\tau ^{n\Delta T}_{\textrm{st}}(\eta )\le \tau \}. \end{aligned}$$

The events ${\mathcal {A}}_1,{\mathcal {A}}_2$ and ${\mathcal {A}}_3$ categorize which of the stopping times was first activated before time (n+1)ΔT. Thus, the event that item (i) of $S_n$ fails to hold coincides with

$$\begin{aligned}{\mathcal {A}}={\mathcal {A}}_1\cup {\mathcal {A}}_2\cup {\mathcal {A}}_3.\end{aligned}$$

We proceed by estimating the probabilities of the events ${\mathcal {A}}_1,{\mathcal {A}}_2$ and ${\mathcal {A}}_3$.

1. The event ${\mathcal {A}}_1$ while also ${\mathcal {S}}_{n-1}(\eta )$ implies
  $$\begin{aligned}\tau ^{n\Delta T}_{\textrm{mod}}(\eta ) \le \tau ^{n\Delta T}_{\textrm{st}}(\eta )\wedge \Delta T \quad \text {and}\quad n\Delta T+\tau _{\textrm{mod}}^T(\eta ) \le t_c.\end{aligned}$$
  Proposition 6.1 then gives
  $$\begin{aligned}\mathbb {P}\Big [ {\mathcal {S}}_{n-1}(\eta )\cap {\mathcal {G}}_n(\eta )\cap {\mathcal {A}}_1\Big ]\le e^{-\delta _{12}\eta ^2/\sigma ^2}.\end{aligned}$$
2. The event ${\mathcal {A}}_2$ while also ${\mathcal {S}}_{n-1}(\eta )$ implies
  8.2 $$\begin{aligned} 0<\tau ^{n\Delta T}_{\textrm{en}}(\delta _*)\le \tau ^{n\Delta T}_{\textrm{mod}}(\eta ) \quad \text {and} \quad n\Delta T+\tau ^{n\Delta T}_{\textrm{en}}(\delta _*)\le t_{\textrm{en}}(\delta _*/2)\wedge t_c.\end{aligned}$$
  This event has probability zero. Indeed, we have
  $$\begin{aligned}c^T(s),c(T+s)\in [\tfrac{1}{2}c_{\textrm{min}},2c_{\textrm{max}}],\quad \text {for}\quad s\in [0,\tau _{\textrm{mod}}^{n\Delta T}(\eta )\wedge (t_c-n\Delta T)].\end{aligned}$$
  Lemma 4.2 then gives
  $$\begin{aligned}\Vert v^T(s)\Vert _{H^1}\le 2\eta +\Vert v(T+s)\Vert _{H^1} <\delta _*, \end{aligned}$$
  which contradicts (8.2).
3. The event ${\mathcal {A}}_3$ while also ${\mathcal {S}}_{n-1}(\eta )$ implies
  $$\begin{aligned}\tau ^{n\Delta T}_{\textrm{st}}(\eta )\le \Delta T \ \ \text {while} \quad \Vert v(n\Delta T)\Vert _{H^1_w}\le \tfrac{\eta }{3M}\end{aligned}$$
  and
  $$\begin{aligned}\tau ^{n\Delta T}_{\textrm{st}}(\eta ) \le \min \big \{\tau ^{n\Delta T}_{\textrm{en}}(\delta _*),\tau ^{n\Delta T}_{\textrm{mod}}(\eta ),\tau ^{n\Delta T}_c\big \}.\end{aligned}$$
  Via Proposition 5.1, we then obtain
  $$\begin{aligned}\mathbb {P}\Big [{\mathcal {S}}_{n-1}(\eta )\cap {\mathcal {G}}_n(\eta )\cap {\mathcal {A}}_3\Big ]\le e^{-\delta _9\eta ^2/\sigma ^2}.\end{aligned}$$

Summarizing our results so far, we have shown that

$$\begin{aligned} \mathbb {P}\Big [{\mathcal {S}}_{n-1}(\eta )\cap {\mathcal {G}}_n(\eta ) \cap {\mathcal {A}}\Big ]\le e^{-\delta _{12}\eta ^2/\sigma ^2}+e^{-\delta _9\eta ^2/\sigma ^2}. \end{aligned}$$

To complete the proof, we turn our attention to item (ii) of ${\mathcal {S}}_n$, which fails to hold in the event that

$$\begin{aligned}{\mathcal {B}}=\{ \Vert v^{n\Delta T}(\Delta T)\Vert _{H^1_w}> \tfrac{\eta }{9M}\} \cup \{\Vert v((n+1)\Delta T)\Vert _{H^1_w}> \tfrac{\eta }{3M}\}.\end{aligned}$$

Note that the event

$$\begin{aligned}\Vert v^{n\Delta T}(\Delta T)\Vert _{H^1_w}> \tfrac{\eta }{9M}\quad \text {while} \quad {\mathcal {S}}_{n-1}(\eta )\quad \text {and} \quad {\mathcal {A}}^c \quad \text {hold}\end{aligned}$$

occurs with probability less than $e^{-\delta _9\eta ^2/\sigma ^2}$ through Proposition 5.1. In the likely case that

$$\begin{aligned}\Vert v^{n\Delta T}(\Delta T)\Vert _{H^1_w}\le \tfrac{\eta }{9M},\end{aligned}$$

it follows via Lemma 4.2 that also

$$\begin{aligned}\Vert v((n+1)\Delta T)\Vert _{H^1_w}\le \tfrac{\eta }{3M}.\end{aligned}$$

Hence,

$$\begin{aligned}\mathbb {P}\Big [{\mathcal {S}}_{n-1}(\eta ) \cap {\mathcal {G}}_n(\eta )\cap {\mathcal {B}}\cap {\mathcal {A}}^c\Big ]\le e^{-\delta _9\eta ^2/\sigma ^2}.\end{aligned}$$

The proof is then completed by noting that

$$\begin{aligned} {\mathcal {S}}_{n-1}(\eta )\cap {\mathcal {G}}_n(\eta ) \cap {\mathcal {S}}_n^c=\Big ({\mathcal {S}}_{n-1}(\eta )\cap {\mathcal {G}}_n(\eta ) \cap {\mathcal {A}}\Big )\cup \Big ({\mathcal {S}}_{n-1}(\eta )\cap {\mathcal {G}}_n(\eta ) \cap {\mathcal {B}}\Big ), \end{aligned}$$

where the probability of the latter can be estimated as

$$\begin{aligned}\mathbb {P}\Big [{\mathcal {S}}_{n-1}(\eta )\cap {\mathcal {G}}_n(\eta ) \cap {\mathcal {B}}\Big ]\le \mathbb {P}\Big [{\mathcal {S}}_{n-1}(\eta )\cap {\mathcal {G}}_n(\eta )\cap {\mathcal {A}}\Big ]+\mathbb {P}\Big [{\mathcal {S}}_{n-1}(\eta )\cap {\mathcal {G}}_n(\eta ) \cap {\mathcal {B}}\cap {\mathcal {A}}^c\Big ]. \end{aligned}$$

□

We are now in shape to prove Theorem 1.1. Given $c_*,E>0$, we fix $c_{\textrm{min}}$ and $c_{\textrm{max}}$ via C2. Picking $w\in (0,\sqrt{c_{\textrm{min}}/3})$ then ensures that we are in the setting of S2. Lastly, let the constants $\Delta T,\delta _*$ and $\eta _0$ satisfy C1. We set out to control the probability of the slightly larger event

$$\begin{aligned}{\mathcal {C}}(\eta )=\{\min \{t_{\textrm{st}}(2\eta ),t_c,t_{\textrm{en}}(\delta _*/2)\}<T\}.\end{aligned}$$

Writing ${\overline{t}}=\min \{t_{\textrm{st}}(2\eta ),t_c,t_{\textrm{en}}(\delta _*/2)\}$, we categorize ${\mathcal {C}}(\eta )$ into three scenarios:

$$\begin{aligned} {\mathcal {C}}_1(\eta )=&\{t_c<T\}\cap \{t_c\le {\overline{t}}\};\\ {\mathcal {C}}_2(\eta )=&\{t_{\textrm{en}}(\delta _*/2)<T\}\cap \{t_{\textrm{en}}(\delta _*/2)\le {\overline{t}}\};\\ {\mathcal {C}}_3(\eta )=&\{t_{\textrm{st}}(2\eta )<T\}\cap \{t_{\textrm{st}}(2\eta )\le {\overline{t}}\}, \end{aligned}$$

corresponding to which of the stopping times is hit first. We now subdivide the event ${\mathcal {C}}_3(\eta )$ by noting that for each realisation $\omega \in {\mathcal {C}}_3(\eta )$, the stopping time $t_{\textrm{st}}(2\eta )(\omega )$ is contained in an interval

$$\begin{aligned}\big [n(\omega )\Delta T, n(\omega )\Delta T+\Delta T\big ),\quad n(\omega )\in \{0, 1,\ldots ,\lceil T/\Delta T \rceil -1\}.\end{aligned}$$

In view of Lemma 6.5, we in turn find that

$$\begin{aligned}n(\omega )\Delta T+\min \{\tau ^{n(\omega )\Delta T}_{\textrm{st}}(\eta )(\omega ),\tau ^{n(\omega )\Delta T}_{\textrm{mod}}(\eta )(\omega )\}\le t_\textrm{st}(2\eta )(\omega ).\end{aligned}$$

Hence, ${\mathcal {C}}_3(\eta )$ implies that either

$$\begin{aligned} {\mathcal {C}}_{3;i}(\eta ):=\bigcup _{n=1}^{\lceil T/\Delta T \rceil -1} \Big ( {\mathcal {S}}_{n-1}(\eta )\cap {\mathcal {G}}_{n}(\eta ) \cap {\mathcal {S}}^c_n(\eta )\Big );\end{aligned}$$

i.e. the ‘chain’ of stable events was interrupted, or stability failed on the first interval

$$\begin{aligned} {\mathcal {C}}_{3;ii}(\eta ):={\mathcal {S}}_{0}^c(\eta )\cap {\mathcal {G}}_{0}(\eta ).\end{aligned}$$

In summary, we have obtained

8.3 $$\begin{aligned} {\mathcal {C}}(\eta )\subseteq {\mathcal {C}}_1(\eta )\cup {\mathcal {C}}_2(\eta )\cup {\mathcal {C}}_{3;i}(\eta )\cup {\mathcal {C}}_{3;ii}(\eta ). \end{aligned}$$

Each of these events can be readily controlled using our prior results.

### Proof of Theorem 1.1

For ease of exposition, we consider T≥1 for which $T/(\Delta T)\in {\mathbb {N}}$. We proceed by bounding the probability of each of the events ${\mathcal {C}}_1(\eta ), {\mathcal {C}}_2(\eta ), {\mathcal {C}}_{3;i}(\eta )$ and ${\mathcal {C}}_{3;ii}(\eta )$ in (8.3).

1. Proposition 7.1 gives
  8.4 $$\begin{aligned} \mathbb {P}({\mathcal {C}}_1(\eta ))\le e^{-\delta _{17}/(\sigma ^2 T)}.\end{aligned}$$
2. Proposition 7.2 gives
  8.5 $$\begin{aligned} \mathbb {P}\Big [{\mathcal {C}}_2(\eta )\Big ]\le C_{16}(\delta _*/2)^{-1}\Big (T\big ( \sigma ^2+2\epsilon \eta +4\eta ^2\big )+2\sqrt{T}\sigma \eta \Big ).\end{aligned}$$
3. Applying Proposition 8.1 on the T/ΔT-1 intervals yields
  8.6 $$\begin{aligned} \mathbb {P}({\mathcal {C}}_{3;i}(\eta ))\le \Big (\frac{T}{\Delta T}-1\Big ) C_{19}e^{-\delta _{19}\eta ^2/\sigma ^2}.\end{aligned}$$
  Similarly,
  8.7 $$\begin{aligned} \mathbb {P}[{\mathcal {C}}_{3;ii}(\eta )]\le C_{19}e^{-\delta _{19}\eta ^2/\sigma ^2}. \end{aligned}$$

The bounds (8.4), (8.5), (8.6) and (8.7) together with the union bound

$$\begin{aligned}\mathbb {P}[{\mathcal {C}}(\eta )]\le \mathbb {P}[{\mathcal {C}}_1(\eta )]+\mathbb {P}[{\mathcal {C}}_2(\eta )]+\mathbb {P}[{\mathcal {C}}_{3;i}(\eta )]+\mathbb {P}[{\mathcal {C}}_{3;ii}(\eta )]\end{aligned}$$

imply that

8.8 $$\begin{aligned} \mathbb {P}(t_{\textrm{st}}(2\eta )<T)\le N(\eta ,\sigma ,T),\end{aligned}$$

where

$$\begin{aligned}N(\eta ,\sigma ,T)={\tilde{C}}T(\eta ^2+e^{-\delta _{19} \eta ^2/\sigma ^2}),\end{aligned}$$

for a sufficiently large constant ${\tilde{C}}>0$. Observe now that for any ${\tilde{\eta }}\in [0,\eta ]$, it $\mathbb {P}$-almost surely holds that $t_{\textrm{st}}(2{\tilde{\eta }})\le t_{\textrm{st}}(2\eta )$. We hence have the inclusion

$$\begin{aligned}\{t_{\textrm{st}}(2\eta )<T\}\subseteq \{t_{\textrm{st}}(2{\tilde{\eta }})<T\},\end{aligned}$$

allowing (8.8) to be improved to

$$\begin{aligned}\mathbb {P}[t_{\textrm{st}}(2\eta )<T]\le \inf _{0\le {\tilde{\eta }}\le \eta } N(\tilde{\eta },\sigma ,T).\end{aligned}$$

For $\sigma ^2< \delta _{19}$, we compute that $N(\tilde{\eta },\sigma ,T)$ is minimized at ${\tilde{\eta }}^2=\frac{\sigma ^2}{\delta _{19}}\log (\frac{\delta _{19}}{\sigma ^2})$, and

$$\begin{aligned}\inf _{{\tilde{\eta }}\ge 0 }N(\tilde{\eta },\sigma ,T)=\frac{{\tilde{C}}}{\delta _{19}}T{\sigma ^2}\Big (\log \Big (\frac{\delta _{19}}{\sigma ^2}\Big )+1\Big ).\end{aligned}$$

Hence, in case $\eta ^2\le \frac{\sigma ^2}{\delta _{19}}\log (\frac{\delta _{19}}{\sigma ^2})$, we have

$$\begin{aligned}\mathbb {P}[t_{\textrm{st}}(2\eta )<T]\le N(\eta ,\sigma ,T)\le {\tilde{C}}T\Big (\frac{\sigma ^2}{\delta _{19}}\log \Big (\frac{\delta _{19}}{\sigma ^2}\Big )+e^{-\delta _{19} \eta ^2/\sigma ^2}\Big ).\end{aligned}$$

On the other hand, for $\eta ^2> \frac{\sigma ^2}{\delta _{19}}\log (\frac{\delta _{19}}{\sigma ^2})$, we find

$$\begin{aligned}\mathbb {P}[t_{\textrm{st}}(2\eta )<T]\le \inf _{{\tilde{\eta }}\ge 0 }N(\tilde{\eta },\sigma ,T)=\frac{{\tilde{C}}}{\delta _{19}}T{\sigma ^2}\Big (\log \Big (\frac{\delta _{19}}{\sigma ^2}\Big )+1\Big ).\end{aligned}$$

Both estimates can be absorbed by the bound (1.9), completing the proof. □

## Validity of reduced dynamics

Our goal here is to establish the remaining approximation result Theorem 1.2. This result concerns the validity of the approximation $c_{\textrm{ap}}(t)$ defined in (1.7) for the soliton amplitude *c*(*t*). We thus set out to analyze the evolution equation

9.1 $$\begin{aligned} {\mathrm d}\big (c(t)-c_{\textrm{ap}}(t)\big )=&c^0_d(v,c)\ {\mathrm d}t+\epsilon f(t)\big (c_f(v,c)-\tfrac{4}{3}c_{\textrm{ap}}\big )\ {\mathrm d}t+\sigma ^2 \big (c_d(v,c)-c_d(0,c_{\textrm{ap}})\big ) \ {\mathrm d}t\nonumber \\&+\sigma \big \langle c_s(v,c)-\tfrac{2}{9}c_{\textrm{ap}}^{-1/2}\phi _{c_{\textrm{ap}}}^2, T_{\xi } {\mathrm d}W_t^Q\big \rangle , \end{aligned}$$

which one finds by subtracting (1.7) from (3.3). Recalling the constants $C_2$ and $\delta _2$ introduced in Lemma 3.3, we obtain the following useful bounds on the terms above.

### Lemma 9.1

Assuming S1 and S2, there exists a constant $C_{20}>0$ so that for all $c_1,c_{2}\in [\tfrac{1}{2}c_{\textrm{min}},2c_{\textrm{max}}]$ and all $v\in L^2_w$ that satisfy $\Vert v\Vert _{L^2_w}\le \delta _2$, we have

$$\begin{aligned} \big |c_f(v,c_1)-\tfrac{4}{3}c_{2}\big |\le&C_2\Vert v\Vert _{L_w^2}+\tfrac{4}{3}|c_1-c_{2}|,\\ \big |c_d(v,c_1)-c_d(0,c_{2})\big |\le&C_2\big (1+\Vert v\Vert _{L^2_w}+\Vert v\Vert _{L^2_w}^2\big )\Vert v\Vert _{L^2_w}+C_{20}|c_1-c_{2}|, \\ \big \Vert Q^{1/2}c_s(v,c_1)-Q^{1/2}[\tfrac{2}{9}c_{2}^{-1/2}\phi _{c_{2}}^2]\big \Vert _{L^2}\le&C_2\Vert v\Vert _{L_w^2}+C_{20}|c_1-c_{2}|.\end{aligned}$$

### Proof

Recalling that $c_f(0,c)=\tfrac{4}{3}c$, we estimate

$$\begin{aligned}\big |c_f(v,c_1)-\tfrac{4}{3}c_{2}\big |\le \big |c_f(v,c_1)-c_f(0,c_1)\big |+\big |\tfrac{4}{3}c_1-\tfrac{4}{3}c_{2}\big |.\end{aligned}$$

Applying Lemma 3.3 to estimate the first term yields

$$\begin{aligned}\big |c_f(v,c_1)-\tfrac{4}{3}c_{2}\big |\le C_2\Vert v\Vert _{L_w^2}+\tfrac{4}{3}|c_1-c_{2}|.\end{aligned}$$

The remaining inequalities follow analogously through the Lipschitz bound

$$\begin{aligned} |c_d(0,c_1)-c_d(0,c_2)|+\Vert Q^{1/2}c_s(0,c_1)-Q^{1/2}c_s(0,c_2)\Vert _{L^2}\le C_{20}|c_1-c_2| \end{aligned}$$

on $$[\tfrac{1}{2}c_{\textrm{min}},2c_{\textrm{max}}]$$. □

As a further preparation, we establish control on the stochastic integral in (9.1). Recall the notation

$$\begin{aligned}t_{\textrm{ap}}(\lambda )=\sup \big \{t\ge 0:|c(t)-c_{\textrm{ap}}(t)|\le \lambda \big \},\end{aligned}$$

and let $$\lambda _0=\min \{\tfrac{1}{2}c_{\textrm{min}},c_{\textrm{max}}\}$$.

### Lemma 9.2

Assuming S1, S2 and C1, there exists a constant $C_{21}>0$ so that for each T≥0 and $\eta \in [0, \delta _2]$ we have

$$\begin{aligned}&\mathbb {E}\sup _{0\le t \le T\wedge t_{c}\wedge t_{\textrm{ap}}(\lambda _0)\wedge t_{\textrm{st}}(\eta )} \Big | \int _0^t \langle c_s(v,c)-\tfrac{2}{9}c_{\textrm{ap}}^{-1/2}\phi _{c_{\textrm{ap}}}^2, T_{\xi } {\mathrm d}W_{t'}^Q \rangle \Big | \\&\quad \le C_{21} \sqrt{T}\mathbb {E}\sup _{0\le {t'} \le T \wedge t_{\textrm{st}}(\eta )}\Vert v({t'})\Vert _{L^2_w} +C_{21} \sqrt{T}\mathbb {E}\sup _{0\le {t'} \le T\wedge t_{c}\wedge t_{\textrm{ap}}(\lambda _0)} |c({t'})-c_{\textrm{ap}}({t'})|. \end{aligned}$$

### Proof

Let us write ${\overline{t}}= t_{c}\wedge t_{\textrm{ap}}(\lambda _0)\wedge t_{\textrm{st}}(\eta )$. The Burkholder-Davis-Gundy inequality [26, proposition 2.1] provides control of the stochastic integral:

$$\begin{aligned}&\mathbb {E}\sup _{0\le t \le T\wedge {\overline{t}}} \Big |\int _0^t \langle c_s(v,c)-\tfrac{2}{9}c_{\textrm{ap}}^{-1/2}\phi _{c_{\textrm{ap}}}^2, T_{\xi } {\mathrm d}W_{t'}^Q \rangle \Big |\\&\le {\tilde{C}}_1 \mathbb {E}\Big [ \Big (\int _0^{T\wedge {\overline{t}}}\big \Vert Q^{1/2}c_s(v,c)-Q^{1/2}[\tfrac{2}{9}c_{\textrm{ap}}^{-1/2}\phi _{c_{\textrm{ap}}}^2]\big \Vert _{L^2}^2{\mathrm d}t'\Big )^{1/2} \Big ].\end{aligned}$$

Lemma 9.1 then yields

$$\begin{aligned}&\mathbb {E}\sup _{0\le t \le T\wedge {\overline{t}}} \Big |\int _0^t \langle c_s(v,c)-\tfrac{2}{9}c_{\textrm{ap}}^{-1/2}\phi _{c_{\textrm{ap}}}^2, T_{\xi } {\mathrm d}W_{t'}^Q \rangle \Big |\\ \le&{\tilde{C}}_2 \mathbb {E}\Big [ \Big (\int _0^{T\wedge {\overline{t}}}\Vert v\Vert ^2_{L^2_w}+|c-c_{\textrm{ap}}|^2{\mathrm d}t\Big )^{1/2} \Big ]\\ \le&{\tilde{C}}_2 \sqrt{T}\mathbb {E}\sup _{0\le t \le T \wedge t_{\textrm{st}}(\eta )}\Vert v(t)\Vert _{L^2_w}+{\tilde{C}}_2 \sqrt{T}\mathbb {E}\sup _{0\le t \le T\wedge t_{c}\wedge t_{\textrm{ap}}(\lambda _0)} |c(t)-c_{\textrm{ap}}(t)|. \end{aligned}$$

□

We are now ready to control $|c(t)-c_{\textrm{ap}}(t)|$ via a Grönwall argument, resulting in conditional control of the stopping time $t_{\textrm{ap}}$.

### Lemma 9.3

Assuming S1, S2, C1 and C2, there exists a constant $C_{22}>0$ so that for each $T\ge 1, \eta \in (0,\delta _2]$, each $\sigma ,\epsilon \in [0,\eta ]$ and each $\lambda \in (0,\lambda _0]$, we have

$$\begin{aligned}\mathbb {P}\big [t_{\textrm{ap}}(\lambda )<T\cap t_{\textrm{st}}(\eta )\wedge t_c \ge T\big ]\le C_{22}Te^{\sigma ^2 T}\frac{\eta ^2}{\lambda }.\end{aligned}$$

### Proof

Applying Lemma 9.1 and Lemma 9.2 to the SDE (9.1) yields

$$\begin{aligned} |c(t)-c_{\textrm{ap}}(t)|\le&C_2\int _0^t \Vert v\Vert ^2_{L^2_w}{\mathrm d}t'+C_2\epsilon \int _0^t \Vert v\Vert _{L^2_w}{\mathrm d}t'+\tfrac{4}{3}\epsilon \int _0^t|f(t')||c-c_{\textrm{ap}}|{\mathrm d}t'\\&+C_2\sigma ^2 \int _0^t (1+\Vert v\Vert _{L^2_w}^2)\Vert v\Vert _{L^2_w}{\mathrm d}t'+C_{20}\sigma ^2\int _0^t|c-c_{\textrm{ap}}| \ {\mathrm d}t'\\&+\sigma \Big |\int _0^t \langle c_s(v,c)-\tfrac{2}{9}c_{\textrm{ap}}^{-1/2}\phi _{c_{\textrm{ap}}}^2, T_{\xi } {\mathrm d}W_{t'}^Q\rangle \Big |, \end{aligned}$$

for all $t\in [0,t_{c}\wedge t_{\textrm{ap}}(\lambda _0)]$. Let us once more write ${\overline{t}}=t_{c}\wedge t_{\textrm{ap}}(\lambda _0)\wedge t_{\textrm{st}}(\eta )$. In addition, we introduce the notation

$$\begin{aligned}E(t):=\mathbb {E}\sup _{0\le t' \le t\wedge {\overline{t}}} |c(t')-c_{\textrm{ap}}(t')|,\quad t\ge 0.\end{aligned}$$

Inspecting the bound above implies

9.2 $$\begin{aligned} E(T)\le&C_2T \eta ^2+C_2\epsilon T \eta +\tfrac{4}{3}\epsilon \int _0^T|f(t)| E(t){\mathrm d}t\nonumber \\&+C_2\sigma ^2 T (1+\eta ^2)\eta +C_{20}\sigma ^2\int _0^TE(t){\mathrm d}t \nonumber \\&+\sigma C_{21} \sqrt{T} \Big (\eta +E(T)\Big ). \end{aligned}$$

Imposing the restriction $\sigma \sqrt{T}\le \frac{1}{2C_{21}}$, we may bring the last term in (9.2) to the left to find

$$\begin{aligned} \tfrac{1}{2}E(T)\le&C_2T \eta ^2+C_2\epsilon T \eta +\tfrac{4}{3}\epsilon \int _0^T|f(t)| E(t){\mathrm d}t\\&+C_2\sigma ^2 T (1+\eta ^2)\eta +C_{20}\sigma ^2\int _0^TE(t) {\mathrm d}t+C_{21}\sigma \eta \sqrt{T}. \end{aligned}$$

Grönwall’s inequality then yields

$$\begin{aligned} \tfrac{1}{2}E(T)\le&C_2\exp \Big ( \int _0^T C_{20}\sigma ^2+\frac{4}{3}\epsilon |f(t)|{\mathrm d}t\Big )\\&\times \Big (T \eta ^2+\epsilon T \eta +\sigma ^2 T (1+\eta ^2)\eta +\frac{C_{21}}{C_2}\sigma \eta \sqrt{T} \Big ), \end{aligned}$$

in which C2 ensures

$$\begin{aligned}\exp \Big ( \int _0^T C_{20}\sigma ^2+\frac{4}{3}\epsilon |f(t)|{\mathrm d}t\Big )\le \exp \Big ( C_{20}\sigma ^2T+\frac{4}{3}E\Big ).\end{aligned}$$

Markov’s inequality finally yields

$$\begin{aligned} \mathbb {P}\Big [\sup _{0\le t' \le T\wedge {\overline{t}}} |c(t')-c_{\textrm{ap}}(t')|\ge \lambda \Big ]\le&\frac{2}{\lambda } C_2\exp \Big ( C_{20}\sigma ^2T+\frac{4}{3}E\Big )\\&\times \Big (T \eta ^2+\epsilon T \eta +\sigma ^2 T (1+\eta ^2)\eta +\frac{C_{21}}{C_2}\sigma \eta \sqrt{T} \Big ). \end{aligned}$$

To complete the proof, note that $t_{\textrm{ap}}(\lambda )<T$ while $t_{\textrm{st}}(\eta )\wedge t_c\ge T$ implies

$$\begin{aligned}\sup _{0\le t'\le T\wedge {\overline{t}}}|c(t')-c_{\textrm{ap}}(t')|> \lambda .\end{aligned}$$

□

### Proof of Theorem 1.2

For any $\eta \ge 0$, we have the union bound

$$\begin{aligned}\mathbb {P}[t_{\textrm{ap}}(\lambda )<T]\le \mathbb {P}[t_{\textrm{ap}}(\lambda )<T\cap t_{\textrm{st}}(\eta )\wedge t_c\ge T]+\mathbb {P}[t_{\textrm{st}}(\eta )\wedge t_c<T]\end{aligned}$$

and hence

$$\begin{aligned}\mathbb {P}[t_{\textrm{ap}}(\lambda )<T]\le \inf _{\eta \ge 0}\Big (\mathbb {P}[t_{\textrm{ap}}(\lambda )<T\cap t_{\textrm{st}}(\eta )\wedge t_c\ge T]+\mathbb {P}[t_{\textrm{st}}(\eta )\wedge t_c<T]\Big ).\end{aligned}$$

Applying Theorem 1.1 and Lemma 9.3 now gives

$$\begin{aligned}\mathbb {P}[t_{\textrm{ap}}(\lambda )<T]\le (C+C_{22})T \inf _{\eta \ge 0}\Big (\frac{\eta ^2}{\lambda }+e^{-\delta \eta ^2/\sigma ^2}\Big )+CT\sigma ^2\log (1/\sigma ).\end{aligned}$$

In case $\sigma ^2<\lambda \delta$, the infimum is attained at

$$\begin{aligned}\eta ^2=\frac{\sigma ^2}{\delta }\log \Big (\frac{\lambda \delta }{\sigma ^2}\Big )\end{aligned}$$

yielding

$$\begin{aligned}\mathbb {P}[t_{\textrm{ap}}(\lambda )<T]\le (C+C_{22})T {\frac{\sigma ^2}{\lambda \delta }\Big (\log \Big (\frac{\lambda \delta }{\sigma ^2}\Big )}+1\Big )+CT\sigma ^2\log (1/\sigma ).\end{aligned}$$

Upon increasing the constant *C* if necessary, we obtain the result (1.10). □

## Data Availability

No datasets were generated or analysed during the current study.

## Stopping times

Here, we provide an overview of the various stopping times introduced throughout the work. In relation to the global modulation system $\big (v(t),c(t),\xi (t)\big )$ introduced in Section 3, we use the stopping times

$$\begin{aligned} t_c=&\sup \{t\ge 0:c(t) \in [c_{\textrm{min}},c_{\textrm{max}}]\};\\ t_{\textrm{log}}=&\sup \{t\ge 0 :|\log (c(t)/c_*)-\frac{4}{3}\epsilon \int _0^t|f(t')|{\mathrm d}t'|\le E\};\\ t_{\textrm{st}}(\eta )=&\sup \big \{t\ge 0:\Vert v(t)\Vert _{H_w^1}\le \eta \big \};\\ t_{\textrm{en}}(\eta )=&\sup \big \{t\ge 0:\big \Vert v(t)\big \Vert _{L^2}\le \eta \big \};\\ t_{\textrm{ap}}(\eta )=&\sup \big \{t\ge 0:|c(t)-c_{\textrm{ap}}(t)|\le \eta \big \}. \end{aligned}$$

For each T≥0, the following stopping times are related to the local modulation system $\big (v^T(s),c^T(s),\xi ^T(s)\big )$ introduced in Section 4:

$$\begin{aligned}\tau ^T_{\textrm{st}}(\eta )=&\sup \big \{s\ge 0:\big \Vert v^T(s)\big \Vert _{H^1_w}\le \eta \big \};\\ \tau ^T_c=&\sup \big \{s\ge 0:c(T+s)\in [\tfrac{1}{2}c_{\textrm{min}},2c_{\textrm{max}}] \big \};\\ \tau ^T_{\textrm{en}}(\eta )=&\sup \big \{s\ge 0:\big \Vert v^T(s)\big \Vert _{L^2}\le \eta \big \};\\ \tau ^{T}_{\textrm{amp,1}}(\eta ) =&\sup \big \{s\ge 0:\big |c(T)-c^T(s)\big |\le \delta _*\eta \}; \\ \tau ^{T}_{\textrm{amp,2}}(\eta ) =&\sup \big \{s\ge 0:\big |c(T+s)-c^T(s)\big |\le \delta _*\eta \}; \\ \tau ^{T}_{\textrm{pos,1}}(\eta ) =&\sup \big \{s\ge 0:\big |\xi (T)+c(T)s-\xi ^T(s)\big |\le 2\Delta T \delta _*\eta \big \};\\ \tau ^{T}_{\textrm{pos,2}}(\eta ) =&\sup \big \{s\ge 0:\big |\xi (T+s)-\xi ^T(s)\big |\le 2\Delta T \delta _*\eta \big \}, \end{aligned}$$

and

$$\begin{aligned} \tau ^T_{\textrm{mod}}(\eta )=\tau ^{T}_{\textrm{amp},1}(\eta )\wedge \tau ^{T}_{\textrm{amp},2}(\eta )\wedge \tau ^{T}_{\textrm{pos,1}}( \eta )\wedge \tau ^{T}_{\textrm{pos,2}}( \eta ). \end{aligned}$$

## Technical proofs

Here, we provide the proofs of various lemmas which were omitted in Section 3, Section 4 and Section 5.

### Proof of Lemma 3.2

We derive the evolution equation for $\big \langle v(t),\phi _c(t)\big \rangle$, noting that the remaining evolution equation for $\big \langle v(t),\zeta _c(t)\big \rangle$ follows analogously. First, we introduce the notation

$$\begin{aligned}\big \langle v(t),\phi _{c(t)}\big \rangle  &   =\big \langle u(t,\cdot +\xi (t)),\phi _{c(t)}\big \rangle -\big \langle \phi _{c(t)},\phi _{c(t)}\big \rangle \\  &   =\big \langle u(t),\phi _{c(t)}(\cdot -\xi (t))\big \rangle -6c^{3/2}(t)=:F\big (u(t),c(t),\xi (t)\big ).\end{aligned}$$

We now apply the mild Itô formula to the functional $F:H^1\times {\mathbb {R}}\times {\mathbb {R}}\rightarrow {\mathbb {R}}$. We therefore interpret the tuple (u,c,ξ) as a mild process with respect to the $C_0$-group $\{S(t)\}_{t\in {\mathbb {R}}}$ given by

$$\begin{aligned} S(t)=\begin{bmatrix} e^{-\partial _x^3t} &  0 &  0\\ 0 &  I_{{\mathbb {R}}} & 0\\ 0 &  0 &  I_{{\mathbb {R}}} \end{bmatrix}, \quad t\in {\mathbb {R}}. \end{aligned}$$

We collect that *F* is twice Fréchet differentiable, with first derivatives

$$\begin{aligned} {\mathrm d}_uF(u,c,\xi )[v]=&\big \langle v,\phi _c(\cdot -\xi )\big \rangle ,\\ {\mathrm d}_cF(u,c,\xi )=&\big \langle u,\partial _c\phi _c(\cdot -\xi )\big \rangle -9c^{1/2},\\ {\mathrm d}_{\xi } F(u,c,\xi )=&-\big \langle u,\partial _x\phi _c(\cdot -\xi )\big \rangle , \end{aligned}$$

and second derivatives

$$\begin{aligned} {\mathrm d}^2_{uu}F(u,c,\xi )[v,w]=&0,\\ {\mathrm d}^2_{cc}F(u,c,\xi )=&\big \langle u,\partial ^2_c\phi _c(\cdot -\xi )\big \rangle -\tfrac{9}{2}c^{-1/2},\\ {\mathrm d}^2_{\xi \xi }F(u,c,\xi )=&\big \langle u,\partial _x^2\phi _c(\cdot -\xi )\big \rangle ,\\ {\mathrm d}^2_{uc}F(u,c,\xi )[v]=&\big \langle v,\partial _c\phi _c(\cdot -\xi )\big \rangle ,\\ {\mathrm d}^2_{u\xi }F(u,c,\xi )[v]=&-\big \langle v,\partial _x\phi _c(\cdot -\xi )\big \rangle ,\\ {\mathrm d}^2_{c\xi }F(u,c,\xi )=&-\big \langle u,\partial ^2_{cx}\phi _c(\cdot -\xi )\big \rangle . \end{aligned}$$

For any orthonormal basis $\{e_k\}_{k=0}^\infty$ of $L^2$, [6, Theorem 1] then yields:

B.1 $$\begin{aligned} F\big (u(t),c(t),\xi (t)\big )=&F\big (e^{-\partial _x^3t}\phi _{c_*},c_*,0\big )+\int _0^tF_1(t,t'){\mathrm d}t'+\sigma ^2\int _0^tF_2(t,t'){\mathrm d}t'\nonumber \\&+\sigma ^2\sum _{k=0}^\infty \int _0^tF_3(t,t',k){\mathrm d}t' +\sigma \int _0^tF_4(t,t'){\mathrm d}W^ Q_{t'}, \end{aligned}$$

with

$$\begin{aligned} F_1(t,t')=&{\mathrm d}_uF\big (e^{-\partial _x^3(t-t')}u,c,\xi \big )\big [ e^{-\partial _x^3 (t-t')}(-\partial _x(u^2)+\epsilon f(t')u)\big ]\\&+ {\mathrm d}_cF\big (e^{-\partial _x^3(t-t')}u,c,\xi \big ) c_d^{\sigma ,\epsilon } + {\mathrm d}_{\xi } F\big (e^{-\partial _x^3(t-t')}u,c,\xi \big ) \xi _d^{\sigma ,\epsilon },\\ F_2(t,t')=&\tfrac{1}{2}{\mathrm d}^2_{cc}F\big (e^{-\partial _x^3(t-t')}u,c,\xi )\big \Vert Q^{1/2}c_s \Vert _{L^2}^2+\tfrac{1}{2}{\mathrm d}^2_{\xi \xi }F\big (e^{-\partial _x^3(t-t')}u,c,\xi \big )\Vert Q^{1/2}\xi _s \Vert _{L^2}^2\\&+{\mathrm d}^2_{c\xi }F\big (e^{-\partial _x^3(t-t')}u,c,\xi \big )\langle Q^{1/2}\xi _s ,Q^{1/2}c_s \rangle ,\\ F_3(t,t',k)=&{\mathrm d}^2_{uc}F\big (e^{-\partial _x^3(t-t')}u,c,\xi \big ) [e^{-\partial _x^3(t-t')}uQ^{1/2}e_k]\langle c_s ,T_{\xi } Q^{1/2}e_k\rangle \\&+ {\mathrm d}^2_{u\xi }F\big (e^{-\partial _x^3(t-t')}u,c,\xi \big ) [e^{-\partial _x^3(t-t')}uQ^{1/2}e_k]\langle \xi _s ,T_{\xi } Q^{1/2}e_k\rangle ,\\ F_4(t,t')[h]=&{\mathrm d}_uF\big (e^{-\partial _x^3(t-t')}u,c,\xi \big )[ e^{-\partial _x^3 (t-t')}uh]\\&+ {\mathrm d}_cF\big (e^{-\partial _x^3(t-t')}u,c,\xi \big ) \langle c_s ,T_{\xi } h\rangle +{\mathrm d}_{\xi } F\big (e^{-\partial _x^3(t-t')}u,c,\xi \big ) \langle \xi _s ,T_{\xi }h\rangle . \end{aligned}$$

Above, we have suppressed the dependence of $$c_d^{\sigma ,\epsilon },\xi _d^{\sigma ,\epsilon }$$ on $\left(v\left(t^{\prime }\right),c\left(t^{\prime }\right),t^{\prime }\right)$ and of $$c_s,\xi _s$$ on $\left(v\left(t^{\prime }\right),c\left(t^{\prime }\right)\right)$. Substituting the derivatives, this becomes

$$\begin{aligned} F\big (e^{-\partial _x^3t}\phi _{c_*},c_*,0\big )=&\langle e^{-\partial _x^3t}\phi _{c_*},\phi _{c_*}\rangle -6c_*^{3/2}, \\ F_1(t,t')=&\big \langle e^{-\partial _x^3 (t-t')}(-\partial _x(u^2)+\epsilon f(t')u),\phi _c(\cdot -\xi )\big \rangle \\&+\big (\langle e^{-\partial _x^3(t-t')}u,\partial _c\phi _c(\cdot -\xi )\rangle -9c^{1/2}\big ) c_d^{\sigma ,\epsilon }- \big \langle e^{-\partial _x^3(t-t')}u,\partial _x\phi _c(\cdot -\xi )\big \rangle \xi _d^{\sigma ,\epsilon },\\ F_2(t,t')=&\tfrac{1}{2}\big (\langle e^{-\partial _x^3(t-t')}u,\partial ^2_c\phi _c(\cdot -\xi )\rangle -\tfrac{9}{2}c^{-1/2}\big )\Vert Q^{1/2}c_s\Vert _{L^2}^2\\&+\tfrac{1}{2}\big \langle e^{-\partial _x^3(t-t')}u,\partial _x^2\phi _c(\cdot -\xi )\big \rangle \Vert Q^{1/2}\xi _s\Vert _{L^2}^2\\&-\big \langle e^{-\partial _x^3(t-t')}u,\partial ^2_{cx}\phi _c(\cdot -\xi )\big \rangle \big \langle Q^{1/2}\xi _s,Q^{1/2}c_s\big \rangle ,\\ F_3(t,t',k)=&\big \langle e^{-\partial _x^3(t-t')}uQ^{1/2}e_k,\partial _c\phi _c(\cdot -\xi )\big \rangle \big \langle c_s,T_{\xi } Q^{1/2}e_k\big \rangle \\&-\langle e^{-\partial _x^3(t-t')}uQ^{1/2}e_k,\partial _x\phi _c(\cdot -\xi )\rangle \langle \xi _s,T_{\xi } Q^{1/2}e_k\rangle ,\\ F_4(t,t')[h]=&\big \langle e^{-\partial _x^3 (t-t')}u h,\phi _c(\cdot -\xi )\big \rangle + \big (\langle e^{-\partial _x^3(t-t')}u,\partial _c\phi _c(\cdot -\xi )\rangle -9c^{1/2}\big ) \langle c_s,T_{\xi }h\rangle \\&-\big \langle e^{-\partial _x^3(t-t')}u,\partial _x\phi _c(\cdot -\xi )\big \rangle \langle \xi _s,T_{\xi }h\rangle . \end{aligned}$$

We now show how to convert the mild expression (B.1) into a strong form^2^, focusing on the case $\sigma =\epsilon =0$ for ease of exposition. Differentiating $\big \langle v(t),\phi _{c(t)}\big \rangle$ via Leibniz’ rule gives

$$\begin{aligned} \partial _t\big \langle v(t),\phi _{c(t)}\big \rangle =&\partial _tF\big (u(t),c(t),\xi (t)\big )=\partial _tF\big (e^{-\partial _x^3t}\phi _{c_*},c_*,0\big )\\&+F_1(t,t)+\int _0^t \partial _tF_1(t,t'){\mathrm d}t', \end{aligned}$$

and thus

$$\begin{aligned}&\partial _t\big \langle v(t),\phi _{c(t)}\big \rangle =\big \langle e^{-\partial _x^3t}\phi _{c_*},\partial _x^3\phi _{c_*}\big \rangle - \big \langle \partial _x(u^2(t)),\phi _c(\cdot -\xi )\big \rangle \\&\quad -\int _0^t \big \langle \partial _x\big (u^2(t')\big ),e^{\partial _x^3 (t-t')}\partial _x^3\phi _c(\cdot -\xi )\big \rangle {\mathrm d}{t'}\\&\quad +\big (\langle u(t),\partial _c\phi _c(\cdot -\xi )\rangle -9c^{1/2}\big )c_d^0\big (v(t)\big )\\&\quad +\int _0^t \big \langle u(t'),e^{\partial _x^3(t-t')}\partial _x^3\partial _c\phi _c(\cdot -\xi )\big \rangle c_d^0\big (v(t')\big ){\mathrm d}{t'}\\&\quad -\big \langle u(t),\partial _x\phi _c(\cdot -\xi )\big \rangle \xi _d^0\big (v(t)\big )-\int _0^t \big \langle u(t'),e^{\partial _x^3(t-t')}\partial _x^3\partial _x\phi _c(\cdot -\xi )\big \rangle \xi _d^0\big (v(t')\big ){\mathrm d}{t'}, \end{aligned}$$

where we have moved the semigroup to the other side of the inner products via the adjoint relation $(e^{-\partial _x^3t})^*=e^{\partial _x^3t}$. Now we recognize the mild formula

$$\begin{aligned} \big \langle u(t),\partial _x^3\phi _{c}(\cdot -\xi )\big \rangle =&\big \langle e^{-\partial _x^3t}\phi _{c_*},\partial _x^3\phi _{c_*}\big \rangle -\int _0^t \big \langle \partial _x\big (u^2(t')\big ),e^{\partial _x^3 (t-t')}\partial _x^3\phi _c(\cdot -\xi )\big \rangle {\mathrm d}{t'}\\&+\int _0^t \big \langle u(t'),e^{\partial _x^3(t-t')}\partial _x^3\partial _c\phi _c(\cdot -\xi )\big \rangle c_d^0\big (v(t')\big ){\mathrm d}{t'}\\&-\int _0^t \big \langle u(t'),e^{\partial _x^3(t-t')}\partial _x^3\partial _x\phi _c(\cdot -\xi )\big \rangle \xi _d^0\big (v(t')\big ){\mathrm d}{t'}. \end{aligned}$$

We thus we arrive at the strong form

$$\begin{aligned} {\mathrm d}\big \langle v(t),\phi _{c(t)}\big \rangle =&\big \langle u(t),\partial _x^3\phi _{c}(\cdot -\xi )\big \rangle \ {\mathrm d}t- \big \langle \partial _x\big (u^2(t)\big ),\phi _c(\cdot -\xi )\big \rangle \ {\mathrm d}t\\&+\big (\big \langle u(t),\partial _c\phi _c(\cdot -\xi )\big \rangle -9c^{1/2}\big )c_d^0\big (v(t)\big )\ {\mathrm d}t-\big \langle u(t),\partial _x\phi _c(\cdot -\xi )\big \rangle \xi _d^0\big (v(t)\big )\ {\mathrm d}t. \end{aligned}$$

After substituting $$u(t,\cdot +\xi )=\phi _{c}+v(t)$$:

$$\begin{aligned} {\mathrm d}\big \langle v(t),\phi _{c(t)}\big \rangle =&\big \langle v(t),\partial _x^3\phi _{c}\big \rangle \ {\mathrm d}t- \big \langle \partial _x(\phi _{c}+v(t))^2,\phi _c\big \rangle \ {\mathrm d}t\\&+\big (\big \langle \phi _{c}+v(t),\partial _c\phi _c\big \rangle -9c^{1/2}\big )c_d^0\big (v(t)\big )\ {\mathrm d}t-\big \langle v(t),\partial _x\phi _c\big \rangle \xi _d^0\big (v(t)\big )\ {\mathrm d}t \end{aligned}$$

where rewriting

$$\begin{aligned}\big \langle \partial _x(\phi _{c}+v(t))^2,\phi _c\big \rangle =-2\big \langle v(t),\phi _c\partial _x\phi _c\big \rangle -\big \langle N\big (v(t)\big ),\phi _c\big \rangle \end{aligned}$$

leads to

$$\begin{aligned} {\mathrm d}\big \langle v(t),\phi _{c(t)}\big \rangle =&\big \langle v(t),\partial _x^3\phi _{c}+2\phi _c\partial _x\phi _c-c\partial _x\phi _c\big \rangle \ {\mathrm d}t+ \big \langle N\big (v(t)\big ),\phi _c\big \rangle \ {\mathrm d}t\\&+\big (\langle \phi _{c}+v(t),\partial _c\phi _c\rangle -9c^{1/2}\big )c_d^0\big (v(t)\big )\ {\mathrm d}t-\big \langle v(t),\partial _x\phi _c\big \rangle \Omega _d^0\big (v(t)\big )\ {\mathrm d}t. \end{aligned}$$

Using the traveling wave identity $\partial _x^3\phi _{c}+2\phi _c\partial _x\phi _c-c\partial _x\phi _c=0$, we arrive at the result

$$\begin{aligned}&{\mathrm d}\big \langle v(t),\phi _{c(t)}\big \rangle = \big \langle N\big (v(t)\big ),\phi _c\big \rangle \ {\mathrm d}t+\big (\langle \phi _{c}+v(t),\partial _c\phi _c\rangle -9c^{1/2}\big )c_d^0\big (v(t)\big )\ {\mathrm d}t\\&\quad -\big \langle v(t),\partial _x\phi _c\big \rangle \Omega _d^0\big (v(t)\big )\ {\mathrm d}t. \end{aligned}$$

The σ- and ϵ-dependent terms in (B.1) can be treated analogously, which completes the proof. □

### Proof of Proposition 4.3

We compute the mild form of

$$\begin{aligned}v^T(s,x)=u\big (T+s,x+\xi (T)+c(T)s\big )-\Phi ^T({\textbf{m}}^T(s),s,x).\end{aligned}$$

Recalling that

$$\begin{aligned}\Phi ^T({\textbf{m}}^T(s),s,x):=\phi _{c^T(s)}\big (x+\xi (T)+c(T)s-\xi ^T(s)\big ),\end{aligned}$$

a straightforward application of Itô’s lemma yields

$$\begin{aligned} {\mathrm d}\Phi ^T=&\Big [\partial _c\Phi ^Tc_d^{\sigma ,\epsilon ,T}+\partial _x\Phi ^T(c(T)-c^T-\Omega _d^{\sigma ,\epsilon ,T})\Big ]{\mathrm d}s\\&+\frac{\sigma ^2}{2}\Big [\partial ^2_c\Phi ^T\Vert Q^{1/2}c_s^{T}\Vert _{L^2}^2+\partial ^2_x\Phi ^T\Vert Q^{1/2}\xi _s^{T}\Vert _{L^2}^2\Big ] {\mathrm d}s\\&+\sigma ^2\partial ^2_{xc}\Phi ^T\langle Q^{1/2}c_s^{T},Q^{1/2}\xi _s^T\rangle {\mathrm d}s\\&+\sigma \partial _c\Phi ^T\langle c_s^{T},T_{\xi (T)+c(T)s}{\mathrm d}W^Q_{T+s}\rangle +\sigma \partial _x\Phi ^T\langle \xi _s^{T},T_{\xi (T)+c(T)s}{\mathrm d}W^Q_{T+s}\rangle \end{aligned}$$

where we have suppressed the dependence of $\Phi ^T,c_d^{\sigma ,\epsilon ,T}, \xi _d^{\sigma ,\epsilon ,T},c_s^T$ and $\xi _s^T$ on $({\textbf{m}}^T(s),s)$. Using the traveling wave identity

$$\begin{aligned}0=&-\partial _x^3\Phi ^T-\partial _x(\Phi ^T)^2+c^T\partial _x\Phi ^T\\&={\mathcal {L}}_{c(T)}\Phi ^T+\big (c^T-c(T)\big )\partial _x\Phi ^T-\partial _x\big ((\Phi ^T)^2\big )-2\partial _x\big (\phi _{c(T)}\Phi ^T\big ) \end{aligned}$$

we may pass to the mild form

B.2 $$\begin{aligned} \Phi ^T(s)=&e^{{\mathcal {L}}_{c(T)}s}\phi _{c(T)}+\int _0^s e^{{\mathcal {L}}_{c(T)}(s-s')}\Big [-\partial _x((\Phi ^T)^2)-2\partial _x(\phi _{c(T)}\Phi ^T)\Big ]{\mathrm d}s' \nonumber \\&+\int _0^se^{{\mathcal {L}}_{c(T)}(s-s')}\Big [\partial _c\Phi ^Tc_d^{\sigma ,\epsilon ,T}-\partial _x\Phi ^T\Omega _d^{\sigma ,\epsilon ,T}\Big ]{\mathrm d}s'\nonumber \\&+\frac{\sigma ^2}{2}\int _0^se^{{\mathcal {L}}_{c(T)}(s-s')}\Big [\partial ^2_c\Phi ^T\Vert Q^{1/2}c_s^{T}\Vert _{L^2}^2+\partial ^2_x\Phi ^T\Vert Q^{1/2}\xi _s^{T}\Vert _{L^2}^2\Big ] {\mathrm d}s'\nonumber \\&+\sigma ^2\int _0^se^{{\mathcal {L}}_{c(T)}(s-s')}\partial ^2_{xc}\Phi ^T\langle Q^{1/2}c_s^{T},Q^{1/2}\xi _s^T\rangle {\mathrm d}s'\nonumber \\&+\sigma \int _0^se^{{\mathcal {L}}_{c(T)}(s-s')}\partial _c\Phi ^T\langle c_s^{T},T_{\xi (T)+c(T)s'}{\mathrm d}W^Q_{T+s'}\rangle \nonumber \\&+\sigma \int _0^se^{{\mathcal {L}}_{c(T)}(s-s')}\partial _x\Phi ^T\langle \xi _s^{T},T_{\xi (T)+c(T)s'}{\mathrm d}W^Q_{T+s'}\rangle . \end{aligned}$$

Next, we derive a mild formula for u(T+s,x+ξ(T)+c(T)s) based on the identity

$$\begin{aligned}&u(T+s)=e^{-\partial _x^3 s}u(T)-\int _0^s e^{-\partial _x^3 (s-s')}\partial _x\big (u^2(T+s')\big ){\mathrm d}s'\\&\quad +\epsilon \int _0^s f(T+s')e^{-\partial _x^3 (s-s')}u(T+s'){\mathrm d}s'\\&\quad +\sigma \int _0^s e^{-\partial _x^3 (s-s')}u(T+s'){\mathrm d}W_{T+s'}^Q. \end{aligned}$$

The mild Itô formula [6] now yields

$$\begin{aligned}&u\big (T+s,x+\xi (T)+c(T)s\big )=e^{-\partial _x^3s}u\big (T,x+\xi (T)\big )\\&-\int _0^s e^{-\partial _x^3(s-s')}\partial _x\big (u^2(T+s',\cdot +\xi (T)+c(T)s')\big ){\mathrm d}s'\\&+\epsilon \int _0^s f(T+s')e^{-\partial _x^3(s-s')}u\big (T+s',\cdot +\xi (T)+c(T)s'\big ){\mathrm d}s'\\&+c(T)\int _0^s e^{-\partial _x^3(s-s')}u_x\big (T+s',\cdot +\xi (T)+c(T)s'\big ){\mathrm d}s'\\&+\sigma \int _0^s e^{-\partial _x^3(s-s')}u\big (T+s',\cdot +\xi (T)+c(T)s'\big ) T_{\xi (T)+c(T)s'}{\mathrm d}W^Q_{T+s'}. \end{aligned}$$

Using $-\partial _x^3+c(T)\partial _x={\mathcal {L}}_{c(T)}+2\partial _x(\phi _{c(T)}\cdot )$, we rephrase the formula above in terms of the semigroup $\{e^{{\mathcal {L}}_{c(T)}s}\}_{s\ge 0}$ to find:

B.3 $$\begin{aligned}&u\big (T+s,x+\xi (T)+c(T)s\big )=e^{{\mathcal {L}}_{c(T)}s}u\big (T,x+\xi (T)\big )\nonumber \\&+2\int _0^se^{{\mathcal {L}}_{c(T)}(s-s')}\partial _x\big (\phi _{c(T)}u(T+s',\cdot +\xi (T)+c(T)s')\big ){\mathrm d}s'\nonumber \\&-\int _0^s e^{{\mathcal {L}}_{c(T)}(s-s')}\partial _x\big (u^2(T+s',\cdot +\xi (T)+c(T)s')\big ){\mathrm d}s'\nonumber \\&+\epsilon \int _0^s f(T+s')e^{{\mathcal {L}}_{c(T)}(s-s')}u\big (T+s',\cdot +\xi (T)+c(T)s'\big ){\mathrm d}s'\nonumber \\&+\sigma \int _0^s e^{{\mathcal {L}}_{c(T)}(s-s')}u\big (T+s',\cdot +\xi (T)+c(T)s'\big ) T_{\xi (T)+c(T)s'}{\mathrm d}W^Q_{T+s'}. \end{aligned}$$

Subtracting the mild formula (B.2) from (B.3), we arrive at

$$\begin{aligned} v^T(s)=&e^{{\mathcal {L}}_{c(T)}s}v(T)-\int _0^s e^{{\mathcal {L}}_{c(T)}(s-s')}\big [\partial _x((v^T)^2)+2\partial _x\big ((\phi _{c(T)}-\Phi ^T)v^T\big )\big ]{\mathrm d}s'\\&+\epsilon \int _0^s f(T+s')e^{{\mathcal {L}}_{c(T)}(s-s')}\big [\Phi ^T+v^T\big ]{\mathrm d}s'\\&-\int _0^se^{{\mathcal {L}}_{c(T)}(s-s')}\Big [\partial _c\Phi ^Tc_d^{\sigma ,\epsilon ,T}-\partial _x\Phi ^T\Omega _d^{\sigma ,\epsilon ,T}\Big ]{\mathrm d}s'\\&-\frac{\sigma ^2}{2}\int _0^se^{{\mathcal {L}}_{c(T)}(s-s')}\Big [\partial ^2_c\Phi ^T\Vert Q^{1/2}c_s^{T}\Vert _{L^2}^2+\partial ^2_x\Phi ^T\Vert Q^{1/2}\xi _s^{T}\Vert _{L^2}^2\Big ] {\mathrm d}s'\\&-\sigma ^2\int _0^se^{{\mathcal {L}}_{c(T)}(s-s')}\partial ^2_{xc}\Phi ^T\langle Q^{1/2}c_s^{T},Q^{1/2}\xi _s^T\rangle {\mathrm d}s'\\&-\sigma \int _0^se^{{\mathcal {L}}_{c(T)}(s-s')}\partial _c\Phi ^T\langle c_s^{T},T_{\xi (T)+c(T)s'}{\mathrm d}W^Q_{T+s'}\rangle \\&-\sigma \int _0^se^{{\mathcal {L}}_{c(T)}(s-s')}\partial _x\Phi ^T\langle \xi _s^{T},T_{\xi (T)+c(T)s'}{\mathrm d}W^Q_{T+s'}\rangle \\&+\sigma \int _0^s e^{{\mathcal {L}}_{c(T)}(s-s')}[\Phi ^T+v^T] T_{\xi (T)+c(T)s'}{\mathrm d}W^Q_{T+s'}, \end{aligned}$$

as desired. □

### Proof of Theorem 5.3

Applying [24, Theorem 4.5] gives

$$\begin{aligned} \mathbb {E}\sup _{t\in [0,T]} \big \Vert \int _0^t S(t-s)g(s){\mathrm d}W^Q_s \big \Vert _{{\mathcal {H}}}^p\le (K_p M\sqrt{p})^p T^{\frac{p}{2}-1}\int _0^T\mathbb {E}\Big [ \Vert g(t)\Vert ^p_{\textrm{HS}(L_Q^2,{\mathcal {H}})}\Big ]{\mathrm d}t , \end{aligned}$$

for $p\in (2,\infty )$, where $\limsup _{p\rightarrow \infty }K_p<\infty$. Assume without loss of generality that $K_p\le K$ for p>2. By our assumption,

$$\begin{aligned} \mathbb {E}\sup _{t\in [0,T]} \big \Vert \int _0^t S(t-s)g(s){\mathrm d}W^Q_s \big \Vert _{{\mathcal {H}}}^p\le (BK M\sqrt{p}\sqrt{T})^p , \end{aligned}$$

for p>2. Markov’s inequality then gives

$$\begin{aligned} \mathbb {P}\Big [ \sup _{t\in [0,T]}\big \Vert \int _0^t S(t-s)g(s){\mathrm d}W^Q_s \big \Vert _{{\mathcal {H}}}^p \ge \lambda ^p \Big ] \le (\lambda ^{-1}BK M\sqrt{p}\sqrt{T})^p . \end{aligned}$$

For $\lambda > eBKM\sqrt{ T}$ we may choose $p=(eBK M)^{-2}\lambda ^2/T$ to conclude

$$\begin{aligned}  &   \mathbb {P}\Big [ \sup _{t\in [0,T]}\big \Vert \int _0^t S(t-s)g(s){\mathrm d}W^Q_s \big \Vert _{{\mathcal {H}}} \ge \lambda \Big ]\\  &   \quad =\mathbb {P}\Big [ \sup _{t\in [0,T]}\big \Vert \int _0^t S(t-s)g(s){\mathrm d}W^Q_s \big \Vert _{{\mathcal {H}}}^p \ge \lambda ^p \Big ] \le e^{-(eBKM)^{-2}\lambda ^2/T}.\end{aligned}$$

□

## Footnotes

1 The process $c_{\textrm{ap}}$ introduced in (1.7) approximates the soliton amplitude only in distribution. As will be made clear later on, the soliton amplitude is driven by a translated version of $W_t^Q$.
